# Investigating the Effects of Electron Correlation and Methylation on the *S*_1_/*S*_0_ Conical Intersection Seam of Fulvene

**DOI:** 10.1021/acs.jctc.5c01276

**Published:** 2025-12-27

**Authors:** Javier Segarra-Martí, Michael J. Bearpark

**Affiliations:** † Instituto de Ciencia Molecular, 16781 Universitat de Valéncia, C/Catedrático José Beltrán 2, 46980 Paterna, Valencia, Spain; ‡ Department of Chemistry, Molecular Sciences Research Hub, 4615 Imperial College London, White City Campus, 82 Wood Lane, W12 0BZ London, U.K.

## Abstract

The benzene isomer fulvene has established itself as a computational benchmark for characterizing conical intersections of potential energy surfaces and modeling radiationless decay. However, in contrast to other benchmark systems such as benzene itself or the DNA/RNA nucleobases, there is as yet no time-resolved experimental data for fulvene to compare computational predictions with. This article has three main aims. The first is to thoroughly characterize the *S*_1_/*S*_0_ conical intersection seam of fulvene mediating ultrafast decay along its main reaction coordinates. The second is to understand how radiationless decay is shaped by the electron correlation included in the model and is carried out by comparing XMS-CASPT2 with CASSCF results and extracting how dynamic correlation effects impact differently the critical points encountered along the seam. The third and final aim is to compare fulvene against 6-methyl-fulvene (6MFulv) and 6,6-dimethyl-fulvene (DMF), potential surrogate systems available experimentally. We find methylation does not alter the energetics along the majority of the conical intersection seam itself, while single methylation (6MFulv) enables easier access to the planar region of the seam through the C_1_–C_6_ stretching coordinate compared to the parent fulvene or DMF. We thus suggest DMF as an appropriate surrogate system accessible by current experimental setups and where predictions on fulvene excited state reactivity can be tested.

## Graphical abstract

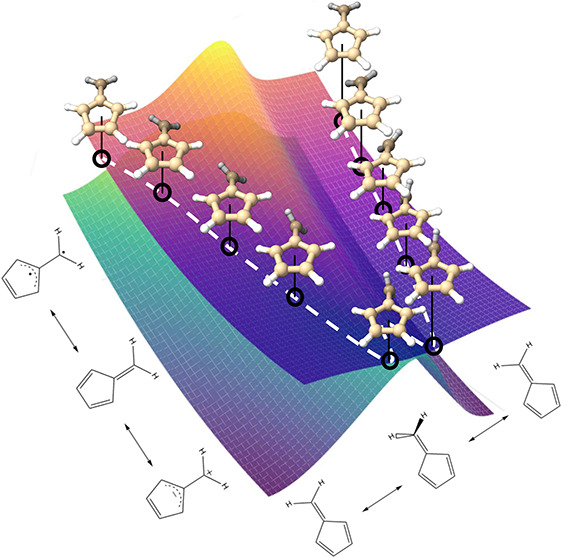

## I Introduction

One of the most notorious examples in molecular photophysics is that of azulene, which was the very first system known to display what was referred to as “anomalous fluorescence,”^1^ where emission occurs from an upper excited state instead of the lowest-lying in energy (i.e., *S*_1_),^2^ going against the well-known Kasha rules.^3^ As such, this molecule plays an important part in the development of our understanding of radiationless decay.

At the end of the article by Beer and Longuet Higgins describing this phenomenon,^1^ they wrote it was also impossible to obtain emission from the first excited state of another system, 6,6-dimethyl-fulvene. The implication here was clear: there must be a connection between these two nonradiative species, which was later on reconciled with the first conical intersection characterization in azulene mediating internal conversion 40 years later.^4,5^

These calculations brought together a body of experimental evidence built over the years, including the work of Rentzepis^6–8^ and later Zewail,^9^ in which they recorded an emission feature arising from the *S*_2_ electronic excited state. These helped extend our understanding of nonadiabatic crossings and their relevance to reactivity, highlighting their widespread occurrence and challenging the back-then prevalent view of surface crossings as rare, with little impact in photochemistry.

Fulvene^10,11^ has recently established itself as one of the preferred benchmark model systems to study nonadiabatic processes. It is an isomer of benzene which features an azulene-like conical intersection that can, in principle, mediate an ultrafast radiationless deactivation to the electronic ground state.^5^ Presently, there are still no comparable time-resolved experiments for fulvene, despite featuring very interesting photophysical and photochemical properties and being a unique model to study efficient internal conversion processes across a wide range of crossing points.

Substantial work has been carried out theoretically to characterize nonadiabatic events in fulvene.^5,12–27^ Interestingly, and despite featuring an azulene-like planar crossing, this is not the minimum energy conical intersection (MECI) but rather a critical point along the intersection seam, the MECI requiring an additional CH_2_ torsion that hampers its accessibility dynamically.

All of the above make fulvene an ideal prototype for two concepts that emerged later on: (i) radiationless decay most likely away from the MECI (though it is targeted directly following evolution from the Franck–Condon (FC) point, unlike other systems), and (ii) an extended seam of crossing, with multiple critical points, and having different topographies, i.e., sloped vs peaked.^14^

Fulvene has recently experienced a resurgence in popularity as a molecular surrogate for the Tully wave packet reflection model^28^ to assess nonadiabatic dynamics schemes.^15^ This confers added value to this system, as it is used to benchmark new nuclear propagation schemes,^15–18,20,27,29^ and a thorough understanding of its potential energy surface is therefore essential.

An aspect that has not been thus far thoroughly considered to our knowledge (in fulvene; it has been addressed previously for ethylene^30^) is how electron correlation affects this photoprocess, and particularly the *S*_1_/*S*_0_ conical intersection seam that dominates deactivation from *S*_1_. To assess this, here we use complete active space self-consistent field (CASSCF) calculations^5^ and compare them against a range of multistate complete active space second-order perturbation theory (CASPT2) approaches, which account for missing dynamic electron correlation.

Our results point to subtle yet important changes in the potential energy landscape of fulvene due to (dynamic) electron correlation. We show it substantially reduces the vertical excitation energy, in agreement with available experimental evidence,^31–34^ while not having a prominent effect on the resulting molecular geometries (ground and excited state minima, as well as MECIs). We also show dynamic electron correlation affects the conical intersection seam and the neighboring conformational space in subtle yet important ways: (i) it favors access to more twisted conical intersections compared to CASSCF estimates, and modifies the range of twisted structures for which conical intersection topography changes from sloped to peaked, (ii) it hampers access to the more planar section of the crossing seam, which dominates decay in CASSCF-based nonadiabatic dynamics simulations,^5^ and (iii) it reduces the norm of the nonadiabatic coupling (and of the molecular gradients) across the potential energy landscape, which is likely to hamper/delay nonadiabatic events.

We also analyze the *S*_1_/*S*_0_ intersection seam of 6-methyl- (6MFulv) and 6,6-dimethyl-fulvene (DMF) derivatives, which are both commercially available and thus readily measurable. We show that the excited state potential energy landscape of 6MFulv deviates from fulvene at planar structures, which we expect may enhance decay through the C_1_–C_6_ stretching coordinate, while DMF remains qualitatively unchanged, with the intersection seam of both methylated species away from the planar region being almost quantitative to that of fulvene. We therefore suggest DMF as the most appropriate fulvene surrogate to measure experimentally.

## II Computational Details

All calculations reported were carried out using the OpenMolcas package.^35–37^ The Cholesky decomposition was used to speed up electron repulsion integrals,^38–40^ and the Atomic Natural Orbital large (ANO-L)^41^ one-electron basis set was employed throughout in its double-ζ contraction.

Complete active space self-consistent field (CASSCF) reference calculations in fulvene included 3 each of occupied and nonoccupied π orbitals, resulting in 6 electrons in 6 orbitals for CASSCF­(6,6), as shown in Figure S1 of the Supporting Information (SI). An equal-weight state-averaging procedure was used, including the lowest 2 electronic states. An additional set of calculations averaged over 5 roots was also performed on fulvene to assess the potential interaction of the *S*_2_ state along the *S*_1_/*S*_0_ intersection seam (included in the SI, Section S3).

On top of the CASSCF reference functions, second-order perturbation theory (CASPT2)^42^ corrections were carried out using multistate (MS),^43^ extended multistate (XMS)^44,45^ and dynamically weighted and rotated multistate (XDW and RMS)^46,47^ zeroth-order Hamiltonians, with only XMS-CASPT2 estimates (hereafter referred to as CASPT2) being reported in the main text while MS/RMS/XDW-CASPT2 results, are provided in the SI. Any significant differences between XMS and any other CASPT2 formulation are highlighted in the text with reference to the SI where relevant. A 0.2 au imaginary level shift^48^ was employed to avoid the presence of intruder states, and the IPEA shift was set to zero.^49^

First-order energy derivatives and nonadiabatic couplings for CASSCF and the different multistate CASPT2 variants were used as implemented in OpenMolcas.^50–52^ Using these, ground and excited state optimizations were characterized. Conical intersection searches were performed using the method of Fdez Galván et al.^53^

Two-dimensional (2D) maps corresponding to a rigid scan along the C_1_–C_6_ bond and C_2‑_C_1_–C_6‑_H_7_ torsion coordinates and starting off with the reference (*S*_1_)_min_ XMS-CASPT2 structures were generated using ChemCoord,^54^ and are reported in Figures 2 and 6.

For mapping the conical intersection seam along the twisting coordinate, a constrained optimization procedure was set up so that the CH_2_ torsion angle was constrained every 10° while enforcing ring planarity and a minimum energy conical intersection (MECI) search. Additional results without ring planarity constraints and the slight changes observed are reported in the SI. The intersection parameters P and B defined by Fdez Galván et al.^53^ were obtained for each MECI and used to classify the different conical intersection points along the seam, discerning those with peaked ( P<1 ) from those with sloped ( P>1 ) topography, as well as those single-pathed ( B>1 ) from those bifurcating ( B<1 ).^55^

## III Results and Discussion

Results are organized as follows: we first analyze changes incurred by ground and excited state minima (both geometries and energies; Section 3.1) upon including dynamic electron correlation by comparing CASSCF with XMS-CASPT2 (labeled as CASPT2 from there onward; other multistate formulations and an analysis of the small changes observed between them are reported in the SI) and then move onto the role of correlation on seam topology (Section 3.2) and how it affects its accessibility (Section 3.3), finishing with a detailed discussion on how these are affected due to methylation (Section 3.4).

### III.I Critical Points Along the PES

We start by considering the differences induced by electron correlation in the relative energies of the critical points characterized along the *S*_1_ excited state decay of fulvene. Table [Table tblI] shows the relative (adiabatic, with respect to *S*_0_ at (*S*_0_)_min_) energies of the different critical points at the CASPT2 and CASSCF levels of theory. As can be seen, a pronounced (∼0.7 eV) decrease of the *S*_0_ → *S*_1_ transition is observed at (*S*_0_)_min_ upon including dynamic electron correlation leading to an estimate for the vertical excitation energy of 3.25 eV (vs the 4.03 eV predicted at CASSCF, see Table [Table tblI]), which more closely resembles the absorption maximum registered experimentally in the gas phase at 3.44 ^33,34^ and 3.34 eV.^31^

**1 tblI:** CASPT2 Adiabatic Energies (Δ*E*, in eV) of Fulvene for the Different Critical Structures Characterized in This Work; CASSCF Energy Estimates Are Provided in Brackets

state	(*S*_0_)_min_	(*S*_1_)_min_	(*S*_1_/*S*_0_)_MECI_	(*S*_1_/*S*_0_)_CI_^0°^
*S*_0_	0.00 (0.00)	0.90 (1.41)	2.38 (2.43)	3.01 (2.85)
*S*_1_	3.25 (4.03)	2.38 (2.58)	2.38 (2.43)	3.01 (2.85)

Along the relaxation pathway of the *S*_1_ state, energy differences become gradually smaller, being only 0.2 eV between CASSCF and CASPT2 estimates at (*S*_1_)_min_, and 0.05 eV at (*S*_1_/*S*_0_)_MECI_. A qualitative difference, however, shows between CASSCF and CASPT2 results: CASSCF places this conical intersection energetically (by 0.15 eV) below (*S*_1_)_min_, whereas CASPT2 places it at very close energies. This all points to minor differences in predicted *S*_1_ → *S*_0_ population transfer due to the electron correlation retained in the model, which may be slightly more accessible at CASSCF^5,15–17,20^ but that is also effectively barrierless at CASPT2. Larger differences are, however, found for the relative energetic position of the planar conical intersection (*S*_1_/*S*_0_)_CI_^0°^, which is increased by 0.16 eV to higher energies at CASPT2 and whose accessibility we discuss below.

We consider the changes in fulvene critical points (see Figure 1), that is, (*S*_0_)_min_, (*S*_1_)_min_, (*S*_1_/*S*_0_)_MECI_, and (*S*_1_/*S*_0_)_CI_^0°^, at CASSCF and upon including dynamic electron correlation by means of the CASPT2 method next. It is worth noting that we use (*S*_1_/*S*_0_)_MECI_ for the unconstrained minimum energy conical intersection, which is twisted for all levels of theory tested, whereas (*S*_1_/*S*_0_)_CI_^0°^ denotes the planar conical intersection that is only obtained upon imposing constraints, as it lies at higher energies.

**Figure 1:**
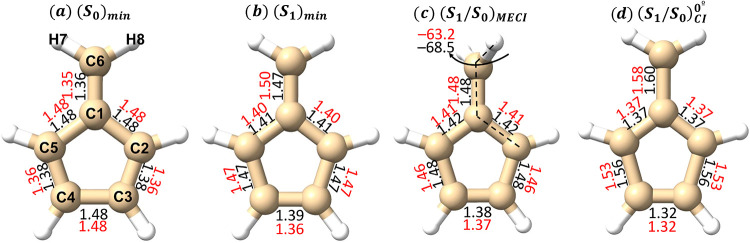
Molecular geometries of (a) (*S*_0_)_min_, (b) (*S*_1_)_min_, (c) (*S*_1_/*S*_0_)_MECI_ and (d) (*S*_1_/*S*_0_)_CI_^0°^, together with their (relevant) atom labeling. Bond length distances are provided in Å: red for CASSCF and black for CASPT2 optimized structures.

The resulting optimized geometries for the different critical points are for the most part relatively unaffected by the level of theory employed, as shown in Figure 1, even if there are some small differences worth highlighting. A clear pattern is observed in (*S*_0_)_min_ where fulvene retains a planar structure and where C_4‑_C_5_, C_2‑_C_3_, and C_1_–C_6_ display short double-bond distances, while the rest are significantly longer. This picture changes upon relaxation to (*S*_1_)_min_, with a pronounced C_1_–C_6_ elongation (∼0.12 Å) and C_3‑_C_4_ shortening (∼0.10 Å) observed for both CASPT2 and CASSCF, in line with previous CASSCF estimates^5^ and with concomitant changes to all other bond lengths while preserving planarity.

Further relaxation on *S*_1_ leads to (*S*_1_/*S*_0_)_MECI_, where both methods show an elongated C_1_–C_6_ bond length coupled with a H_7_C_6_C_1_C_2_ torsion motion that appears to be favored slightly at CASPT2, displaying a more twisted angle. Fewer differences are observed for (*S*_1_/*S*_0_)_CI_^0°^, which corresponds to the conical intersection featuring a planar geometry and characterized by a significantly larger C_1_–C_6_ stretching, which is slightly more pronounced at CASPT2, reaching 1.6 Å, and where both CASSCF and CASPT2 display almost identical structures.

After populating the *S*_1_ excited state, we see a pronounced C_1_–C_6_ elongation (see Figure 1) leading to (*S*_1_)_min_ and a concomitant relaxation in the *S*_1_ excited state energy, which decreases by 1.45 and 0.87 eV for the CASSCF and CASPT2 levels of theory, respectively. From (*S*_1_)_min_, there is a potential energy barrier to be surmounted to reach the planar (*S*_1_/*S*_0_)_CI_^0°^ mediating decay to the ground state along the C_1_–C_6_ stretching coordinate (see Table [Table tblI]), placed 0.27 and 0.63 eV above the excited state minimum for CASSCF and CASPT2, respectively. This energy barrier thus depends on the amount of electron correlation retained in the model, being the largest for the CASPT2 computation, which is more than twice that of the CASSCF result: dynamic electron correlation therefore appears to disfavor decay along the C_1_–C_6_ stretching coordinate at 0° torsion.

Summarizing, dynamic electron correlation appears to be crucial to appropriately model the absorption maximum of fulvene but also leads to significant differences in terms of potential energy barriers to be surmounted to access the planar (*S*_1_/*S*_0_) conical intersection seam mediating *S*_1_ → *S*_0_ decay to the ground state. The effects of electron correlation on the molecular structure appear to be quite small.

### III.II Seam Topography along the CH_2_ Torsion

Previous work by Bearpark, Robb and co-workers has extensively studied the conical intersection seam of fulvene,^5,14,21–23,56^ and how this seam may affect subsequent nonadiabatic molecular dynamics with different nuclear propagation methods.^5,12,13,57^ Despite the vast body of work available on the topic,^15–18,20,27,29^ almost none of these dynamics studied included dynamic electron correlation in the model.^27^

As a first step, we explore the potential energy landscape around (*S*_1_)_min_ along the C_1_–C_6_ stretching and CH_2_ torsion. An *S*_1_ relaxed scan was also computed, leading to energy differences that are qualitatively equivalent to those shown here for the rigid scan (see Figure S4 in the SI). From (*S*_0_)_min_ to (*S*_1_)_min_ fulvene experiences very significant structural changes,^5^ triggering an ∼0.1 Å shortening of the C_3‑_C_4_ and lengthening of the C_4‑_C_5_, C_2‑_C_3_ and C_1_–C_6_ bonds (see Section 3.1), effectively alternating the single to double bond length pattern.

Figure 2a,b shows energy degeneracies from ∼80/∼100° (∼70/∼110°) at very short C_1_–C_6_ distances, moving onto lower/higher torsion angles as the bond elongates and reaching values of ∼60/∼120° (∼40/∼140°) for CASPT2 (CASSCF) levels of theory, respectively. CASSCF therefore favors degeneracies along less twisted geometries, while the introduction of electron correlation appears to favor torsion: CASPT2 estimates show large energy differences (∼1 eV) across most of the space covered here, while CASSCF shows very low *S*_1_–*S*_0_ energy gaps even at 0° torsion when the C_1_–C_6_ bond is sufficiently elongated (>1.7 Å).

**Figure 2:**
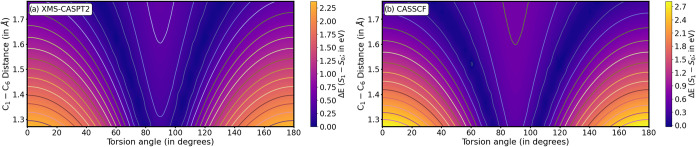
Potential energy cuts for the rigid scan along the torsion (*X*-axis; in degrees) and C_1_–C_6_ stretching (*Y*-axis; in Å) for (a) CASPT2 and (b) CASSCF levels of theory in fulvene starting from the (*S*_1_)_min_ optimized structure (further details are provided in the Section 2). The *Z*-axis represents a color map showing the *S*_1_–*S*_0_ energy difference (in eV), with the same color range applied to both panels for comparison.

From the rigid scans, we predict CASSCF to show faster decays than CASPT2 and to favor decay through more planar structures. This depends on two aspects: (i) the significantly lower energy gaps displayed across the explored geometric space, especially at highly elongated C_1_–C_6_ bond lengths and small torsion angles, featuring energy gaps sufficiently low as to enable population transfers at planar geometries, and (ii) an estimated ∼30% larger nonadiabatic coupling and molecular gradient norm across the whole geometry space explored (more details in the SI Figures S5 and S6), which is expected to increase population transfer.

With this, we explore the actual *S*_1_/*S*_0_ conical intersection seam along the torsion coordinate, which is expected to be responsible for the *S*_1_ → *S*_0_ decay.^5^ In Figure 3, we show how the CASPT2 characterization of the *S*_1_/*S*_0_ conical intersection seam leads to very similar results to those previously found^5,14^ (and here recomputed) at CASSCF: both energy profiles in Figure 3a show equivalent twisted crossing minima (or CI_min_ by Sicilia et al.^14^), the main differences arising at the planar crossing where CASPT2 is placed at significantly higher energies as previously discussed (see Table [Table tblI]; while featuring an analogous structure to CI_plan_ by Sicilia et al.^14^ and also to those recently obtained by Wang et al.,^58^ our estimates being shown in Figure 1). Conical intersection topology along the seam is analyzed in Figure 3b, showing how the seam smoothly transitions from sloped ( P>1 ) to peaked ( P<1 ) intersections as it approaches 90° torsion (or CI_perp_ by Sicilia et al.^14^) and thus supporting a single conical intersection seam connecting all points^14^ instead of the existence of two separate seams as suggested by Deeb et al.^59^ The associated B values are reported in the SI Figure S3, together with an analysis of the multistate treatment and how it affects the resulting conical intersection topology: we observe a single-path behavior (i.e., B>1 ) across the seam with the exception of the 90° structure, and significant differences in the resulting topologies particularly for MS- and XDW-CASPT2 formulations. A thorough discussion is provided in the SI.

**Figure 3:**
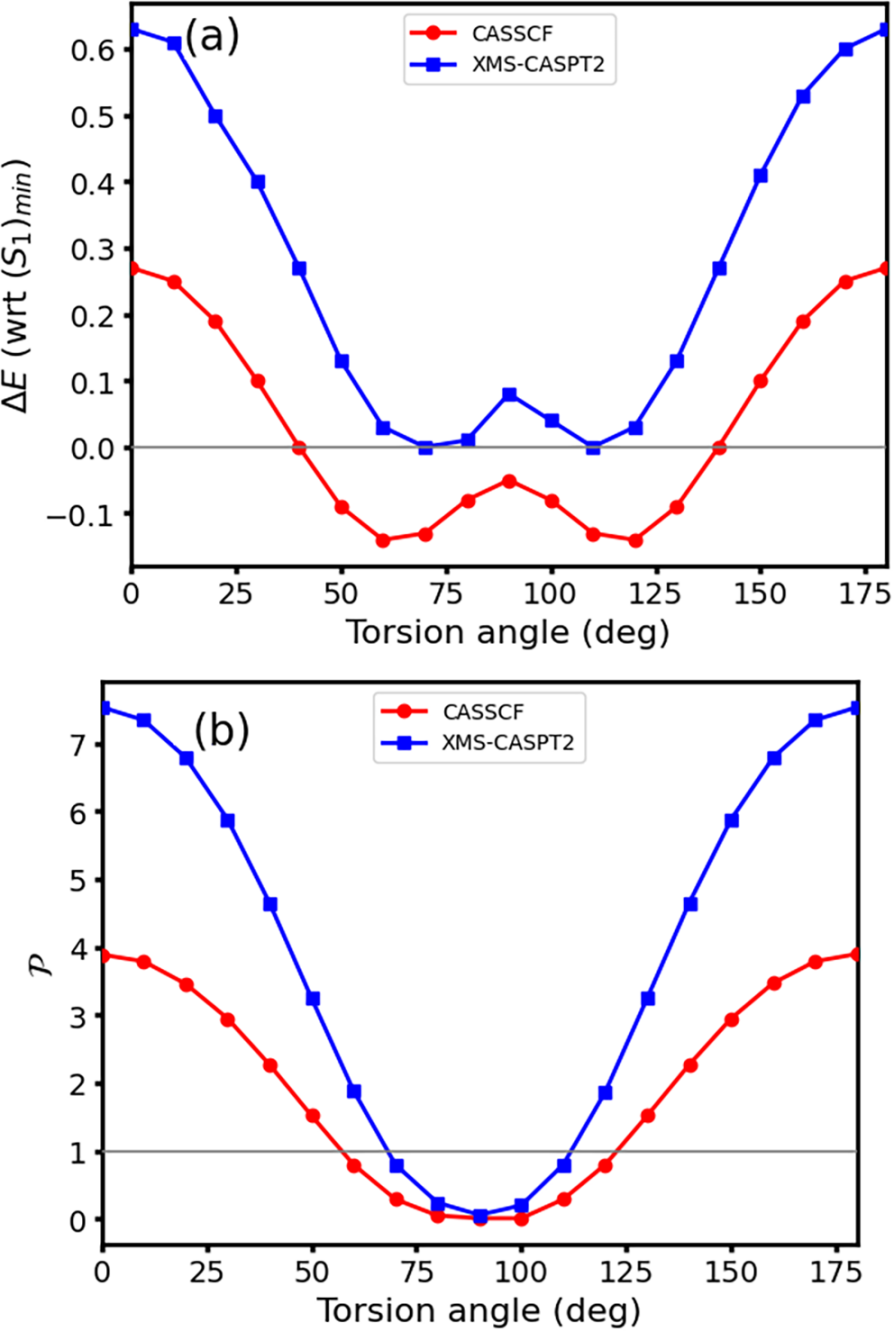
Constrained optimization of the *S*_1_/*S*_0_ conical intersection seam of fulvene along the CH_2_ torsion. (a) Adiabatic energy (in eV, with respect to (*S*_1_)_min_) of the seam for CASSCF (red) and CASPT2 (blue). The horizontal gray line showcases Δ*E* = 0. (b) Conical intersection parameter P . The horizontal gray line showcases P=1 which is used to classify sloped ( P>1 ) vs peaked ( P<1 ) intersections.^53^

We have also considered the potential role of an ionic *S*_2_ state in the photoprocess at highly twisted structures where it is heavily stabilized, a possibility mentioned but that could not be accurately assessed in previous work.^5^ Over the range of geometries scanned, driven by *S*_1_ evolution, we do not find any *S*_2_/*S*_1_ crossings; a recomputed seam including 5 states in the state-averaging procedure (and therefore including *S*_2_) can be found in the SI Section S3. We also monitored changes in the molecular charge of the cyclopentadienyl and methylene fragments along the seam, finding no qualitative differences to arise due to the inclusion of dynamic electron correlation (SI Section S4).

Changes in the CASPT2 bond lengths along the intersection seam are reported in Figure 4. It can be seen that all bond lengths in the ring become comparable for 90° CH_2_ torsion, in agreement with previous work on the cyclopentadienyl structure.^60–62^ The C_1_–C_6_ distance remains elongated, compared to (*S*_0_)_min_ but at its shortest length along the *S*_1_/*S*_0_ intersection seam, with the biggest shortening being observed for the C_3‑_C_4_ bond for planar structures.

**Figure 4:**
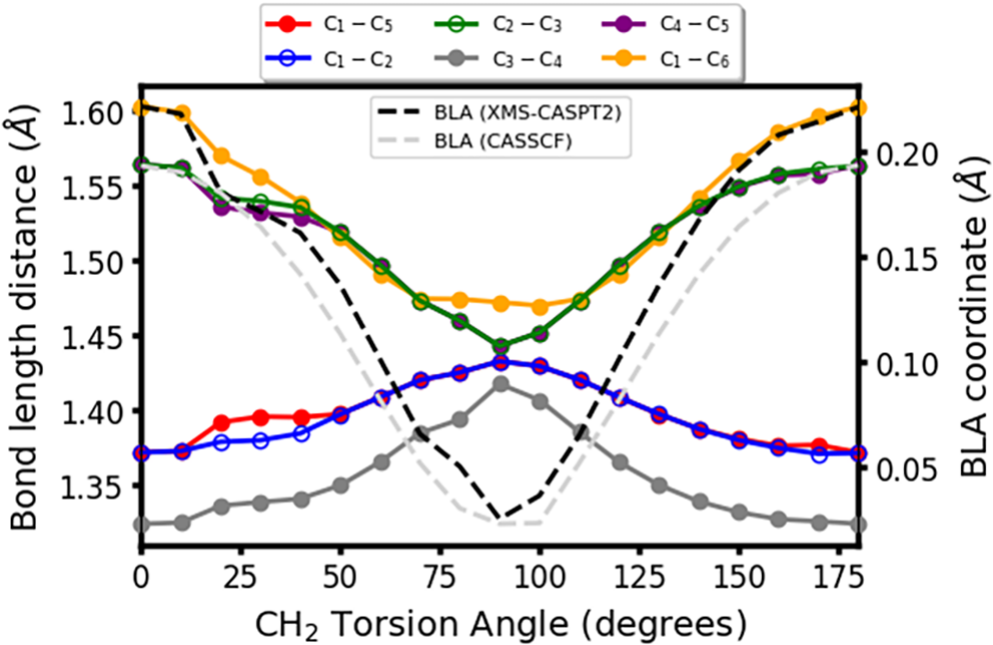
CASPT2 changes in C–C bond length distances (Å) along the intersection seam. The bond length alternation (BLA) coordinate is represented on the right-hand side *y*-axis and is displayed by black (CASPT2) and light gray (CASSCF) dashed lines.

To better observe this trend, we look at the bond length alternation (BLA) coordinate, which measures the difference between the average bond length distance in single and double bonds, considering them single or double with respect to their ground state equilibrium geometry (see Figure 1), and that is represented in the right-hand vertical axis of Figure 4. Black (CASPT2) and gray (CASSCF) dashed lines represent the values obtained for the BLA coordinate along the seam, which shows a noticeable alternating single/double-bond character for planar structures while displaying very small values (i.e., very small differences between single and double bonds) as CH_2_ twisting approaches 90°, where the BLA coordinate is almost zero, as expected in structures featuring almost identical bond lengths, and which is depicted by both levels of theory.

### III.III Accessibility of the Conical Intersection Seam

In Section 3.2, we analyzed in detail the energetics at and around the characterized *S*_1_/*S*_0_ conical intersection seam as well as its topography. In this section, we look instead at a crucial aspect to understand fulvene photophysics, which is how accessible and therefore photochemically relevant the MECIs characterized are.

To assess the accessibility of the MECIs encountered along the *S*_1_/*S*_0_ torsion conical intersection seam, we compute a linear interpolation in internal coordinates (LIIC) between (*S*_1_)_min_ and the MECIs at varying torsion angles. It is worth noting that LIICs are known to provide upper bounds rather than accurate estimates for the actual potential energy barriers, and as such, the values themselves might not be completely trustworthy; nevertheless, the approximation is equally applied to both CASSCF and XMS-CASPT2 approaches, and the analysis of their differences should still remain meaningful. We start LIICs from (*S*_1_)_min_, as initial relaxation in fulvene is a C_1_–C_6_ bond elongation^5,12,15–20,57^ and C_3‑_C_4_ shortening embodied by (*S*_1_)_min_ and leading to a bond length alternation as seen in Figure 1.

As can be seen in Figure 5, MECIs featuring small or no torsion in the 0–40° range appear at higher energies but otherwise feature no distinguishable potential energy barrier along the interpolated path. MECIs with larger torsions (50–90°), on the other hand, appear at noticeably lower energies (see the (*S*_1_/_S0_)_CI_ estimates on the right-hand side of each panel in Figure 5) but feature a sizable potential energy barrier to access them. This shows that the *S*_1_ energy increases along the CH_2_ torsion motion, thus making decay along less twisted structures more prevalent, even if these are placed at higher energies. This is in line with previous nonadiabatic molecular dynamics predictions.^5^

**Figure 5:**
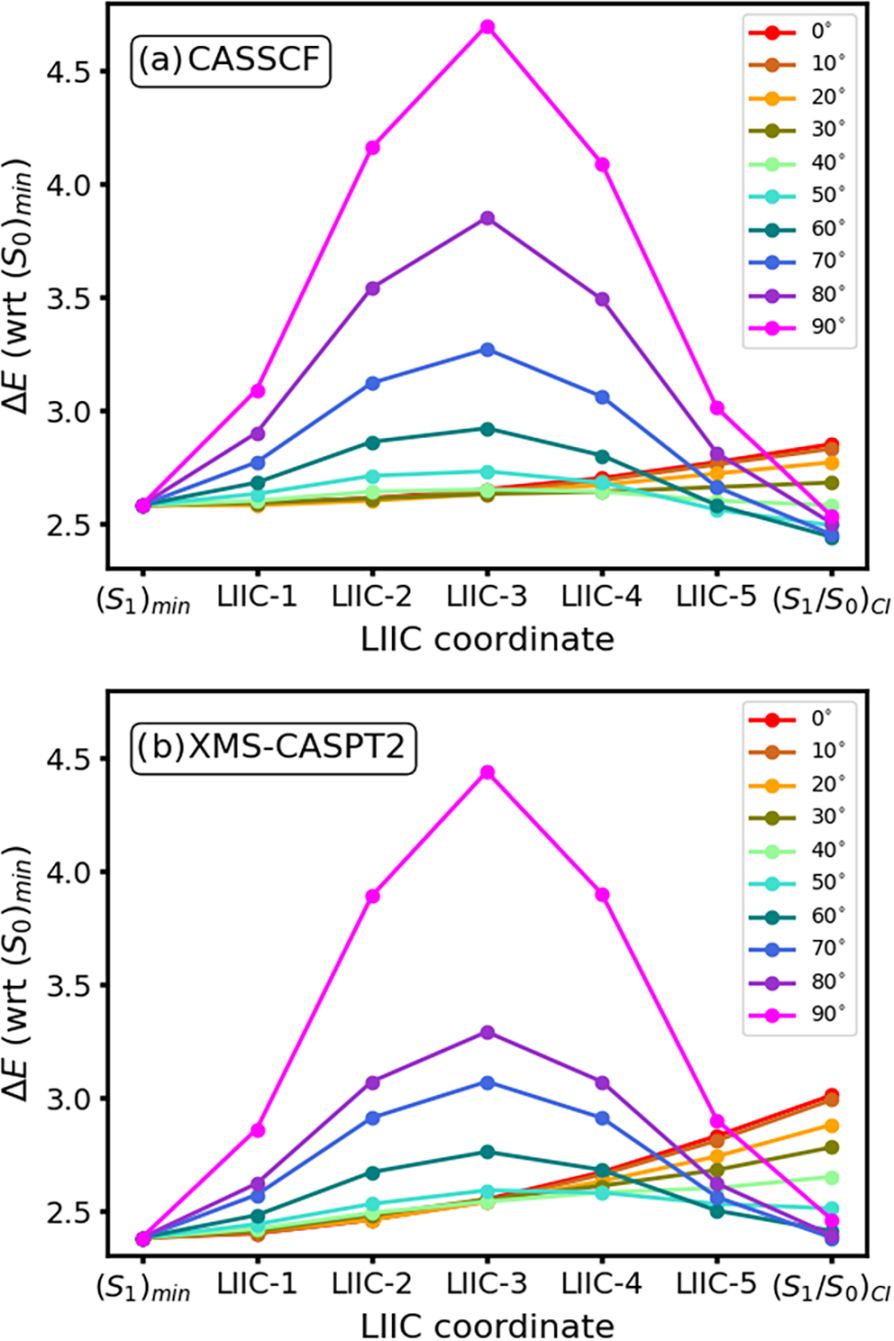
Linear interpolation in internal coordinates (LIIC) between (*S*_1_)_min_ and the different minimum energy conical intersection points characterized along the seam in the 0–90° range described by the CH_2_ torsion mode for (a) CASSCF and (b) CASPT2 levels of theory. Energies are referred to adiabatic *S*_1_ values (i.e., with respect to the ground state minimum (*S*_0_)_min_) and are reported in eV.

Dynamic electron correlation plays a subtle role in modulating access to intersections with varying torsion angles: upon comparing Figure 5a,b, we observe smaller potential energy barriers for the more twisted pathways (torsion angles 50–90°) at CASPT2, while at the same time displaying higher energies for the planar MECIs (torsion angles 0–40°). This suggests CASPT2 may favor larger torsions compared to CASSCF, even if highly twisted (70–90° torsion) structures are still unfavorable at both levels of theory. Concretely, Figure 5a shows the 0–40° torsion MECIs to be relatively accessible at CASSCF: 0, 10, 20, 30 and 40° MECIs feature potential energy barriers of 0.27, 0.25, 0.19, 0.1, and 0.07 eV, respectively, compared to 0.58, 0.56, 0.49, 0.41, and 0.27 eV for CASPT2 (cf. Figure 5b). CASPT2, on the other hand, shows the lowest potential energy barrier along the path connecting (*S*_1_)_min_ to the MECI at 50° at 0.21 eV.

### III.IV Single and Double Methylations

As fulvene methylated species are available commercially, we characterize the *S*_1_ → *S*_0_ decay in 6-methyl-fulvene (6MFulv) and 6,6-dimethyl-fulvene (DMF) to lay the groundwork for further experimental validation. We focus here on the discussion of the CASPT2 results.

Tables [Table tblII] and [Table tblIII] show the effects of methylation on the vertical excitation energies and main geometric parameters of the key structures encountered along the nonradiative decay, respectively.

**2 tblII:** Adiabatic Energies of 6-Methyl-fulvene (6MFulv) and 6,6-Dimethyl-fulvene (DMF) for the Different Critical Structures Characterized in This Work; the Values Reported (in eV) Are Computed at the CASPT2 Level of Theory, with CASSCF Energy Estimates in Brackets for Comparison

system	state	(*S*_0_)_min_	(*S*_1_)_min_	(*S*_1_/*S*_0_)_MECI_	(*S*_1_/*S*_0_)_CI_°
6-methyl-fulvene (6MFulv)	*S*_0_	0.00 (0.00)	0.89 (1.41)	2.40 (2.42)	3.18 (2.94)
	*S*_1_	3.32 (4.10)	2.46 (2.64)	2.40 (2.42)	3.18 (2.94)
6,6-dimethyl-fulvene (DMF)	*S*_0_	0.00 (0.00)	0.86 (1.39)	2.39 (2.38)	3.32 (2.99)
	*S*_1_	3.34 (4.10)	2.50 (2.66)	2.39 (2.38)	3.32 (2.99)

**3 tblIII:** Main Structural Parameters (Atom Labeling in Figure 1) for the Key Structures Encountered along the *S*_1_ Decay in 6-Methyl-fulvene (6MFulv) and 6,6-Dimethyl-fulvene (DMF), Computed at the CASPT2 Level of Theory (CASSCF Estimates in Brackets)[Table-fn tIIIfn1]

	system	C_1_–C_6_ (Å)	H_7‑_C_6‑_C_1_–C_2_ (°)
	fulvene	1.364	–0.015
(*S*_0_)_min_	6MFulv	1.366	–0.021
	DMF	1.370	–0.026
	fulvene	1.471	–0.004
(*S*_1_)_min_	6MFulv	1.473	0.001
	DMF	1.478	–0.042
	fulvene	1.476	–68.386
(*S*_1_/*S*_0_)_MECI_	6MFulv	1.474	65.205
	DMF	1.476	57.021
	fulvene	1.602	–0.050
(*S*_1_/*S*_0_)_CI_^0°^	6MFulv	1.623	0.296
	DMF	1.643	1.181

a Fulvene is also reported for comparison. Bond distances are measured in Å, while torsion and angles are measured in degrees. Torsion refers to C–C_6‑_C_1_–C_2_ for both methylated species, and the angle to C–C_6‑_H_8_ for 6MFulv and to C–C_6‑_C in DMF.

We predict methylation to blue-shift the vertical absorption very slightly around the FC region, moving it from 3.25 eV for fulvene (cf. Table [Table tblI]) to 3.32 eV upon single and to 3.34 eV upon double-methylation (cf. Table [Table tblII]), both increases being less than 0.1 eV away from the fulvene reference. Similarly to fulvene, our CASPT2 predictions for 6MFulv and DMF absorption are in agreement with the experimentally recorded absorption maxima at 3.34 ^63^ and 3.35 eV^31,32^ in the gas phase, respectively, and also with the more recent VUV absorption measurement of ∼3.5 eV by Palmer et al. for DMF.^64^

As can be seen in Table [Table tblII], methylation results in relatively small energy changes when compared to fulvene: the adiabatic energies observed at (*S*_1_)_min_ (2.46 and 2.50 eV for 6MFulv and DMF, respectively, vs 2.38 eV for fulvene, Table [Table tblI]) are slightly bigger than those predicted for (*S*_0_)_min_ (3.32 and 3.34 eV for 6MFulv and DMF, respectively, vs 3.25 eV for fulvene) but still represent very small changes that are unlikely to affect excited state decay prominently. Despite the small but noticeable changes observed in (*S*_0_)_min_ and (*S*_1_)_min_, the minimum energy conical intersection (*S*_1_/*S*_0_)_MECI_ is placed adiabatically at effectively the exact energy for all systems (2.38–2.39 eV), suggesting that this structure is electronically unaffected by methylation.

The planar intersection (*S*_1_/*S*_0_)_CI_^0°^ is much more affected upon methylation: we observe a 0.17 eV shift to higher energies for 6MFulv and 0.31 eV for DMF compared to fulvene. These differences are also reflected in the potential energy barrier to be surmounted in order to decay through the C_1_–C_6_ stretching coordinate by means of (*S*_1_)_min_ → (*S*_1_/*S*_0_)_CI_^0°^, which goes from 0.63 eV for fulvene (see Table [Table tblI]) to 0.72 and 0.82 eV for 6MFulv and DMF, respectively.

We predict methylation to induce very small differences in terms of the molecular structure. Table [Table tblIII] shows the main internal coordinates activated along decay, that is, the C_1_–C_6_ bond distance and CH_2_ torsion (and the analogues for methylated species). The only difference worth mentioning is the smaller torsion angle predicted for (*S*_1_/*S*_0_)_MECI_ in DMF, which deviates ∼10° from the parent fulvene structure.

We look next at the energies around the main C_1_–C_6_ stretching and CH_2_ torsion coordinates, displayed in Figure 6. We initially observe significant differences between 6MFulv (panel a) and DMF (panel b). Overall, we observe a generalized shift to smaller energies for DMF compared to 6MFulv, with DMF showing a very similar profile to that of fulvene (see Figure 2a). 6MFulv, on the other hand, features significantly lower energy differences (of ∼0.3 eV, being almost degenerate for most torsion angles) for large C_1_–C_6_ distances of ∼1.7 Å: this is observed even at 0–20°/160–180° torsions, which suggests single methylation may enable decay through more planar structures, while DMF behaves similarly to fulvene, single methylation breaking the symmetry of the system and thus altering more significantly its behavior.

**Figure 6:**
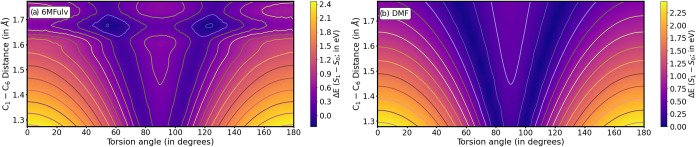
Potential energy cuts for the rigid scan along the torsion (*X*-axis; in degrees) and C_1_–C_6_ stretching (*Y*-axis; in Å) for (a) 6MFulv and (b) DMF at the CASPT2 level of theory, starting from their respective (*S*_1_)_min_ optimized structure (further details are provided in the Section 2). The *Z*-axis represents a color map showing the *S*_1_–*S*_0_ energy difference (in eV), with the same color range applied to both panels for comparison.

Despite their apparent differences in energy across the C_1_–C_6_ and torsion coordinates shown in Figure 6, the addition of methyl groups to the twisting CH_2_ moiety hardly changes the nature of the conical intersection seam. Figure 7a shows the CASPT2 intersection seam along the torsion coordinate, comparing nonmethylated (i.e., fulvene) and methylated species, where small changes can be observed: methylation increases the energy positioning of (*S*_1_/*S*_0_)_CI_^0°^, the effect being additive and thus being largest for DMF, while the rest of the profile is analogous to the parent fulvene molecule but being equally displaced to lower energies for both 6MFulv and DMF due to the lower adiabatic energies of their (*S*_1_)_min_ structures (see Table [Table tblII]) and thus remaining relatively unaffected by substitution. Figure 7b shows analogous topologies across species, with DMF displaying a slightly narrower range of peaked intersections (80–100°); the reported values are, however, borderline ( P≈1 ) and change when other computational parameters are modified, such as the state average (see the SI, Section S3), making the seam topologies across species effectively equivalent.

**Figure 7:**
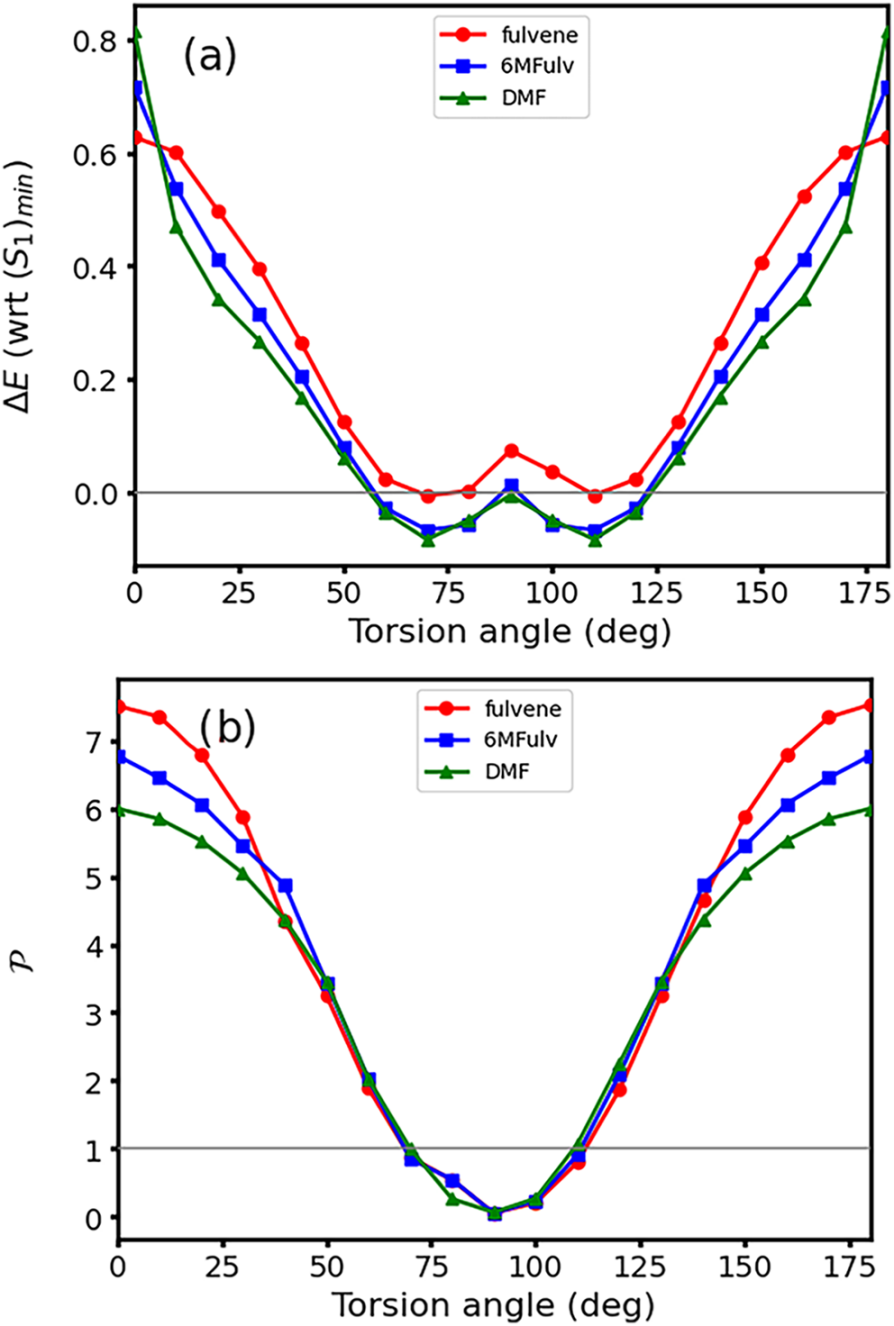
Constrained optimization of the *S*_1_/*S*_0_ conical intersection seam of fulvene (red), 6MFulv (blue), and DMF (green) along the CH_2_ torsion computed at the CASPT2 level of theory. (a) Adiabatic energy (in eV, with respect to (*S*_1_)_min_) along the conical intersection seam. The horizontal gray line showcases Δ*E* = 0. (b) Conical intersection parameter P . The horizontal gray line showcases P=1 , which is used to classify sloped ( P>1 ) vs peaked ( P<1 ) intersections.^53^

To estimate the potential role of methylation along the intersection seam beyond the purely electronic contributions considered, we also looked at the seam in terms of mass weighted differences with respect to the (*S*_1_)_min_ structure (Figure S12): as can be seen, the seam stretches over significantly longer displacements for methylated species, both 6MFulv and DMF being analogous, which reflect the more significant motions required to move them compared to the parent fulvene. This might have implications for the different nuclear motions triggered along the excited state dynamics; the specifics are, however, beyond the capabilities of the present model and the scope of the present work and will be explored in the near future.

We look next at the structural changes along the conical intersection seam: Figure 8 shows changes in their BLA coordinates along the CH_2_ torsion conical intersection seam, which display analogous trends for all systems. Methylation appears to reduce BLA at small torsion values (0–30 and 150–180°), while around 90°, the different species equalize all C–C bond lengths, leading to a BLA value of almost 0. We also observe the spurious emergence of out-of-plane distortions in the cyclopentadienyl ring along the conical intersection seam due to methylation at small torsion values, which do not significantly impact their topology or accessibility, and these are reported in the SI Section S5 for completeness.

**Figure 8:**
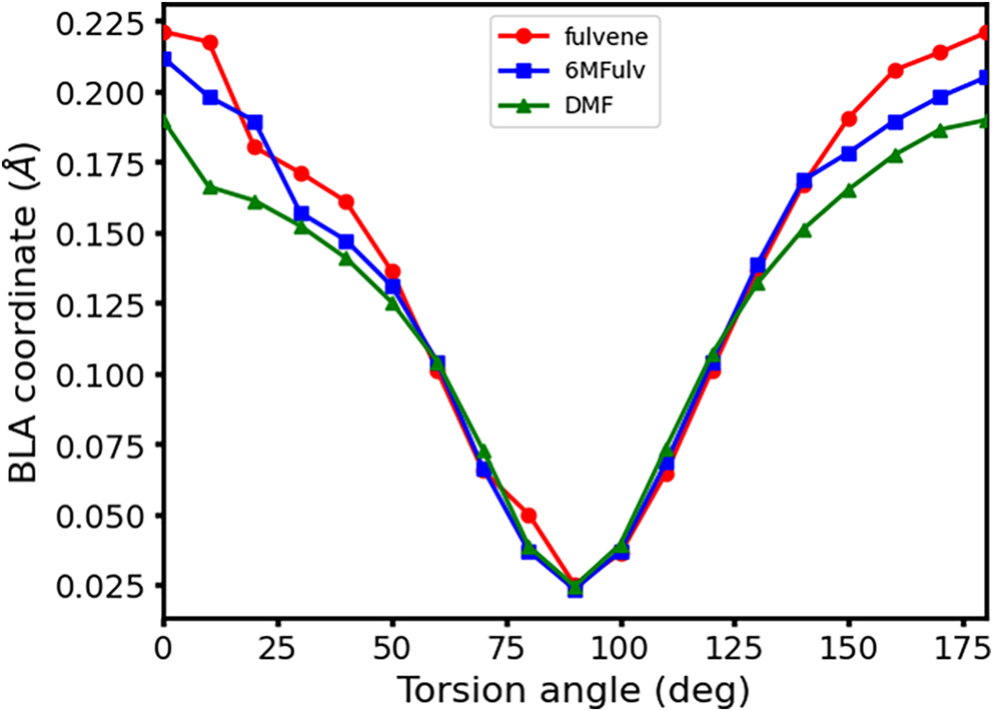
Bond length alternation coordinate changes (in Å) along the CH_2_-like torsion conical intersection seam for fulvene (red), 6MFulv (blue), and DMF (green) at the CASPT2 level of theory.

As in fulvene, we are interested not only in the intersection seam but rather in how it may be accessed. Figure 9 shows the estimated potential energy barriers to reach the different points of the conical intersection seam with varying torsion angles. They display significant increases upon methylation, particularly upon double methylation: DMF shows larger energy barriers across all torsions considered, while 6MFulv estimates are much closer to those of fulvene and show even lower barriers for highly twisted structures (70–90° torsions). To better see these differences, we look at them relative to their respective values in fulvene, which are reported as dots to be confronted against the right-hand *Y*-axis in Figure 9. This shows both methylations significantly increase the energy required to reach partly twisted intersections (30–60°), with the largest difference being predicted at 50°, where ∼3- and ∼6-fold increases are observed for 6MFulv and DMF, respectively, and where the smallest differences are observed at almost planar (0–10°) or completely twisted (80–90°) torsions.

**Figure 9:**
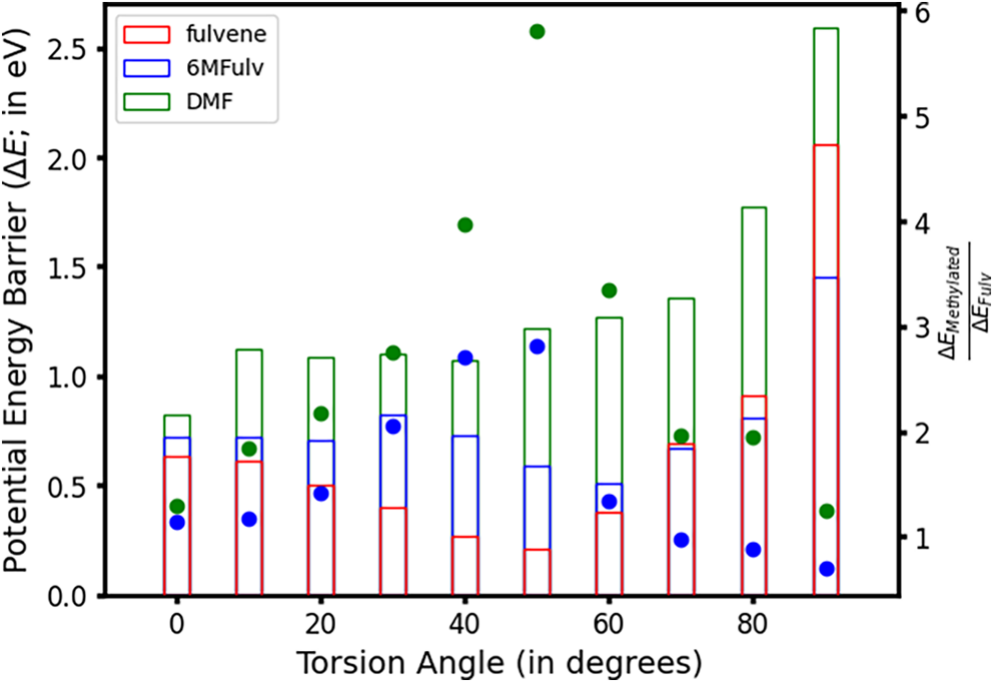
Potential energy barriers (Δ*E*; in eV, represented by bars), computed as the energy difference between the highest-energy point along the linear interpolation in internal coordinates (LIIC) connecting (*S*_1_)_min_ and the different conical intersections and the (*S*_1_)_min_ energy, characterized along the CH_2_ torsion at the CASPT2 level of theory. The right-hand side *Y*-axis compares the Δ*E* values obtained for the methylated species by dividing them by the estimates previously obtained for fulvene (Figure 5 panel b) and are represented with blue dots for 6MFulv and green for DMF.

Overall, we predict the potential energy surfaces of fulvene along the photochemically relevant C_1_–C_6_ stretch and CH_2_ torsion coordinates to be impacted upon single methylation (i.e., 6MFulv) by reducing the *S*_1_–*S*_0_ energy gap at planar and nearly planar structures, while they remain qualitatively unchanged for DMF, even if small quantitative differences are still observed. The conical intersection seam mediating the *S*_1_ → *S*_0_ ultrafast decay upon light absorption is, however, unaffected by methylation, in relative energy, conical intersection topology, and structural terms. We thus suggest DMF to be the more appropriate surrogate methylated species to experimentally record the ultrafast excited state decay of fulvene.

## IV Conclusions

We model the effects of dynamic electron correlation and additionally methylation along the *S*_1_ excited state potential energy surface and conical intersection seam of fulvene by comparing CASSCF results against dynamically correlated CASPT2 approaches and by analyzing how 6-methyl- and 6,6,-dimethyl-fulvene compare against the parent fulvene molecule.

We show how dynamic electron correlation is essential to appropriately model the absorption maximum of fulvene (4.10 eV at CASSCF vs 3.25 eV at CASPT2) and find a quantitative agreement with the 3.34 eV registered experimentally.^31^ We observe decreasing energy differences between CASSCF and CASPT2 along the *S*_1_ excited state decay pathway: dynamic electron correlation induces a 0.2 eV decrease in energy (compared to the CASSCF estimate) at the excited state minimum and a 0.05 eV decrease at the twisted MECI, while displaying a 0.16 eV increase in energy for the planar conical intersection structure.

Structurally, very small differences are observed across methods, both finding a bond length alternation upon relaxing along the *S*_1_ excited state to its minimum, and where we observe a more pronounced (by ∼5°) H_7‑_C_6‑_C_1_–C_5_ torsion for the CASPT2MECI.

Our results show CASPT2 more than doubles the potential energy barrier on *S*_1_ required to access the planar region of the *S*_1_/*S*_0_ crossing seam, thus hampering decay along the C_1_–C_6_ stretching decay channel. Upon exploring the C_1_–C_6_ stretching and CH_2_ reaction coordinates surrounding the (*S*_1_)_min_ structure, we find dynamic electron correlation to increase the *S*_1_–*S*_0_ energy gaps and to decrease the norm of the molecular gradients and nonadiabatic couplings compared to CASSCF, which we expect will lead to slower excited state decays.

Beyond the planar region, both CASSCF and CASPT2 predict an analogous *S*_1_/*S*_0_ conical intersection seam, where we find very similar relative energies and almost identical structures, and where a bond-length alternation motion ensues along the CH_2_ torsion angle until reaching 90° twisting, where all bond lengths effectively equalize.

Following methylation, our CASPT2 estimates qualitatively reproduce the absorption maxima recorded experimentally for 6-methyl-fulvene (6MFulv) and 6,6-dimethyl-fulvene (DMF), which, upon comparison with the parent molecule, show methylation to have a very small effect in the absorption spectrum.

We find singly and doubly methylated species lead to analogous conical intersection seams and other critical structures such as ground and excited state minima but predict 6MFulv to display smaller energy gaps along the C_1_–C_6_ stretching coordinate, thus enhancing decay through this channel compared to fulvene. We thus consider DMF to be the most appropriate fulvene surrogate to record its excited state decay and provide a solid reference to test theoretical predictions against future experiments.

Our work highlights the importance of dynamic electron correlation in the description of the *S*_1_ excited state potential energy landscape of fulvene and methylated derivatives, showing subtle differences that are nevertheless expected to significantly alter their excited state dynamics. Analysis based on nonadiabatic dynamics simulations is underway to thoroughly assess precisely if/how the differences highlighted here due to including dynamic electron correlation in the descriptions of the potential energy surfaces and conical intersection seam affect decay.

## Supplementary Material



## Notes

Computational details such as active space orbitals; a thorough benchmark against other multistate CASPT2 formulations; an analysis of the role of the *S*_2_ electronic state along the *S*_1_/*S*_0_ conical intersection seam; changes in molecular charge along the intersection seam; and the out-of-plane and mass weighted motions along the CH_2_ torsion; all Cartesian coordinates for the critical structures characterized are also supplied (PDF)
