# Supplementary material for: Investigating the Effects of Electron Correlation and Methylation on the S 1/S 0 Conical Intersection Seam of Fulvene
Source: J Chem Theory Comput. 2025 Dec 27;22(1):477–87. doi: 10.1021/acs.jctc.5c01276 (PMC12805561; doi:10.1021/acs.jctc.5c01276)
Supplement: Supplementary file 1 [file ct5c01276_si_001.pdf]

Supporting information for:
Investigating the effects of electron correlation and methylation on the S_1/S_0 conical intersection seam of fulvene

Javier Segarra-Martí
Instituto de Ciencia Molecular, Universitat de València,
C/ Catedrático José Beltrán 2, 46980 Paterna, Valencia, Spain
E-mail: javier.segarra@uv.es

Michael J. Bearpark
Department of Chemistry, Molecular Sciences Research Hub, Imperial College London,
White City Campus, 82 Wood Lane, W12 0BZ, London, UK
E-mail: m.bearpark@imperial.ac.uk

December 14, 2025

Contents

1 CASSCF orbitals S2

2 CASPT2 zeroth-order Hamiltonian: MS-, XDW- and RMS-CASPT2 S2
- 2.1 Critical points along the potential energy surface . . . S2
- 2.2 Decay along the stretching coordinate . . . S2
- 2.3 Torsion and the S_1/S_0 intersection seam . . . S3

3 The role of the S_2 electronic excited state along the S_1/S_0 intersection seam S6

4 Changes in molecular charge along the seam: CASSCF vs CASPT2 S7

5 Out-of-plane and mass-weighted motions motions along the CH_2 torsion intersection seam S8

6 Optimised Cartesian coordinates S10
- 6.1 Fulvene . . . S10
  - 6.1.1 CASSCF . . . S10
  - 6.1.2 XMS-CASPT2 . . . S14
  - 6.1.3 MS-CASPT2 . . . S19
  - 6.1.4 RMS-CASPT2 . . . S24
  - 6.1.5 XDW-CASPT2 . . . S29
- 6.2 6-methyl-Fulvene . . . S33
  - 6.2.1 CASSCF . . . S33
  - 6.2.2 XMS-CASPT2 . . . S39
  - 6.2.3 MS-CASPT2 . . . S45
  - 6.2.4 RMS-CASPT2 . . . S51
  - 6.2.5 XDW-CASPT2 . . . S57
- 6.3 6,6-dimethyl-Fulvene . . . S62
  - 6.3.1 CASSCF . . . S62
  - 6.3.2 XMS-CASPT2 . . . S69
  - 6.3.3 MS-CASPT2 . . . S76
  - 6.3.4 RMS-CASPT2 . . . S83
  - 6.3.5 XDW-CASPT2 . . . S89

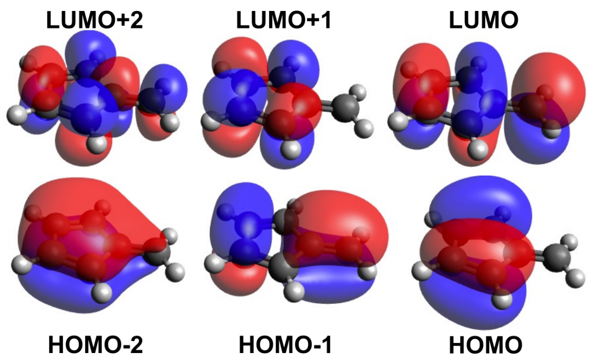

Figure S1: Molecular orbitals included in the active space.

1 CASSCF orbitals

Figure S1 displays the specific orbitals employed in the active space throughout this work.

2 CASPT2 zeroth-order Hamiltonian: MS-, XDW- and RMS-CASPT2

In the main text we provide estimates only for XMS-CASPT2, as it is well-known to address some of the issues presented by other CASPT2 zeroth-order Hamiltonians at and nearby conical intersections.[1] Here we show the values obtained for multistate (MS-),[2] extended dynamically weighted (XDW-)[3] and rotated multistate (RMS)-CASPT2:[4] as it will be seen, there are very subtle (i.e. negligible) changes due to the specific *multistate* CASPT2 Hamiltonian used, which is in line with what has been recently observed by Ibele *et al.* using *ab initio* multiple spawning non-adiabatic molecular dynamics.[5]

2.1 Critical points along the potential energy surface

Table S1: MS- (in brackets), XDW- (in squared brackets) and RMS-CASPT2 adiabatic energies (ΔE, in eV) of fulvene at the different critical structures characterised in this work: $(S_0)_{min}$, $(S_1)_{min}$, $(S_1/S_0)_{MECI}$ and $(S_1/S_0)^{0°}_{CI}$.

State	$(S_0)_{min}$	$(S_1)_{min}$	$(S_1/S_0)_{MECI}$	$(S_1/S_0)^{0°}_{CI}$
S_0	0.00 (0.00) [0.00]	0.91 (0.91) [0.91]	2.29 (2.29) [2.29]	2.91 (2.91) [2.91]
S_1	3.20 (3.20) [3.20]	2.32 (2.32) [2.32]	2.29 (2.29) [2.29]	2.91 (2.91) [2.91]

Table S1 shows the relative energies for the different critical points characterised at the RMS-, XDW- and MS-CASPT2 levels of theory. As can be seen, both provide analogous results, diverging only in the meV range (not shown as such differences are not relevant due to being well below the expected accuracy of the methods). We observe the values are all red-shifted with respect to those reported in Table I in the main text for XMS-CASPT2: this is expected and well-known, as the state-averaged Fock operator used in XMS-CASPT2[6, 7] tends to overestimate the resulting vertical excitation energies.[8, 9] This difference is smallest at the equilibrium FC region or $(S_0)_{min}$, and increases along the S_1 excited state decay as we move onto $(S_1)_{min}$, $(S_1/S_0)_{MECI}$ and $(S_1/S_0)^{0°}_{CI}$, resulting in blue-shifts of 0.05, 0.06, 0.09 and 0.10, respectively. All these values are in any case below the expected accuracy of the method, which is placed at ~0.2-0.3 eV.[10]

RMS-, XDW- and MS-CASPT2 predict $(S_1/S_0)_{MECI}$ to be energetically below $(S_1)_{min}$ even if only by 0.03 eV, which is in qualitative agreement with the reported XMS-CASPT2 results in the main text that place both structures at the same adiabatic energy. $(S_1/S_0)^{0°}_{CI}$, on the other hand, shows a more significant 0.1 eV energy difference, which is relatively small with the overall energy barrier estimated at XMS-CASPT2 in the main text (0.63 eV).

The negligible differences in energy are also in line with the analogous structures obtained with RMS-, XDW- and MS-CASPT2 Hamiltonians (i.e. differences in the sub-pm range for distances), as shown in Table S2, and which are also in line with those reported at the XMS-CASPT2 level in Table 1 of the main text.

2.2 Decay along the stretching coordinate

To verify the negligible role of the zeroth-order hamiltonian along decay, Table S2 shows the the main geometrical parameters of all critical points characterised. These are in line with the values shown in Fig. 1 of the main

Table S2: Main structural parameters for the RMS-CASPT2 (MS-CASPT2 in brackets and XDW-CASPT2 in squared brackets) optimised structures of $(S_0)_{min}$, $(S_1)_{min}$, $(S_1/S_0)_{MECI}$ and $(S_1/S_0)^{0°}_{CI}$ in fulvene. Bond length distances are reported in Å, while torsion and angles are in degrees. $C_1{-}C_2$ and $C_2{-}C_3$ distances are not shown due to being equivalent to $C_1{-}C_5$ and $C_4{-}C_5$, respectively.

	$C_1{-}C_6$	$C_1{-}C_5$	$C_4{-}C_5$	$C_3{-}C_4$	$C_2{-}C_1{-}C_6{-}H_7$
$(S_0)_{min}$	1.364 (1.364)	1.477 (1.477)	1.376 (1.376)	1.484 (1.484)	-0.001 (-0.001)
$(S_1)_{min}$	1.474 (1.474)	1.413 (1.413)	1.472 (1.472)	1.384 (1.384)	-0.007 (-0.007)
$(S_1/S_0)_{MECI}$	1.475 (1.475)	1.418 (1.418)	1.475 (1.475)	1.383 (1.383)	-68.460 (-68.452)
$(S_1/S_0)^{0°}_{CI}$	1.600 (1.599)	1.371 (1.370)	1.560 (1.560)	1.324 (1.324)	0.000 (0.000)

text obtained with XMS-CASPT2, showing negligible deviations, and being close to the CASSCF values also reported in the main text.

2.3 Torsion and the S_1/S_0 intersection seam

Upon excitation, bond length alternation motions are triggered in fulvene leading to its excited state minimum $(S_1)_{min}$ structure, which shows an elongation of the $C_1{-}C_6$ and a shortening of the $C_3{-}C_4$ bonds. After this initial structural reorganisation, the system is then ready to twist along the $C_2{-}C_1{-}C_6{-}H_7$ torsion angle. To analyse the potential energy surfaces after reaching $(S_1)_{min}$, we have computed a rigid scan along the $C_1{-}C_6$ distance and the $C_2{-}C_1{-}C_6{-}H_7$ torsion angle, and which is shown in Fig. S2.

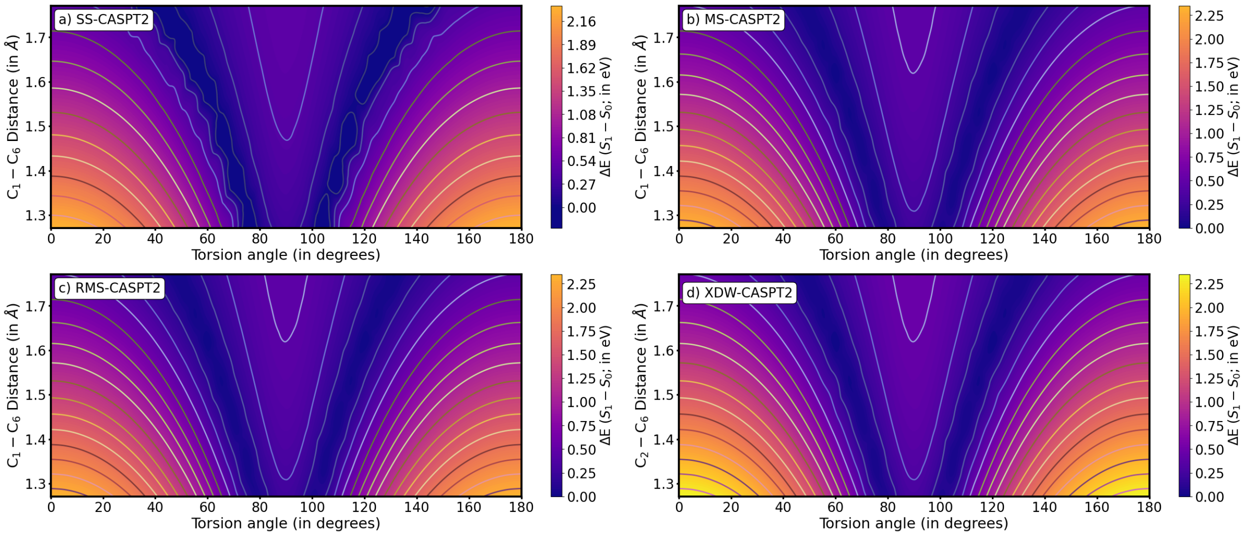

Figure S2: Potential energy rigid scans along the $C_1{-}C_6$ and $C_2{-}C_1{-}C_6{-}H_7$ torsion angles from the $(S_1)_{min}$ structure, with different levels of theory: a) single-state (SS)-CASPT2, b) multistate (MS)-CASPT2, c) rotated multistate (RMS)-CASPT2, and d) extended dynamically weighted (XDW)-CASPT2. Energy differences between S_1 and S_0 states are provided as a contour plot, with dark purple values denoting energy degeneracies (i.e. $\Delta E \approx 0$).

As can be seen from Fig. S2, all methods explored herein (and also including those reported in Fig. 2 of the main text) lead to qualitatively analogous descriptions of the potential energy surface around $(S_1)_{min}$, with SS-CASPT2 being the outlier as it lacks a multistate treatment and is slightly red-shifted while showing small negative values along the surface due to changes in state ordering.

The main text mentions the values of $\mathcal{B}$ associated to the different points characterised along the conical intersection seam; we include here values for Fulvene (red), 6-methyl-fulvene (6MFulv; in blue) and 6,6-dimethyl-fulvene (DMF; in green) in Fig. S3. As can be seen, $\mathcal{B} > 1$ across the torsion angle with the exception of the 90° point, which is classed as bifurcating. This is reproduced by all methylated systems, as shown in Fig. S3a), and further supports the small role of methylation in the energetics and topography of the conical intersection seam discussed in more detail in the main text.

The specific effects of the multistate treatment on the topology along the conical intersection seam are considered for fulvene (Fig. S3b)): as can be seen, MS- and RMS-CASPT2 lead to analogous results for $\mathcal{P}$, which are in turn equivalent to those obtained for XMS-CASPT2 and reported in the main text, whereas XDW-CASPT2 appears to deviate more significantly, placing $\mathcal{P} > 1$ for the 80−100° range. Comparing the $\mathcal{B}$ values (i.e. dashed lines in Fig. S3b)) with those reported for XMS-CASPT2 in Fig. S3a), we see how

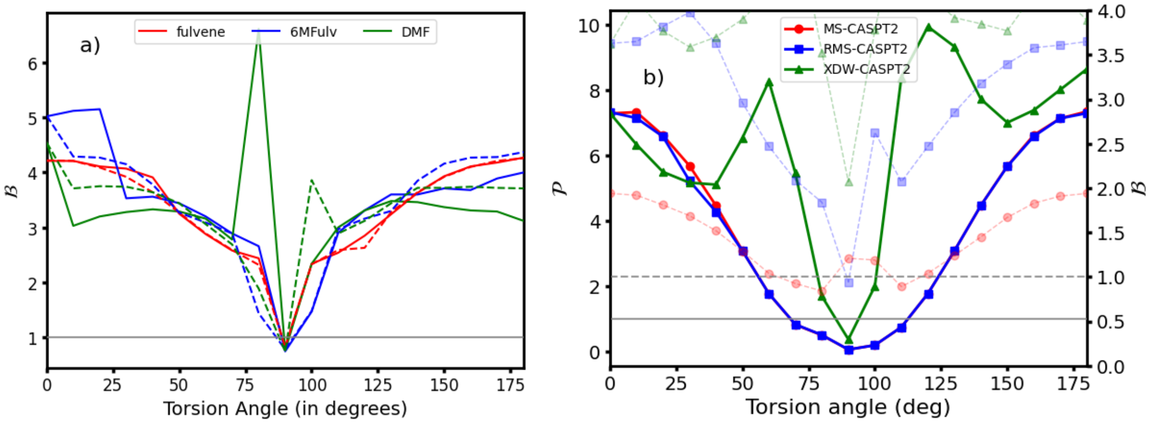

Figure S3: a) $\mathcal{B}$ values of the different points characterised along the $C{=}CH_2$ torsion angle in fulvene (red), 6MFulv (blue) and DMF (green). Values above 1 (denoted by a grey line) refer to single path topologies, whereas values under 1 refer to bifurcating. Full thick lines refer to unconstrained optimisations (beyond the constraint on the dihedral angle), whereas dashed lines denote estimates while enforcing ring planarity (see Computational Details in the main text). b) Changes in $\mathcal{P}$ and $\mathcal{B}$ parameters for fulvene due to the different multistate CASPT2 formulations: MS-CASPT2 (red), RMS-CASPT2 (blue) and XDW-CASPT2 (green), XMS-CASPT2 values having been already provided in panel a). Full thick lines refer to $\mathcal{P}$ values, whereas dashed lines denote $\mathcal{B}$ estimates, the grey lines showcasing the threshold value of 1 for both cases.

RMS-CASPT2 is the only one that reproduces the single bifurcating point at 90°: MS-CASPT2 shows several values as bifurcating, displaying the actual 90° point as single path, whereas XDW-CASPT2 does not feature a single point as bifurcating.

With this we can thus see how XMS-CASPT2 and RMS-CASPT2 behave very similarly and would therefore be the optimal choices to characterise the conical intersection seam of fulvene; MS-CASPT2 displays the known shortcomings close to degeneracies, whereas the interpolation scheme in XDW-CASPT2 appears to not retain the correct behaviour throughout the seam.

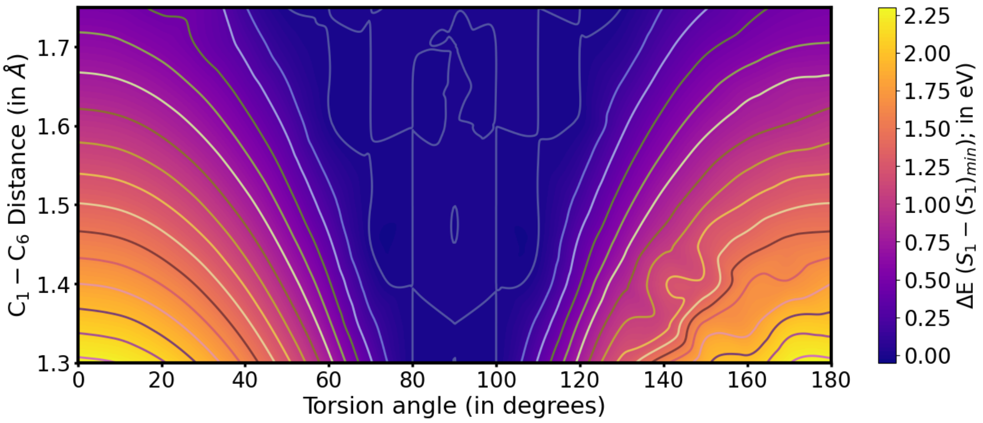

Figure S4: Potential energy cuts for a S_1 relaxed scan along the torsion (X-axis; in degrees) and $C_1{-}C_6$ stretching (Y-axis; in Å) at the XMS-CASPT2 level of theory in fulvene. The Z-axis represents a colour map showing the $S_1 - S_0$ energy difference (in eV).

Another aspect to consider is the potential role of relaxation in the potential energy profiles showcased along the $C_1{-}C_6$ and CH_2 torsion degrees of freedom. Fig. S4 shows the $S_1 - S_0$ energy difference resulting from a relaxed scan along the S_1 excited state at the XMS-CASPT2 level of theory. As can be seen, the results are qualitatively analogous to those represented by rigid scans int he main text (Fig. 2a) and validate the approach used. Using rigid scans allows for comparing CASSCF and XMS-CASPT2 on an even footing (i.e. on the same geometries), which is part of the reason why the rigid scans were left in the main text.

The differences in the zeroth-order CASPT2 Hamiltionians revolve around the usage of either a state-specific or state-averaged Fock operator (or an interpolation between them), and whether the effective Hamiltonian is pre-diagonalised and how, prior to the subsequent perturbation treatment.[11] These are therefore, to some degree, encoded in their resulting off-diagonal coupling elements, which we depict in Fig. S5: here we can

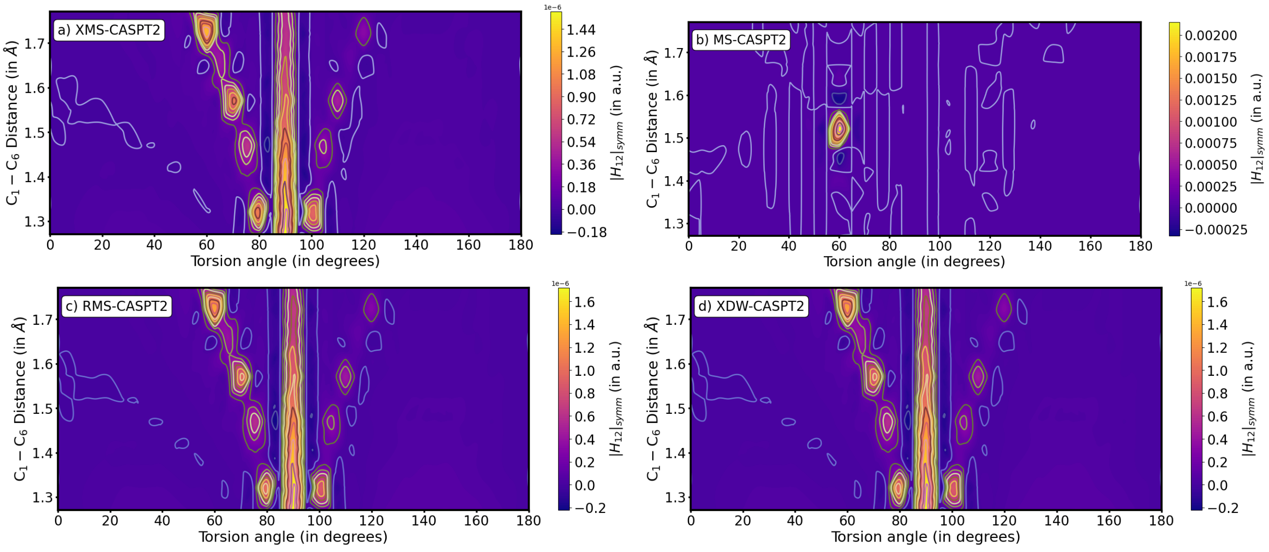

Figure S5: Module of the symmetric off-diagonal elements ($|H_{12}|_{symm}$, in a.u.) of the multistate CASPT2 Hamiltonian for a) XMS-, b) MS-, c) RMS-, and d) XDW-CASPT2 formulations along the C_1-C_6 stretch and torsion coordinates of fulvene.

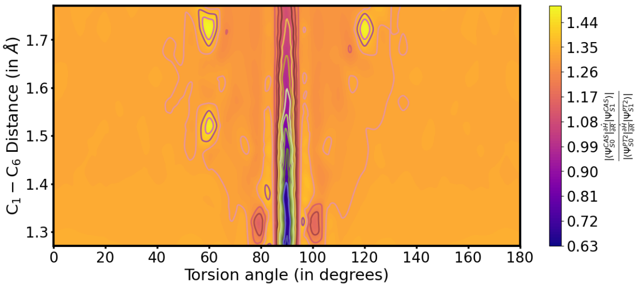

Figure S6: Quotient of the Frobenius norm of the non-adiabatic coupling vector between the CASSCF and CASPT2 levels of theory along the torsion (X-axis; in degrees) and C_1-C_6 stretching (Y-axis; in Å) in fulvene starting from the $(S_1)_{min}$ optimised structure.

see MS-CASPT2 presents a spike in the off-diagonal element at ∼1.5 Å C_1-C_6 bond length distance and 60° torsion, which could potentially lead to the well-documented discontinuities in energy reported at and around conical intersections. The values obtained for MS-CASPT2, outside the outlier previously mentioned of ∼0.002 a.u. (∼0.05 eV; i.e. still very small), are of the same order as those for the other formulations, which points at very similar potential energy surfaces. The other three methods (XMS-, RMS- and XDW-CASPT2) behave almost identically, showing non-zero coupling values only along the intersection seam (i.e. when both states are strongly coupled), and even then the values are very small ($\sim10^{-6}$ a.u.), which results in the analogous energies observed above in Fig. S2 and Fig. 2a) of the main text (for XMS-CASPT2).

To further assess the likelihood of non-adiabatic transitions it is convenient to account for the way in which the states may be coupled. Fig. S6 shows the quotient of the CASSCF over the CASPT2 Frobenius norm of the $S_0 - S_1$ non-adiabatic coupling vector, which is often used in non-adiabatic molecular dynamics to both estimate the probability of transferring population across electronic states[12, 13] and also to redistribute the excess energy after such transfers;[14, 15] we here assume larger norms will enhance population transfer, even if the norm itself is not directly (nor solely) involved in computing population transfer probabilities and also depends on the velocities.[12] We have used here the non-adiabatic coupling vector instead of employing the total derivative coupling,[16] which is often used in semi-classical formulations for transition probabilities, as the latter is divided by the energy difference and is thus prone to discontinuities when encountering energy degeneracies. As can be seen, CASSCF norms are ∼30% larger (i.e. values of ∼1.3) across the whole space besides the ∼90° torsion region where this trend is inverted and that approaches equal values at long (>1.6 Å) C_2-C_6 distances. This larger norm also applies to nuclear gradients (Fig. S7): CASSCF is known to overestimate nuclear gradients and the resulting bond length distances during optimisations compared to the dynamically electron correlated CASPT2,[17, 18] and here we show this also extends to their associated non-adiabatic couplings.

In more depth, Fig. S7 compares the Frobenius norm of the S_0 and S_1 gradients at CASSCF and XMS-CASPT2 levels of theory along the C_1-C_6 stretch and $C_2-C_1-C_6-H_7$ torsion angle, where we observe a ∼1.35

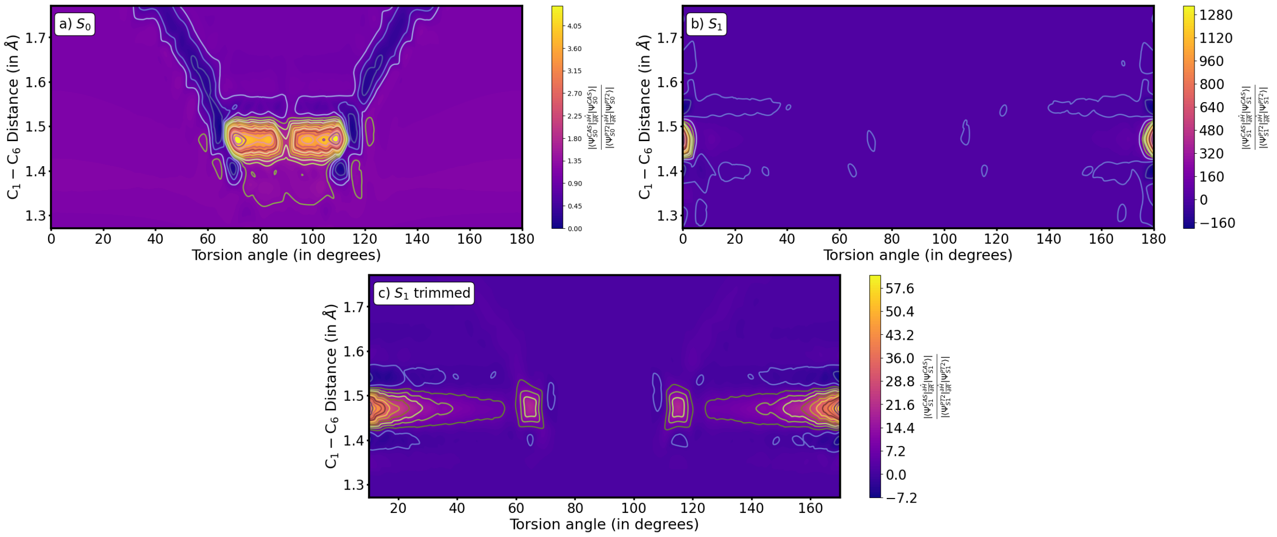

Figure S7: Quotient of the Frobenius norm of the a) S_0 and b) S_1 gradients between the CASSCF and XMS-CASPT2 levels of theory along the torsion (X-axis; in degrees) and C_1-C_6 stretching (Y-axis; in Å) in fulvene. Panel c) depicts the quotient of the norm of S_1 for a trimmed area, to better discern changes away from small torsion angle values.

times increase of the norm across most of the explored space (similar to what was observed for the non-adiabatic couplings in S6), while showing decreases in the S_0 molecular gradient at the vicinities of the intersection seam (i.e. the XMS-CASPT2 gradient appears to be larger there), and with a sudden increase at distances of ∼1.5 Å and torsion angles 70-110°.

The S_1 gradient, on the other hand, presents much larger differences: at 0° torsion and ∼1.5 Å C_1-C_6 distance we observe a three orders of magnitude difference between CASSCF and XMS-CASPT2 estimates, which is however overestimated due to using the XMS-CASPT2 $(S_1)_{min}$ structure as a reference, and this region thus referring XMS-CASPT2 gradients at a minimum which are very close to 0. Fig. S7c) shows the quotient of the S_1 gradient excluding the values at 0-10° and 170-180° torsions that obscure the remaining conformational space, and where we still observe significant differences (almost up to ×60) at ∼1.5 Å distances and almost all torsion angles, excluding the 80-100° range. Despite being partly due to the way the geometries are sampled as described above, these large changes in the norm of the S_1 absorbing state are nevertheless likely to induce changes in the ensuing excited state dynamics, as they are also observed away from $(S_1)_{min}$ and at small torsion values that may be accessible upon sampling the ground state for preparation of the initial wave packet.

3 The role of the S_2 electronic excited state along the S_1/S_0 intersection seam

The simulations presented in the main text only considered the ground and first electronic excited states, namely S_0 and S_1. Upon twisting (particularly ∼90° torsion), however, an ionic (S_2)[19, 20] electronic excited state stabilises in energy, which may potentially interfere in the description of the S_1/S_0 conical intersection seam.

Figure S8 shows the intersection seam from Figure 6 in the main text, accounting in this case for the lowest-energy 5 electronic states, and thus ensuring the inclusion of the S_2 ionic state (shown in blue). CASSCF estimates are omitted here as by not including dynamic electron correlation, the S_2 at this level of theory appears heavily blue-shifted and a potential crossing with the S_1/S_0 intersection seam is not a concern as it has been shown elsewhere.[19]

Our XMS-CASPT2 optimisations averaged over 5 states show the intersection seam remains relatively unaffected. Even at 90° torsion, which stabilises S_2 the most, the S_2 electronic state still lies ∼1.5 eV above the S_1/S_0 degeneracy. This is in line with previous studies which were unable to locate a S_2/S_0 crossing.[20]

Interestingly, conical intersection topographies along the intersection seam are slightly affected: the range in which peaked intersections appear reduces to 80−100° torsion, i.e. upon including more interacting states decreases the torsion range showing a peaked character. This correlates with the increasingly sizeable contributions of the H-1→L configuration at such twisted structures and can already be seen in Figure 6 in the main text for the calculations averaging over 2 states, and is also reflected in the way the $S_2 - S_1/S_0$ energy gap correlates with $\mathcal{P}$ decrease in Figure S8.

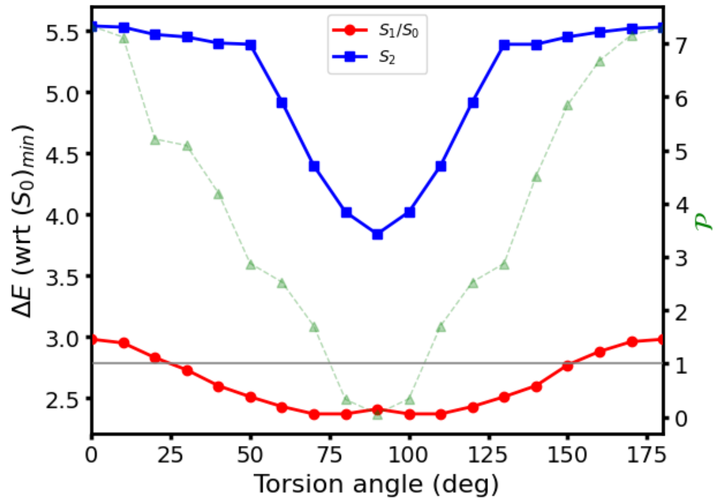

Figure S8: XMS-CASPT2 constrained optimisation along the CH_2 torsion angle, depicted as an in-set on the bottom-left corner, of the S_1/S_0 conical intersection (red) and the S_2 state (blue) of fulvene. The left-hand side y-axis denotes the adiabatic energy (in eV) of the seam and corresponds to the full lines, while the right-hand side (green) depicts the parameter $\mathcal{P}$ that helps characterise S_1/S_0 conical intersection topology, and whose values are provided on dashed transparent green lines. The horizontal grey line showcases $\mathcal{P} = 1$ which is used to classify sloped ($\mathcal{P} > 1$) *vs* peaked ($\mathcal{P} < 1$) conical intersections.[16]

We can therefore confirm S_2 does not directly interact with the S_1/S_0 seam, but its stabilisation at $\sim$90° torsion angles leads to a S_0 wave function of H-1→L character, which is in turn responsible for the conical intersection topography changes (i.e. going from sloped to peaked) thoroughly discussed in the main text.

4 Changes in molecular charge along the seam: CASSCF vs CASPT2

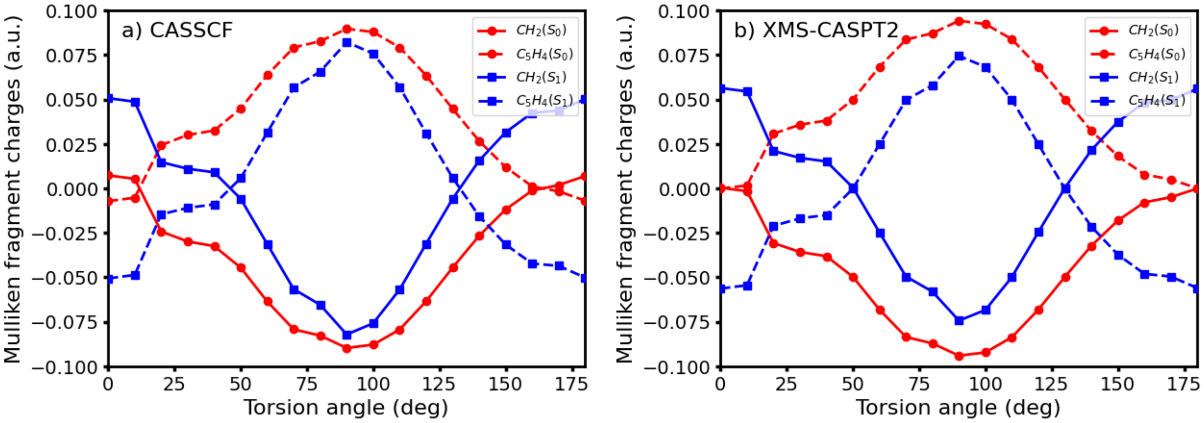

Figure S9: Partial Mulliken charges for the S_0 (in red) and S_1 (in blue) along the CH_2 twisting intersection seam for a) CASSCF and b) XMS-CASPT2 levels of theory. Full lines refer to the CH_2 moiety whereas dashed lines denote the cyclopentadiene C_5H_4 ring.

Figure S9 shows the changes in partial Muliken charges along the CH_2 twisting intersection seam for both CASSCF and XMS-CASPT2 levels of theory. As can be seen, both can be considered to be qualitatively equivalent, charge separation therefore being accounted for in both CASSCF and XMS-CASPT2 treatments of electron correlation. The most noticeable feature is the charge inversion along the CH_2 twisting coordinate for the S_1 state (blue lines), which is recovered at both levels of theory from $\sim$50° − 120° torsion.

5 Out-of-plane and mass-weighted motions motions along the CH_2 torsion intersection seam

In the main text we have covered the conical intersection seam in fulvene, 6MFulv and DMF along the CH_2 torsion (Figs. 3 and 7 in the main text) while including constraints in the optimisation procedure to ensure the cyclopentadiene moiety does not pucker. This was done for two main reasons: i) we observed the optimised structures mostly retained ring planarity even when not enforcing it, and ii) even in those cases where slightly puckered structures were observed, the energies and conical intersection topographies hardly changed. However, in methylated species and when including dynamic electron correlation (i.e. in CASPT2 optimisations) we have observed slightly more puckered structures, which nevertheless lead to very similar results to those of the planar structures reported in the main text.

To monitor this we here report r_{IJ},[16] which allows comparison between different conical intersections by projecting the branching space of one (x', y') onto the other (x, y):

$$r_{IJ} = |(x \cdot x')(y \cdot y') - (x \cdot y')(y \cdot x')| \tag{1}$$

where r_{IJ} takes values between 0 and 1, with 0 representing an orthogonal and 1 parallel branching planes, respectively.

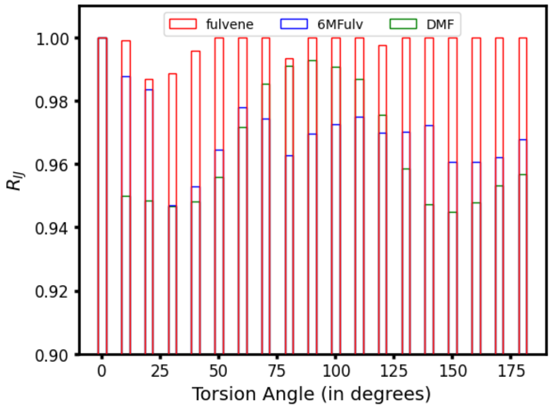

Figure S10: R_{IJ} values comparing the branching space along the CH_2 torsion conical intersection seam when enforcing planarity on the pentadiene ring *vs* the unconstrained optimisation for fulvene (red), 6Mfulv (blue) and DMF (green).

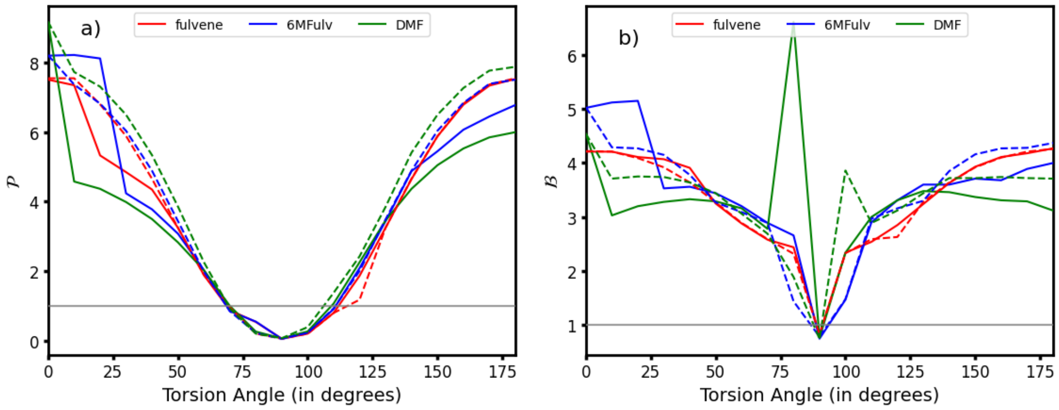

Figure S11: Conical intersection parameters $\mathcal{P}$ (panel a) and $\mathcal{B}$ (panel b) along the conical intersection seam for fulvene (red), 6MFulv (blue) and DMF (green) comparing optimisations when enforcing planarity on the pentadiene ring (dashed lines) *vs* unconstrained (thick lines).

Fig. S10 projects the branching spaces of planar-constrained and unconstrained optimised conical intersections along the CH_2 torsion. As can be seen, despite featuring slight out-of-plane deformations, their branching spaces are almost identical, which is also observed when looking at the $\mathcal{P}$ and $\mathcal{B}$ parameters (Fig. S11): even though quantitative differences might be observed in some cases, qualitatively the results are analogous (i.e. whether they're above or below the threshold of 1). With this we can safely assume that any spurious out-of-plane motion along the CH_2 torsion conical intersection seam is not expected to significantly alter reactivity.

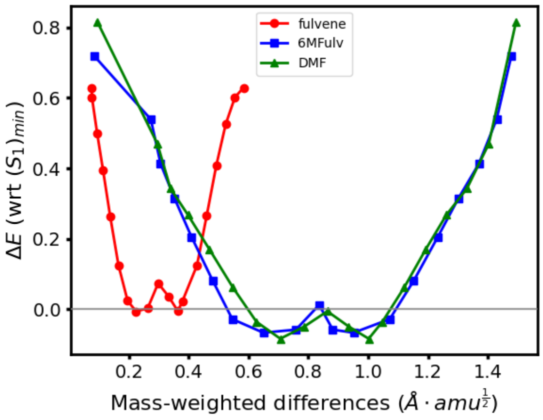

Figure S12: Constrained optimisation of the S_1/S_0 conical intersection seam of fulvene (red), 6MFulv (blue) and DMF (green) along the CH_2 torsion computed at the CASPT2 level of theory and displayed in mass-weighted differences. The x-axis shows the mass-weighted differences computed as the different points of the seam with respect to the $(S_1)_{min}$ structure that is first accessed along the excited state decay. The y-axis depicts the adiabatic energies (in eV, with respect to $(S_1)_{min}$, with the horizontal grey line showcasing $\Delta E = 0$.

We furthermore consider differences introduced upon methylation in terms of the mass, and its effects in the resulting structures along the seam: Fig. S12 showcases the CH_2 conical intersection seam recomputed in terms of mass-weighted differences, where all points of the seam are confronted against the $(S_1)_{min}$ structure used as reference. As can be seen, significant differences arise upon methylation, with the differences becoming much bigger, as expected upon inserting a heavier methyl group, this difference however not being too different for 6MFulv and DMF.

6 Optimised Cartesian coordinates

6.1 Fulvene

6.1.1 CASSCF

$(S_0)_{min}$

C	1.17687730	-0.11463482	0.00010758
C	0.00325987	0.77914721	-0.00001084
C	-1.17793768	-0.10459286	-0.00009377
C	0.73530848	-1.39907464	-0.00000910
C	-0.74733944	-1.39275192	0.00001088
C	0.00902105	2.13021810	0.00000542
H	-0.91338704	2.69428848	0.00042773
H	0.93620586	2.68640192	-0.00044598
H	-2.19892293	0.24142924	-0.00012299
H	2.20077660	0.22266681	0.00015056
H	-1.36618491	-2.27630062	-0.00000640
H	1.34659618	-2.28786901	-0.00001309

$(S_1)_{min}$

C	1.12433619	-0.09191896	-0.00001815
C	0.00310727	0.74339773	0.00001177
C	-1.12520494	-0.08232611	0.00002767
C	0.67389223	-1.49412118	-0.00001181
C	-0.68673614	-1.48831901	0.00000730
C	0.00948855	2.23980423	0.00002730
H	-0.91719032	2.78950285	-0.00019165
H	0.94082190	2.78157955	0.00014814
H	-2.15233826	0.24827644	0.00004865
H	2.15425169	0.22991159	-0.00003445
H	-1.34366114	-2.34274277	0.00001411
H	1.32350633	-2.35411647	-0.00002889

$(S_0/S_1)_{MECI}$

C	1.12468559	-0.09542482	-0.12452857
C	0.00306470	0.74765271	0.00016101
C	-1.12556597	-0.08607561	0.12445462
C	0.67547920	-1.48541658	-0.07655206
C	-0.68799795	-1.47975113	0.07585648
C	0.00924994	2.22768129	0.00047558
H	-0.49406620	2.78347092	-0.77665309
H	0.51727676	2.77891303	0.77778498
H	-2.14470697	0.24644456	0.24427906
H	2.14657142	0.22861774	-0.24420372
H	-1.33574789	-2.33808299	0.14979668
H	1.31603073	-2.34910124	-0.15087100

$(S_0/S_1)_{CI}^{0^\circ}$

C	1.10571523	-0.08436391	-0.00138037
C	0.00303282	0.72945102	-0.00022572
C	-1.10652132	-0.07495839	0.00151962
C	0.65328094	-1.54792670	-0.00043162
C	-0.66659509	-1.54231376	0.00092962
C	0.00977311	2.30539368	-0.00013026
H	-0.92055865	2.84653353	0.00141525
H	0.94471182	2.83853216	-0.00102992
H	-2.13614774	0.25146404	-0.00073791
H	2.13808165	0.23328909	0.00018999
H	-1.34199460	-2.38241562	-0.00167800
H	1.32149518	-2.39375725	0.00155932

$(S_0/S_1)_{CI}^{10^\circ}$

C	1.10626987	-0.08425669	0.01850494
C	0.00317809	0.73038128	0.00095547

C	-1.10689429	-0.07465657	-0.01895814
C	0.65366570	-1.54621533	0.00906840
C	-0.66693042	-1.54060105	-0.00900145
C	0.00972951	2.30233907	0.00088836
H	-0.90904790	2.84394183	0.14620400
H	0.93270543	2.83618507	-0.14650030
H	-2.13610524	0.25200867	-0.04319887
H	2.13830825	0.23346173	0.04145510
H	-1.34177158	-2.38109697	-0.01471765
H	1.32116592	-2.39256318	0.01530013

$(S_0/S_1)^{20^\circ}_{CI}$

C	1.10760321	-0.08424216	0.03750957
C	0.00317072	0.73324629	-0.00102621
C	-1.10822835	-0.07473585	-0.03935527
C	0.65473849	-1.54125338	0.01963109
C	-0.66820967	-1.53560816	-0.01807575
C	0.00969375	2.29435577	0.00019051
H	-0.87400798	2.83703604	0.28935846
H	0.89797481	2.83061052	-0.28685106
H	-2.13653749	0.25202869	-0.08496240
H	2.13873394	0.23367891	0.08187868
H	-1.34137633	-2.37741411	-0.02962990
H	1.32071823	-2.38877469	0.03133229

$(S_0/S_1)^{30^\circ}_{CI}$

C	1.10986193	-0.08437151	0.05672860
C	0.00304209	0.73703323	-0.00092997
C	-1.11075076	-0.07501110	-0.05743970
C	0.65715568	-1.53274971	0.02880858
C	-0.67040100	-1.52709656	-0.02816081
C	0.00960921	2.28112300	-0.00029726
H	-0.81652169	2.82680175	0.42392876
H	0.84087458	2.82041860	-0.42263635
H	-2.13760127	0.25213659	-0.12439828
H	2.13943890	0.23405063	0.12385833
H	-1.34071732	-2.37104561	-0.04804223
H	1.32028299	-2.38236144	0.04858034

$(S_0/S_1)^{40^\circ}_{CI}$

C	1.11333073	-0.08566010	0.07494411
C	0.00296173	0.74122499	-0.00153032
C	-1.11446176	-0.07640946	-0.07607787
C	0.66064254	-1.52071444	0.03997941
C	-0.67383954	-1.51513006	-0.03940568
C	0.00947699	2.26482875	-0.00046267
H	-0.73814486	2.81339510	0.54882341
H	0.76312332	2.80804287	-0.54680592
H	-2.13937165	0.25160423	-0.16155428
H	2.14087064	0.23385507	0.16107559
H	-1.33930659	-2.36250054	-0.07103575
H	1.31899179	-2.37360854	0.07204999

$(S_0/S_1)^{50^\circ}_{CI}$

C	1.11807166	-0.08869386	0.09438213
C	0.00312894	0.74526331	0.00002851
C	-1.11887615	-0.07913079	-0.09447710
C	0.66588325	-1.50631681	0.05309458
C	-0.67887181	-1.50051293	-0.05306568
C	0.00953489	2.24740295	0.00008379
H	-0.64241143	2.79979818	0.65833637
H	0.66614148	2.79420032	-0.65820828
H	-2.14161256	0.25042380	-0.19448785
H	2.14359097	0.23205348	0.19450357
H	-1.33806676	-2.35206380	-0.09957816
H	1.31776088	-2.36349597	0.09938813

$(S_0/S_1)_{CI}^{60^\circ}$

C	1.12342428	-0.09357515	0.11307011
C	0.00314503	0.74752481	0.00002772
C	-1.12429655	-0.08394769	-0.11306952
C	0.67297793	-1.49051365	0.06790626
C	-0.68584782	-1.48471735	-0.06790396
C	0.00948083	2.23177931	0.00005050
H	-0.52992559	2.78681181	0.75274424
H	0.55358559	2.78221480	-0.75263537
H	-2.14477588	0.24778982	-0.22437092
H	2.14671012	0.22939794	0.22439177
H	-1.33695266	-2.34124859	-0.13271333
H	1.31674809	-2.35258820	0.13250251

$(S_0/S_1)_{CI}^{70^\circ}$

C	1.12878373	-0.10005151	0.13131477
C	0.00311452	0.74674102	0.00037826
C	-1.12969839	-0.09034853	-0.13106289
C	0.68169062	-1.47609858	0.08285885
C	-0.69436973	-1.47017089	-0.08314358
C	0.00954205	2.22134864	0.00014379
H	-0.40383963	2.77791224	0.82871817
H	0.42723474	2.77384401	-0.82900071
H	-2.14758533	0.24435350	-0.25367690
H	2.14949524	0.22592507	0.25393860
H	-1.33677613	-2.33155060	-0.16733933
H	1.31668168	-2.34297651	0.16687097

$(S_0/S_1)_{CI}^{80^\circ}$

C	1.13217650	-0.10628977	0.15038751
C	0.00313947	0.74322875	0.00023519
C	-1.13305505	-0.09653269	-0.15059139
C	0.68972277	-1.46623050	0.09588317
C	-0.70231958	-1.46021880	-0.09617002
C	0.00951484	2.21787596	0.00016745
H	-0.26829807	2.77389639	0.88415277
H	0.29153502	2.77137909	-0.88406243
H	-2.14780505	0.24147200	-0.28743645
H	2.14978295	0.22296344	0.28734227
H	-1.33749241	-2.32558730	-0.19313241
H	1.31737200	-2.33702872	0.19322434

$(S_0/S_1)_{CI}^{90^\circ}$

C	1.13137039	-0.10859910	0.17283262
C	0.00306709	0.74068567	-0.00057909
C	-1.13248123	-0.09904037	-0.17327618
C	0.69227955	-1.46262555	0.10726148
C	-0.70494608	-1.45672441	-0.10705318
C	0.00921777	2.21769066	-0.00002676
H	-0.12817425	2.77179428	0.91759001
H	0.15228179	2.77166664	-0.91683996
H	-2.14410682	0.24039653	-0.32796073
H	2.14582704	0.22227172	0.32753932
H	-1.33810528	-2.32368304	-0.20303706
H	1.31804343	-2.33490517	0.20354953

$(S_0/S_1)_{CI}^{100^\circ}$

C	1.13136507	-0.10792725	0.18045096
C	0.00306751	0.73976291	-0.00028333
C	-1.13247497	-0.09833061	-0.18028056
C	0.69179970	-1.46195132	0.10864873
C	-0.70443597	-1.45606920	-0.10878437
C	0.00931262	2.21642778	-0.00017742
H	-0.10873274	2.77020417	0.92043861
H	0.13269578	2.76982254	-0.92031945

H	-2.14474128	0.24110042	-0.33139633
H	2.14643507	0.22289023	0.33186587
H	-1.33789505	-2.32288412	-0.20394946
H	1.31787767	-2.33411770	0.20378676

$(S_0/S_1)_{CI}^{110^\circ}$

C	1.11648942	-0.09999222	0.21179051
C	0.00312849	0.74678278	0.00001769
C	-1.11741044	-0.09043830	-0.21187559
C	0.67504138	-1.47602531	0.12615949
C	-0.68776259	-1.47019716	-0.12619553
C	0.00943573	2.22133174	0.00006120
H	0.16098844	2.77523862	0.91513931
H	-0.13743073	2.77658331	-0.91496423
H	-2.12537864	0.24427774	-0.39913261
H	2.12728450	0.22608694	0.39904807
H	-1.32594393	-2.33173208	-0.23680065
H	1.30583178	-2.34298821	0.23675235

$(S_0/S_1)_{CI}^{120^\circ}$

C	1.10609573	-0.09339735	0.22728137
C	0.00313000	0.74764452	0.00001325
C	-1.10698968	-0.08393172	-0.22725569
C	0.66229523	-1.49047254	0.13787976
C	-0.67513070	-1.48475105	-0.13795078
C	0.00946467	2.23160518	0.00004386
H	0.30442887	2.78315095	0.87993876
H	-0.28075365	2.78567743	-0.87984242
H	-2.11414583	0.24770967	-0.42582233
H	2.11604845	0.22961811	0.42586280
H	-1.31588807	-2.34137727	-0.26988455
H	1.29571839	-2.35254808	0.26973598

$(S_0/S_1)_{CI}^{130^\circ}$

C	1.09539460	-0.08859467	0.24322168
C	0.00311819	0.74526683	-0.00001062
C	-1.09624258	-0.07922823	-0.24322357
C	0.65106246	-1.50609700	0.14969430
C	-0.66403370	-1.50047540	-0.14978422
C	0.00952517	2.24735107	0.00004765
H	0.44023330	2.79506139	0.82337625
H	-0.41643356	2.79877752	-0.82327135
H	-2.10219407	0.25039679	-0.45282136
H	2.10411253	0.23241897	0.45289136
H	-1.30665469	-2.35243348	-0.30150557
H	1.28638577	-2.36351593	0.30138547

$(S_0/S_1)_{CI}^{140^\circ}$

C	1.08538639	-0.08569616	0.25979551
C	0.00310465	0.74125599	0.00001564
C	-1.08620398	-0.07640519	-0.25980604
C	0.64218788	-1.52070279	0.16097856
C	-0.65528417	-1.51515342	-0.16105199
C	0.00961230	2.26487743	0.00004826
H	0.56525203	2.80840758	0.74662927
H	-0.54136791	2.81316602	-0.74650639
H	-2.09000646	0.25165538	-0.48358959
H	2.09195536	0.23376360	0.48359593
H	-1.30021592	-2.36260150	-0.32768423
H	1.27985325	-2.37363908	0.32757509

$(S_0/S_1)_{CI}^{150^\circ}$

C	1.07645241	-0.08432715	0.27681264
C	0.00308500	0.73676576	0.00001281
C	-1.07725744	-0.07511347	-0.27682661

C	0.63539700	-1.53256959	0.17095038
C	-0.64859492	-1.52707577	-0.17102583
C	0.00968011	2.28105261	0.00005011
H	0.67656603	2.82071742	0.65123500
H	-0.65257168	2.82642569	-0.65110842
H	-2.07768029	0.25212370	-0.51768673
H	2.07963402	0.23433842	0.51769133
H	-1.29475924	-2.37120872	-0.34933510
H	1.27432242	-2.38220105	0.34923044

$(S_0/S_1)_{CI}^{160°}$

C	1.06851009	-0.08419171	0.29348682
C	0.00310754	0.73310080	-0.00009795
C	-1.06927959	-0.07502682	-0.29356604
C	0.63014880	-1.54103497	0.17997787
C	-0.64342337	-1.53558649	-0.18002484
C	0.00968188	2.29440945	0.00011080
H	0.77186582	2.83073128	0.53915033
H	-0.74778859	2.83726448	-0.53903456
H	-2.06485348	0.25166426	-0.55510023
H	2.06680649	0.23395314	0.55517752
H	-1.28970697	-2.37770059	-0.36726579
H	1.26920480	-2.38865499	0.36718609

$(S_0/S_1)_{CI}^{170°}$

C	1.06172309	-0.08411389	0.31123350
C	0.00310152	0.73048700	-0.00012156
C	-1.06249910	-0.07501348	-0.31131130
C	0.62643075	-1.54621942	0.18790065
C	-0.63975434	-1.54080349	-0.18793917
C	0.00972609	2.30264810	0.00012557
H	0.84768298	2.83680838	0.41364781
H	-0.82353314	2.84397988	-0.41356464
H	-2.05223702	0.25134565	-0.59497801
H	2.05415590	0.23375633	0.59512364
H	-1.28588778	-2.38151417	-0.38189451
H	1.26536445	-2.39243306	0.38177806

$(S_0/S_1)_{CI}^{180°}$

C	1.05641089	-0.08405554	0.32778416
C	0.00317351	0.72936318	-0.00035683
C	-1.05711843	-0.07499320	-0.32816112
C	0.62307416	-1.54785111	0.19737171
C	-0.63660675	-1.54246960	-0.19682391
C	0.00970793	2.30553336	0.00035920
H	0.90022046	2.83849928	0.28531776
H	-0.87610622	2.84604407	-0.28502942
H	-2.04080043	0.25133166	-0.63237391
H	2.04282850	0.23384624	0.63206133
H	-1.28159774	-2.38273024	-0.39659215
H	1.26108752	-2.39359028	0.39644323

6.1.2 XMS-CASPT2

$(S_0)_{min}$

C	1.18697702	-0.11001068	-0.00011817
C	0.00324512	0.77414456	-0.00003803
C	-1.18799472	-0.09988617	-0.00003944
C	0.73577148	-1.40961204	0.00011683
C	-0.74789246	-1.40328695	-0.00005270
C	0.00906543	2.13844482	-0.00000703
H	-0.92702039	2.70605788	-0.00002448
H	0.94993996	2.69808420	0.00020985
H	-2.21873236	0.25641173	-0.00002258

H	2.22071175	0.23750223	-0.00017235
H	-1.37279915	-2.29864349	0.00004219
H	1.35300166	-2.31027821	0.00010590

$(S_1)_{min}$

C	1.13154842	-0.09175799	-0.00001064
C	0.00318162	0.76079999	0.00000183
C	-1.13241537	-0.08210345	0.00001043
C	0.68589702	-1.49440771	-0.00000589
C	-0.69874294	-1.48850298	0.00000614
C	0.00945345	2.23158332	0.00001186
H	-0.92953768	2.78951666	-0.00009503
H	0.95316852	2.78148865	0.00008013
H	-2.17230796	0.25384037	0.00001856
H	2.17426832	0.23530462	-0.00001492
H	-1.36385176	-2.35264388	0.00001501
H	1.34361172	-2.36418971	-0.00001749

$(S_0/S_1)_{MECI}$

C	1.12771588	-0.09416982	-0.13193254
C	0.00294119	0.75929190	-0.00088135
C	-1.12865610	-0.08472511	0.13100163
C	0.67952734	-1.49959294	-0.08183013
C	-0.69194925	-1.49393145	0.08198488
C	0.00917629	2.23484318	0.00041489
H	-0.43212588	2.80020509	-0.82665184
H	0.45548575	2.79438972	0.82879260
H	-2.15821903	0.25458647	0.26507532
H	2.16008844	0.23660826	-0.26568183
H	-1.35102367	-2.35872396	0.16721808
H	1.33131241	-2.36985346	-0.16750974

$(S_0/S_1)_{CI}^{0^\circ}$

C	1.09356673	-0.08469778	-0.00118790
C	0.00308333	0.74619324	-0.00034490
C	-1.09440847	-0.07541081	0.00005920
C	0.65474705	-1.58494183	-0.00134038
C	-0.66838619	-1.57934397	-0.00094370
C	0.00996530	2.34821125	0.00008055
H	-0.93568298	2.88936116	0.00076351
H	0.96024972	2.88117519	-0.00031716
H	-2.14062773	0.24809373	-0.00007863
H	2.14250145	0.22987382	0.00116510
H	-1.36917827	-2.41404500	0.00107393
H	1.34844340	-2.42554110	0.00107037

$(S_0/S_1)_{CI}^{10^\circ}$

C	1.09348171	-0.08473589	0.00720006
C	0.00308848	0.74615271	0.00044661
C	-1.09435442	-0.07539657	-0.00506171
C	0.65469984	-1.58486671	0.00590858
C	-0.66829000	-1.57919869	-0.00868613
C	0.00996965	2.34812515	-0.00076021
H	-0.93549457	2.88921997	0.02073994
H	0.96004144	2.88104946	-0.02306931
H	-2.14056730	0.24806982	0.00339959
H	2.14240726	0.22987054	0.00106894
H	-1.36908606	-2.41388446	-0.00922772
H	1.34837731	-2.42547744	0.00804134

$(S_0/S_1)_{CI}^{20^\circ}$

C	1.09585791	-0.08414106	0.03602255
C	0.00318292	0.74906814	-0.00104364
C	-1.09680828	-0.07478804	-0.04149112
C	0.65644836	-1.57750473	0.01767131

C	-0.66987449	-1.57167995	-0.01465882
C	0.00993651	2.33466636	0.00218296
H	-0.88809750	2.87816006	0.29539258
H	0.91250280	2.87176413	-0.28880490
H	-2.14117787	0.24998244	-0.09535634
H	2.14323725	0.23159575	0.08460642
H	-1.36864384	-2.40805524	-0.02431174
H	1.34770956	-2.42013998	0.02979071

$(S_0/S_1)_{CI}^{30^\circ}$

C	1.09927765	-0.08352140	0.05447595
C	0.00323234	0.75226024	-0.00166588
C	-1.09992934	-0.07429425	-0.06273015
C	0.65803168	-1.56742924	0.02689935
C	-0.67196565	-1.56159421	-0.02276713
C	0.01005493	2.31803063	0.00297700
H	-0.82990100	2.86457460	0.43284014
H	0.85432992	2.86063298	-0.42343964
H	-2.14191924	0.25175624	-0.14158814
H	2.14446509	0.23379387	0.12648901
H	-1.36631214	-2.40170911	-0.03687869
H	1.34490910	-2.41357248	0.04538815

$(S_0/S_1)_{CI}^{40^\circ}$

C	1.10448299	-0.08374166	0.07322282
C	0.00339068	0.75587488	-0.00185251
C	-1.10490525	-0.07441026	-0.08222550
C	0.66116049	-1.55379304	0.03780874
C	-0.67525690	-1.54781067	-0.03333602
C	0.01003693	2.29702281	0.00303655
H	-0.75086050	2.84736329	0.55881945
H	0.77505936	2.84562436	-0.54881428
H	-2.14397310	0.25390730	-0.18186226
H	2.14690414	0.23572750	0.16653047
H	-1.36405787	-2.39239756	-0.05743142
H	1.34229236	-2.40443907	0.06610393

$(S_0/S_1)_{CI}^{50^\circ}$

C	1.11161341	-0.08548110	0.09278777
C	0.00357671	0.75898348	-0.00152934
C	-1.11168818	-0.07592793	-0.10018591
C	0.66586127	-1.53637797	0.05088704
C	-0.67977598	-1.53043270	-0.04749223
C	0.00972060	2.27359635	0.00235187
H	-0.65320750	2.82867141	0.66985956
H	0.67670633	2.82776920	-0.66187081
H	-2.14752509	0.25549786	-0.21520728
H	2.15080177	0.23681964	0.20345288
H	-1.36066306	-2.38107188	-0.08616078
H	1.33885305	-2.39311848	0.09310720

$(S_0/S_1)_{CI}^{60^\circ}$

C	1.12011394	-0.08934132	0.11269163
C	0.00327794	0.76023162	-0.00068037
C	-1.12082426	-0.07977331	-0.11615610
C	0.67243558	-1.51650640	0.06582243
C	-0.68594180	-1.51062185	-0.06460579
C	0.00977225	2.25091917	0.00118423
H	-0.53803247	2.81142637	0.76394632
H	0.56227556	2.80907848	-0.75993626
H	-2.15344850	0.25570119	-0.24116089
H	2.15570207	0.23732283	0.23651739
H	-1.35579310	-2.36893183	-0.12552813
H	1.33473612	-2.38057708	0.12790552

$(S_0/S_1)_{CI}^{70^\circ}$

C	1.12578709	-0.09401203	0.13022900
C	0.00050837	0.75897881	0.00080132
C	-1.13012222	-0.08699773	-0.12776412
C	0.67905135	-1.49896534	0.08035356
C	-0.69212595	-1.49361749	-0.08185430
C	0.01115405	2.23566457	0.00073785
H	-0.42427240	2.80302029	0.82999785
H	0.45568092	2.79333043	-0.83043830
H	-2.16050387	0.25139704	-0.25770326
H	2.15793305	0.23895628	0.26014745
H	-1.34963838	-2.35947154	-0.16933924
H	1.33082133	-2.36935543	0.16483218

$(S_0/S_1)_{CI}^{80^\circ}$

C	1.13661515	-0.10231715	0.15323917
C	0.00311708	0.75421472	0.00132532
C	-1.13795938	-0.09226220	-0.14836424
C	0.68996947	-1.48179934	0.09663440
C	-0.70239725	-1.47562349	-0.09966318
C	0.00943092	2.22344163	-0.00062337
H	-0.27140507	2.78982039	0.89414007
H	0.29521377	2.78442380	-0.89720504
H	-2.16359127	0.25284008	-0.29160905
H	2.16511605	0.23373910	0.29739448
H	-1.34858405	-2.34783125	-0.20744898
H	1.32874791	-2.35971845	0.20218042

$(S_0/S_1)_{CI}^{90^\circ}$

C	1.13740156	-0.10540827	0.17631137
C	0.00333799	0.75130603	-0.00024033
C	-1.13787461	-0.09553223	-0.17767545
C	0.69362378	-1.47557223	0.10745381
C	-0.70640981	-1.47003786	-0.10921562
C	0.00936642	2.22178240	0.00049963
H	-0.13277724	2.78529073	0.92927100
H	0.15615629	2.78457173	-0.92788993
H	-2.16001944	0.25171616	-0.33499701
H	2.16219399	0.23302752	0.33574674
H	-1.34926504	-2.34563509	-0.21097879
H	1.32853942	-2.35658105	0.21171459

$(S_0/S_1)_{CI}^{100^\circ}$

C	1.12938194	-0.10225494	0.19979899
C	0.00346644	0.75420926	-0.00102437
C	-1.12921151	-0.09233959	-0.20473595
C	0.68647597	-1.48167011	0.11911021
C	-0.69957721	-1.47566565	-0.11710693
C	0.00904078	2.22334518	0.00142073
H	0.00863904	2.78514658	0.94197812
H	0.01371939	2.78907697	-0.93677137
H	-2.14965372	0.25281962	-0.38110020
H	2.15311230	0.23398257	0.37416850
H	-1.34788966	-2.34800954	-0.20979175
H	1.32676955	-2.35971251	0.21405404

$(S_0/S_1)_{CI}^{110^\circ}$

C	1.11620727	-0.09536424	0.21705468
C	0.00329427	0.75884325	-0.00042978
C	-1.11661008	-0.08564627	-0.21936682
C	0.67342504	-1.49679199	0.13043835
C	-0.68647989	-1.49091869	-0.12979850
C	0.00931719	2.23245973	0.00082211
H	0.15757661	2.79382619	0.92910140
H	-0.13431384	2.79689055	-0.92632874
H	-2.13746648	0.25461621	-0.40629661

H	2.14004578	0.23608413	0.40349385
H	-1.33976646	-2.35677662	-0.24416481
H	1.31904389	-2.36829439	0.24547488

$(S_0/S_1)_{CI}^{120^\circ}$

C	1.11049957	-0.08992993	0.22569615
C	0.00305792	0.76205285	0.00072364
C	-1.11193579	-0.08041593	-0.22345685
C	0.66764525	-1.50665704	0.13817399
C	-0.68046712	-1.50066189	-0.13985334
C	0.00978225	2.23489768	0.00006285
H	0.22541776	2.79973738	0.91117072
H	-0.20100051	2.79879386	-0.91283709
H	-2.13624829	0.25406268	-0.40555150
H	2.13727049	0.23574270	0.40997103
H	-1.33945562	-2.35852189	-0.27884005
H	1.31970738	-2.37017260	0.27474046

$(S_0/S_1)_{CI}^{130^\circ}$

C	1.08742445	-0.08529988	0.24898560
C	0.00305687	0.75899372	0.00177661
C	-1.08958829	-0.07604414	-0.24196289
C	0.64977935	-1.53629295	0.15490650
C	-0.66211109	-1.53050524	-0.15988778
C	0.01003710	2.27344549	-0.00146149
H	0.44391430	2.82853020	0.83330435
H	-0.41899813	2.82791970	-0.83912703
H	-2.11162857	0.25531743	-0.44615236
H	2.11131813	0.23714956	0.45814016
H	-1.32280770	-2.38135773	-0.32791064
H	1.30387687	-2.39292829	0.31938898

$(S_0/S_1)_{CI}^{140^\circ}$

C	1.07486902	-0.08351821	0.26491484
C	0.00278383	0.75584214	0.00207692
C	-1.07786863	-0.07451731	-0.25639056
C	0.64156308	-1.55359532	0.16566719
C	-0.65363382	-1.54796581	-0.17136034
C	0.01049025	2.29701694	-0.00200585
H	0.57230100	2.84635862	0.75517011
H	-0.54629320	2.84653103	-0.76272701
H	-2.09829651	0.25360259	-0.47630567
H	2.09646656	0.23611363	0.49184499
H	-1.31729070	-2.39274488	-0.35645832
H	1.29918242	-2.40419555	0.34557372

$(S_0/S_1)_{CI}^{150^\circ}$

C	1.06413274	-0.08317986	0.28078732
C	0.00253697	0.75226883	0.00183502
C	-1.06764796	-0.07440215	-0.27304614
C	0.63543605	-1.56714266	0.17504620
C	-0.64717335	-1.56203435	-0.18067534
C	0.01068508	2.31791809	-0.00198486
H	0.68637026	2.86111188	0.65935522
H	-0.65964672	2.86405682	-0.66629376
H	-2.08478509	0.25131929	-0.51311834
H	2.08201053	0.23441020	0.52849190
H	-1.31324004	-2.40225565	-0.37680098
H	1.29559483	-2.41314258	0.36640378

$(S_0/S_1)_{CI}^{160^\circ}$

C	1.05557830	-0.08356091	0.29724852
C	0.00266559	0.74910560	0.00145973
C	-1.05809323	-0.07485633	-0.29109676
C	0.63042539	-1.57720852	0.18379534

C	-0.64261442	-1.57156555	-0.18852384
C	0.01029000	2.33400542	-0.00159983
H	0.78264860	2.87234144	0.54752649
H	-0.75699128	2.87733939	-0.55290447
H	-2.07015428	0.24936300	-0.55467220
H	2.06852121	0.23227156	0.56756072
H	-1.31058658	-2.40855744	-0.39155817
H	1.29258399	-2.41974981	0.38276449

$(S_0/S_1)_{CI}^{170^\circ}$

C	1.04824608	-0.08410668	0.31397100
C	0.00278286	0.74689983	0.00076463
C	-1.04991492	-0.07542577	-0.31117703
C	0.62686271	-1.58291178	0.19152375
C	-0.63964693	-1.57740576	-0.19369517
C	0.00992254	2.34436700	-0.00041278
H	0.85962933	2.87893582	0.42333091
H	-0.83499306	2.88585774	-0.42495864
H	-2.05513225	0.24813025	-0.60104743
H	2.05515057	0.23100911	0.60726473
H	-1.30812033	-2.41272954	-0.40165738
H	1.28948669	-2.42369236	0.39609343

$(S_0/S_1)_{CI}^{180^\circ}$

C	1.04255653	-0.08412939	0.33023071
C	0.00262311	0.74616715	0.00032895
C	-1.04342780	-0.07613253	-0.32908568
C	0.62363948	-1.58502917	0.20002392
C	-0.63667606	-1.57900571	-0.20199710
C	0.00959675	2.34805051	0.00018681
H	0.91258164	2.88123693	0.29582903
H	-0.88844588	2.88946147	-0.29546406
H	-2.04178695	0.24743356	-0.64163513
H	2.04298090	0.23090331	0.64485288
H	-1.30407822	-2.41436837	-0.41297422
H	1.28470979	-2.42565992	0.40970393

6.1.3 MS-CASPT2

$(S_0)_{min}$

C	1.18619082	-0.10943183	-0.00000632
C	0.00323803	0.77401720	0.00000124
C	-1.18720644	-0.09931078	-0.00000210
C	0.73563867	-1.40992483	0.00000504
C	-0.74776217	-1.40359906	-0.00000457
C	0.00905546	2.13829699	0.00000648
H	-0.92676913	2.70572887	-0.00000137
H	0.94968499	2.69772768	0.00000361
H	-2.21782584	0.25621270	-0.00000164
H	2.21980499	0.23728852	-0.00001109
H	-1.37333876	-2.29822457	-0.00000226
H	1.35356271	-2.30985302	0.00001296

$(S_1)_{min}$

C	1.13108852	-0.09165082	0.00019491
C	0.00317421	0.75907776	-0.00005364
C	-1.13195462	-0.08200032	-0.00002809
C	0.68570668	-1.49509641	-0.00005580
C	-0.69855846	-1.48919337	0.00000239
C	0.00945778	2.23259711	-0.00002196
H	-0.92941765	2.79024591	-0.00008443
H	0.95305491	2.78221849	0.00015397
H	-2.17151088	0.25469772	0.00004503
H	2.17347847	0.23616899	-0.00021002
H	-1.36334345	-2.35329791	-0.00002058

H	1.34309783	-2.36483927	0.00007821

$(S_0/S_1)_{MECI}$

C	1.12852352	-0.09439436	-0.13125782
C	0.00309623	0.75848771	-0.00003673
C	-1.12938875	-0.08499156	0.13101733
C	0.68037836	-1.49855307	-0.08241876
C	-0.69297138	-1.49285068	0.08183003
C	0.00928959	2.23387712	0.00045674
H	-0.43191720	2.79837078	-0.82716277
H	0.45516299	2.79401662	0.82853784
H	-2.15857732	0.25521559	0.26534884
H	2.16052403	0.23724986	-0.26542120
H	-1.35090480	-2.35818051	0.16746968
H	1.33105809	-2.36931961	-0.16836321

$(S_0/S_1)_{CI}^{0°}$

C	1.09456282	-0.08501410	-0.00092931
C	0.00324595	0.74415058	-0.00052988
C	-1.09526141	-0.07547903	-0.00118683
C	0.65524363	-1.58168187	-0.00194063
C	-0.66884591	-1.57592732	-0.00038161
C	0.00990098	2.34378297	0.00049532
H	-0.93550228	2.88562245	0.00072849
H	0.95964377	2.87795556	0.00095173
H	-2.14094724	0.24997960	-0.00067197
H	2.14302241	0.23140746	-0.00034267
H	-1.36753225	-2.41208051	0.00196235
H	1.34674287	-2.42378789	0.00184499

$(S_0/S_1)_{CI}^{10°}$

C	1.09430204	-0.08486271	0.00741884
C	0.00308218	0.74383306	0.00052284
C	-1.09515896	-0.07553266	-0.00517062
C	0.65512480	-1.58122517	0.00602319
C	-0.66868606	-1.57560025	-0.00880415
C	0.00993365	2.34333919	-0.00064378
H	-0.93509456	2.88539121	0.02072038
H	0.95959247	2.87722875	-0.02279321
H	-2.14080918	0.25012464	0.00363585
H	2.14269810	0.23186210	-0.00007041
H	-1.36696167	-2.41204654	-0.00984401
H	1.34625052	-2.42358373	0.00900505

$(S_0/S_1)_{CI}^{20°}$

C	1.09718915	-0.08430547	0.03615765
C	0.00339479	0.74703035	-0.00118392
C	-1.09742402	-0.07482917	-0.04177877
C	0.65653687	-1.57398194	0.01779925
C	-0.67036432	-1.56795917	-0.01557719
C	0.00983764	2.33015867	0.00192119
H	-0.88821556	2.87413569	0.29490528
H	0.91187931	2.86870002	-0.28880444
H	-2.14120441	0.25190860	-0.09448457
H	2.14398510	0.23330775	0.08475578
H	-1.36615409	-2.40655313	-0.02382800
H	1.34481287	-2.41868431	0.03011770

$(S_0/S_1)_{CI}^{30°}$

C	1.10002614	-0.08353738	0.05469389
C	0.00311795	0.75065546	-0.00141530
C	-1.10089644	-0.07442142	-0.06170495
C	0.65854928	-1.56422077	0.02794659
C	-0.67233608	-1.55855524	-0.02375940
C	0.01006649	2.31359453	0.00253587

H	-0.82929523	2.86130897	0.43232057
H	0.85398730	2.85658208	-0.42434391
H	-2.14245686	0.25356199	-0.13969903
H	2.14471159	0.23587975	0.12612751
H	-1.36445013	-2.40012665	-0.03921261
H	1.34324933	-2.41179343	0.04651074

$(S_0/S_1)_{CI}^{40^\circ}$

C	1.10555036	-0.08379234	0.07284756
C	0.00331565	0.75460590	-0.00167584
C	-1.10597622	-0.07457590	-0.08089222
C	0.66182878	-1.55097642	0.03829930
C	-0.67584079	-1.54496890	-0.03382169
C	0.00994137	2.29338656	0.00266251
H	-0.75026159	2.84456614	0.55872457
H	0.77447132	2.84201004	-0.55000171
H	-2.14463263	0.25519979	-0.18044175
H	2.14749565	0.23722654	0.16656275
H	-1.36259588	-2.39087025	-0.05980525
H	1.34097732	-2.40288327	0.06754176

$(S_0/S_1)_{CI}^{50^\circ}$

C	1.11253933	-0.08566679	0.09291361
C	0.00350884	0.75777684	-0.00136658
C	-1.11299094	-0.07607885	-0.09913552
C	0.66653733	-1.53398704	0.05196828
C	-0.68037658	-1.52802588	-0.04818313
C	0.00989743	2.27010882	0.00174857
H	-0.65243970	2.82606806	0.66914878
H	0.67643458	2.82443292	-0.66287330
H	-2.14868403	0.25705009	-0.21238201
H	2.15137128	0.23847619	0.20371832
H	-1.35952615	-2.37963426	-0.08959411
H	1.33800193	-2.39159222	0.09403708

$(S_0/S_1)_{CI}^{60^\circ}$

C	1.12122063	-0.08964616	0.11248431
C	0.00337080	0.75939934	-0.00051573
C	-1.12176313	-0.07993251	-0.11512283
C	0.67329963	-1.51470951	0.06629791
C	-0.68675071	-1.50881718	-0.06535726
C	0.00962633	2.24884783	0.00088471
H	-0.53770503	2.80965643	0.76372507
H	0.56152062	2.80689509	-0.76071484
H	-2.15405451	0.25668828	-0.23975710
H	2.15653651	0.23790259	0.23623336
H	-1.35519289	-2.36783934	-0.12760071
H	1.33416505	-2.37951698	0.12944308

$(S_0/S_1)_{CI}^{70^\circ}$

C	1.12357459	-0.09509135	0.12150032
C	0.00396210	0.75751502	0.00058293
C	-1.12376619	-0.08445529	-0.11984066
C	0.67444362	-1.50550436	0.07436844
C	-0.68756378	-1.49958115	-0.07424314
C	0.00915961	2.24636242	-0.00005335
H	-0.49300847	2.80658924	0.79580509
H	0.51536002	2.80207515	-0.79660443
H	-2.15390261	0.25818591	-0.24046058
H	2.15683547	0.23821337	0.24170551
H	-1.34339463	-2.36672692	-0.15389924
H	1.32257359	-2.37865417	0.15113907

$(S_0/S_1)_{CI}^{80^\circ}$

C	1.13683801	-0.10212824	0.15328014
C	0.00293210	0.75372986	0.00121151
C	-1.13833743	-0.09237572	-0.14860224
C	0.69069779	-1.48165980	0.09670710
C	-0.70302923	-1.47559669	-0.09934484
C	0.00945342	2.22322076	-0.00071367
H	-0.27136276	2.78954698	0.89397717
H	0.29539537	2.78402785	-0.89727233
H	-2.16360572	0.25319328	-0.29256251
H	2.16486407	0.23477542	0.29808739
H	-1.34860917	-2.34808896	-0.20629135
H	1.32903688	-2.35971686	0.20152359

$(S_0/S_1)_{CI}^{90^\circ}$

C	1.13690365	-0.10541411	0.17738062
C	0.00302274	0.75081845	0.00032229
C	-1.13819785	-0.09575033	-0.17681578
C	0.69402467	-1.47630011	0.10928863
C	-0.70639716	-1.47026331	-0.11066461
C	0.00943527	2.22230497	0.00040188
H	-0.13269327	2.78597047	0.92914082
H	0.15648106	2.78469747	-0.92834075
H	-2.16014865	0.25169223	-0.33691259
H	2.16202585	0.23354618	0.33545743
H	-1.34981211	-2.34546691	-0.20880871
H	1.32962914	-2.35690713	0.20955073

$(S_0/S_1)_{CI}^{100^\circ}$

C	1.12862676	-0.10121022	0.19905911
C	0.00212424	0.75383743	-0.00168768
C	-1.13080474	-0.09241009	-0.20593916
C	0.68798737	-1.48249674	0.11816292
C	-0.69974415	-1.47417833	-0.11528089
C	0.00982017	2.22206836	0.00172236
H	0.01010985	2.78394127	0.94241994
H	0.01544171	2.78772161	-0.93676674
H	-2.15142610	0.25200720	-0.38131940
H	2.15198367	0.23602374	0.37406966
H	-1.34791953	-2.34657981	-0.20951640
H	1.32807408	-2.35979656	0.21507623

$(S_0/S_1)_{CI}^{110^\circ}$

C	1.11683594	-0.09548036	0.21736611
C	0.00320248	0.75811830	-0.00056854
C	-1.11726332	-0.08589469	-0.21983509
C	0.67431774	-1.49589655	0.13077194
C	-0.68746931	-1.48997813	-0.12970834
C	0.00937442	2.23159172	0.00063482
H	0.15747999	2.79276121	0.92893817
H	-0.13397681	2.79592269	-0.92649512
H	-2.13789044	0.25512335	-0.40633478
H	2.14044279	0.23683243	0.40324243
H	-1.33980838	-2.35634472	-0.24349200
H	1.31902822	-2.36782739	0.24548035

$(S_0/S_1)_{CI}^{120^\circ}$

C	1.11130014	-0.09002641	0.22618219
C	0.00287312	0.76082690	0.00050512
C	-1.11262189	-0.08082962	-0.22422687
C	0.66822336	-1.50531360	0.13846606
C	-0.68105314	-1.49901617	-0.14041670
C	0.00984571	2.23374296	-0.00015405
H	0.22694046	2.79815084	0.91080243
H	-0.20218560	2.79746712	-0.91279264
H	-2.13676215	0.25435499	-0.40624392

H	2.13769880	0.23668587	0.41082362
H	-1.33909463	-2.35761845	-0.27718409
H	1.31910912	-2.36949657	0.27423879

$(S_0/S_1)_{CI}^{130^\circ}$

C	1.08817327	-0.08542146	0.24929611
C	0.00280615	0.75797073	0.00155204
C	-1.09070801	-0.07638705	-0.24295260
C	0.65061874	-1.53381019	0.15483547
C	-0.66318166	-1.52811096	-0.15850941
C	0.01029141	2.27056793	-0.00133685
H	0.44398670	2.82554799	0.83362676
H	-0.41816297	2.82566727	-0.83891032
H	-2.11207010	0.25612064	-0.44871471
H	2.11166465	0.23857445	0.45808634
H	-1.32236321	-2.38021151	-0.32468787
H	1.30321835	-2.39157998	0.31771496

$(S_0/S_1)_{CI}^{140^\circ}$

C	1.07572903	-0.08362825	0.26541076
C	0.00276321	0.75457552	0.00180426
C	-1.07853580	-0.07470560	-0.25765933
C	0.64213548	-1.55078320	0.16556327
C	-0.65456002	-1.54510692	-0.17033845
C	0.01050269	2.29322959	-0.00200641
H	0.57176092	2.84290940	0.75546081
H	-0.54558946	2.84366782	-0.76269760
H	-2.09834365	0.25514117	-0.47826868
H	2.09708157	0.23747432	0.49175455
H	-1.31680951	-2.39121134	-0.35298662
H	1.29813885	-2.40263466	0.34396335

$(S_0/S_1)_{CI}^{150^\circ}$

C	1.06552136	-0.08364724	0.28133479
C	0.00312337	0.75032770	0.00155785
C	-1.06801581	-0.07412896	-0.27461559
C	0.63593313	-1.56412643	0.17465128
C	-0.64842420	-1.55853778	-0.17841164
C	0.01041600	2.31386052	-0.00208665
H	0.68538302	2.85745494	0.65985732
H	-0.65973890	2.86004005	-0.66683793
H	-2.08430426	0.25391031	-0.51549240
H	2.08308044	0.23552826	0.52870152
H	-1.31289450	-2.40007863	-0.37283388
H	1.29419364	-2.41167489	0.36417524

$(S_0/S_1)_{CI}^{160^\circ}$

C	1.05613545	-0.08374609	0.29725011
C	0.00259894	0.74728513	0.00093065
C	-1.05883158	-0.07508797	-0.29267477
C	0.63105464	-1.57371236	0.18311793
C	-0.64349991	-1.56837540	-0.18713355
C	0.01066647	2.32977283	-0.00152412
H	0.78271879	2.86832009	0.54809812
H	-0.75590281	2.87433933	-0.55285418
H	-2.07014861	0.25112977	-0.55686374
H	2.06866286	0.23425095	0.56678217
H	-1.30970836	-2.40717325	-0.38716183
H	1.29052742	-2.41807519	0.38203311

$(S_0/S_1)_{CI}^{170^\circ}$

C	1.04886679	-0.08438925	0.31414366
C	0.00262815	0.74481354	0.00059207
C	-1.05117483	-0.07571429	-0.31135626
C	0.62715853	-1.57943007	0.19203603

C	-0.64023936	-1.57415053	-0.19376477
C	0.01048233	2.34017157	-0.00079777
H	0.86015530	2.87520270	0.42263654
H	-0.83383998	2.88290614	-0.42514865
H	-2.05587867	0.24963515	-0.60123850
H	2.05534213	0.23294756	0.60678594
H	-1.30669016	-2.41121002	-0.39989853
H	1.28746307	-2.42185465	0.39601015

$(S_0/S_1)_{CI}^{180^\circ}$

C	1.04386131	-0.08484499	0.32991508
C	0.00323109	0.74407028	0.00069317
C	-1.04524817	-0.07544254	-0.32711429
C	0.62377731	-1.58150297	0.20132760
C	-0.63732466	-1.57617983	-0.20185134
C	0.01015742	2.34342521	-0.00089603
H	0.90881430	2.87825076	0.30524237
H	-0.88409443	2.88495198	-0.30819164
H	-2.04429428	0.24998078	-0.63601177
H	2.04487661	0.23173076	0.64163540
H	-1.30343798	-2.41221402	-0.41298625
H	1.28395477	-2.42329756	0.40823762

6.1.4 RMS-CASPT2

$(S_0)_{min}$

C	1.18619083	-0.10943184	-0.00000631
C	0.00323804	0.77401720	0.00000124
C	-1.18720643	-0.09931077	-0.00000210
C	0.73563867	-1.40992483	0.00000504
C	-0.74776217	-1.40359905	-0.00000457
C	0.00905546	2.13829699	0.00000648
H	-0.92676914	2.70572885	-0.00000136
H	0.94968498	2.69772770	0.00000361
H	-2.21782583	0.25621271	-0.00000164
H	2.21980500	0.23728851	-0.00001109
H	-1.37333876	-2.29822456	-0.00000226
H	1.35356271	-2.30985302	0.00001296

$(S_1)_{min}$

C	1.13108852	-0.09165082	0.00019491
C	0.00317421	0.75907776	-0.00005364
C	-1.13195462	-0.08200032	-0.00002809
C	0.68570668	-1.49509641	-0.00005580
C	-0.69855846	-1.48919337	0.00000239
C	0.00945778	2.23259711	-0.00002197
H	-0.92941765	2.79024591	-0.00008443
H	0.95305491	2.78221849	0.00015397
H	-2.17151088	0.25469773	0.00004503
H	2.17347847	0.23616898	-0.00021002
H	-1.36334345	-2.35329791	-0.00002058
H	1.34309783	-2.36483927	0.00007821

$(S_0/S_1)_{MECI}$

C	1.12851141	-0.09439741	-0.13127641
C	0.00309890	0.75847394	0.00001037
C	-1.12938264	-0.08499895	0.13109390
C	0.68038944	-1.49853419	-0.08243929
C	-0.69298306	-1.49283136	0.08183107
C	0.00926764	2.23387305	0.00046678
H	-0.43184658	2.79831374	-0.82723944
H	0.45512045	2.79404789	0.82853213
H	-2.15857064	0.25522193	0.26540789
H	2.16051835	0.23725112	-0.26540724
H	-1.35090670	-2.35817974	0.16738932

H	1.33105678	-2.36931212	-0.16836909

$(S_0/S_1)_{CI}^{0^\circ}$

C	1.09447444	-0.08495145	-0.00074904
C	0.00315620	0.74418374	-0.00035658
C	-1.09525137	-0.07554818	-0.00073303
C	0.65524430	-1.58164100	-0.00144474
C	-0.66883920	-1.57595811	0.00083114
C	0.00992409	2.34376191	0.00038951
H	-0.93537841	2.88576598	0.00064735
H	0.95973163	2.87782284	0.00063357
H	-2.14093966	0.24989821	-0.00112554
H	2.14291113	0.23153264	-0.00020630
H	-1.36747067	-2.41216153	0.00121305
H	1.34671086	-2.42377714	0.00090059

$(S_0/S_1)_{CI}^{10^\circ}$

C	1.09440575	-0.08486305	0.00736909
C	0.00311543	0.74383154	0.00058006
C	-1.09519301	-0.07551815	-0.00485641
C	0.65513835	-1.58131018	0.00618142
C	-0.66870390	-1.57561258	-0.00814385
C	0.00991892	2.34337618	-0.00073249
H	-0.93515187	2.88536932	0.02074121
H	0.95956191	2.87729351	-0.02308896
H	-2.14084574	0.25012516	0.00342888
H	2.14279844	0.23188470	0.00028218
H	-1.36705405	-2.41199242	-0.01024406
H	1.34628311	-2.42365615	0.00848292

$(S_0/S_1)_{CI}^{20^\circ}$

C	1.09728735	-0.08410127	0.03629264
C	0.00340821	0.74708694	-0.00108636
C	-1.09730753	-0.07465861	-0.04160259
C	0.65656580	-1.57386385	0.01803693
C	-0.67035735	-1.56817331	-0.01507071
C	0.00947931	2.32981957	0.00190102
H	-0.88827653	2.87438323	0.29472792
H	0.91174963	2.86793395	-0.28899958
H	-2.14102198	0.25223859	-0.09459616
H	2.14411694	0.23338083	0.08485597
H	-1.36630240	-2.40661818	-0.02415597
H	1.34493190	-2.41850002	0.02969688

$(S_0/S_1)_{CI}^{30^\circ}$

C	1.10030455	-0.08358548	0.05432245
C	0.00311911	0.75059028	-0.00157333
C	-1.10085525	-0.07451989	-0.06165521
C	0.65850470	-1.56425221	0.02762377
C	-0.67240307	-1.55841095	-0.02345481
C	0.00994461	2.31388306	0.00239328
H	-0.82940074	2.86143284	0.43236014
H	0.85372744	2.85691737	-0.42464137
H	-2.14240645	0.25337869	-0.13964792
H	2.14496788	0.23560757	0.12663042
H	-1.36432334	-2.40019406	-0.03913322
H	1.34309392	-2.41191934	0.04677579

$(S_0/S_1)_{CI}^{40^\circ}$

C	1.10541113	-0.08383887	0.07316997
C	0.00317135	0.75450144	-0.00171482
C	-1.10601935	-0.07471625	-0.08104952
C	0.66187803	-1.55092237	0.03874639
C	-0.67575404	-1.54501815	-0.03439334
C	0.00997193	2.29348138	0.00241881

H	-0.75032667	2.84473759	0.55826762
H	0.77463628	2.84206742	-0.55011657
H	-2.14478548	0.25501541	-0.17966961
H	2.14737469	0.23733612	0.16616920
H	-1.36245381	-2.39098791	-0.05982515
H	1.34116930	-2.40272792	0.06799702

$(S_0/S_1)_{CI}^{50^\circ}$

C	1.11261193	-0.08561437	0.09256842
C	0.00335462	0.75790000	-0.00146407
C	-1.11294595	-0.07627676	-0.09905939
C	0.66665843	-1.53388897	0.05161505
C	-0.68036725	-1.52799590	-0.04811630
C	0.00972364	2.27065232	0.00175004
H	-0.65252285	2.82651645	0.66933900
H	0.67622947	2.82477087	-0.66306550
H	-2.14846538	0.25648776	-0.21326679
H	2.15129627	0.23835098	0.20328965
H	-1.35893140	-2.38011507	-0.08835262
H	1.33763181	-2.39185942	0.09476250

$(S_0/S_1)_{CI}^{60^\circ}$

C	1.12117547	-0.08957186	0.11247887
C	0.00328259	0.75945553	-0.00059789
C	-1.12174986	-0.08005603	-0.11509743
C	0.67339577	-1.51470861	0.06645509
C	-0.68662814	-1.50885467	-0.06515314
C	0.00951617	2.24882737	0.00065062
H	-0.53777082	2.80986180	0.76338041
H	0.56138066	2.80682545	-0.76099399
H	-2.15407879	0.25641229	-0.23966679
H	2.15644310	0.23812344	0.23606952
H	-1.35502306	-2.36793472	-0.12727113
H	1.33433024	-2.37945212	0.12974584

$(S_0/S_1)_{CI}^{70^\circ}$

C	1.12924972	-0.09541656	0.13098661
C	0.00329824	0.75823119	0.00070698
C	-1.12980311	-0.08554199	-0.12792008
C	0.68069133	-1.49768541	0.08161856
C	-0.69340041	-1.49132524	-0.08296053
C	0.00914103	2.23363510	-0.00077169
H	-0.42259353	2.79816948	0.83239455
H	0.44531080	2.79308792	-0.83503379
H	-2.15918099	0.25524538	-0.25816413
H	2.16155610	0.23609214	0.26208073
H	-1.35102396	-2.35678963	-0.17038259
H	1.33102811	-2.36877451	0.16744536

$(S_0/S_1)_{CI}^{80^\circ}$

C	1.13696614	-0.10220937	0.15330386
C	0.00317920	0.75379345	0.00118394
C	-1.13819611	-0.09230274	-0.14846432
C	0.69074391	-1.48159698	0.09694124
C	-0.70291215	-1.47561232	-0.09937356
C	0.00920185	2.22340936	-0.00102031
H	-0.27186629	2.78942781	0.89390270
H	0.29498121	2.78385195	-0.89793275
H	-2.16363321	0.25312072	-0.29193402
H	2.16527293	0.23418899	0.29768720
H	-1.34899702	-2.34778721	-0.20579746
H	1.32953287	-2.35935578	0.20150345

$(S_0/S_1)_{CI}^{90^\circ}$

C	1.13734465	-0.10522183	0.17664079
C	0.00304622	0.75069775	-0.00007529
C	-1.13820048	-0.09569858	-0.17686351
C	0.69409835	-1.47621977	0.10859510
C	-0.70642806	-1.47018465	-0.11040019
C	0.00936862	2.22194977	0.00003643
H	-0.13247618	2.78563443	0.92874177
H	0.15603507	2.78474555	-0.92845993
H	-2.16031791	0.25183479	-0.33568302
H	2.16229494	0.23393046	0.33578793
H	-1.34968574	-2.34552006	-0.20872952
H	1.32919387	-2.35701997	0.21040941

$(S_0/S_1)_{CI}^{100^\circ}$

C	1.13525879	-0.10544469	0.18492382
C	0.00296036	0.75118455	0.00028452
C	-1.13704018	-0.09583979	-0.18367443
C	0.69345305	-1.47683334	0.11481274
C	-0.70562134	-1.47017324	-0.11444550
C	0.00948574	2.22245356	-0.00032396
H	-0.11295716	2.78623693	0.93099320
H	0.13687951	2.78512944	-0.93176981
H	-2.16038811	0.25167457	-0.33449989
H	2.16158025	0.23357286	0.33491826
H	-1.34840130	-2.34546137	-0.21598425
H	1.32906373	-2.35757160	0.21476527

$(S_0/S_1)_{CI}^{110^\circ}$

C	1.11697029	-0.09547449	0.21712225
C	0.00328991	0.75803647	-0.00057031
C	-1.11729112	-0.08592987	-0.21946098
C	0.67448306	-1.49580714	0.13078270
C	-0.68738009	-1.49003133	-0.12947636
C	0.00916761	2.23170066	0.00044774
H	0.15742852	2.79294601	0.92868898
H	-0.13457337	2.79572681	-0.92681421
H	-2.13791249	0.25509195	-0.40591675
H	2.14057420	0.23685904	0.40295389
H	-1.33964780	-2.35644827	-0.24334043
H	1.31916463	-2.36774198	0.24558345

$(S_0/S_1)_{CI}^{120^\circ}$

C	1.11137504	-0.09014903	0.22618100
C	0.00307526	0.76078378	0.00065041
C	-1.11262646	-0.08075530	-0.22378623
C	0.66836504	-1.50519131	0.13875189
C	-0.68101323	-1.49916867	-0.13991976
C	0.00960985	2.23380904	-0.00028715
H	0.22668271	2.79867843	0.91037602
H	-0.20280756	2.79704709	-0.91315035
H	-2.13673335	0.25448632	-0.40583789
H	2.13789526	0.23646278	0.41038376
H	-1.33895719	-2.35773799	-0.27736924
H	1.31940797	-2.36933729	0.27400751

$(S_0/S_1)_{CI}^{130^\circ}$

C	1.08828229	-0.08534360	0.24915507
C	0.00280205	0.75792795	0.00147264
C	-1.09065653	-0.07643013	-0.24280837
C	0.65078543	-1.53372440	0.15487376
C	-0.66307236	-1.52816567	-0.15832953
C	0.01005122	2.27051971	-0.00158158
H	0.44377592	2.82563802	0.83327799
H	-0.41859746	2.82545791	-0.83915568
H	-2.11210659	0.25616650	-0.44797852

H	2.11172600	0.23877457	0.45793426
H	-1.32209966	-2.38036891	-0.32449534
H	1.30338303	-2.39152409	0.31763528

$(S_0/S_1)_{CI}^{140^\circ}$

C	1.07576951	-0.08354826	0.26520095
C	0.00267408	0.75458058	0.00176633
C	-1.07863563	-0.07475053	-0.25738740
C	0.64233088	-1.55069093	0.16558907
C	-0.65453552	-1.54518282	-0.16962183
C	0.01034630	2.29325406	-0.00216149
H	0.57176276	2.84301068	0.75515179
H	-0.54596310	2.84359208	-0.76279677
H	-2.09847407	0.25484602	-0.47825631
H	2.09705177	0.23771711	0.49161919
H	-1.31656132	-2.39138779	-0.35264138
H	1.29850769	-2.40251235	0.34353783

$(S_0/S_1)_{CI}^{150^\circ}$

C	1.06547186	-0.08343308	0.28134704
C	0.00299547	0.75067651	0.00167946
C	-1.06763585	-0.07436494	-0.27405975
C	0.63596971	-1.56429360	0.17492380
C	-0.64816898	-1.55868420	-0.17874726
C	0.01011027	2.31355337	-0.00216863
H	0.68470928	2.85827317	0.65914279
H	-0.66004703	2.85981919	-0.66668374
H	-2.08403863	0.25353155	-0.51456747
H	2.08317770	0.23558624	0.52817765
H	-1.31280144	-2.40008647	-0.37337170
H	1.29453098	-2.41164990	0.36432780

$(S_0/S_1)_{CI}^{160^\circ}$

C	1.05625901	-0.08372011	0.29769582
C	0.00259290	0.74726050	0.00147685
C	-1.05920791	-0.07497867	-0.29122922
C	0.63098563	-1.57382518	0.18451730
C	-0.64317798	-1.56866829	-0.18782897
C	0.01046033	2.32958668	-0.00186256
H	0.78224993	2.86934011	0.54673005
H	-0.75645286	2.87355959	-0.55310825
H	-2.07061377	0.25130409	-0.55519176
H	2.06892702	0.23430941	0.56684064
H	-1.30904638	-2.40728530	-0.38958303
H	1.29129741	-2.41795500	0.38154312

$(S_0/S_1)_{CI}^{170^\circ}$

C	1.04910161	-0.08454790	0.31418200
C	0.00287499	0.74483795	0.00072260
C	-1.05043183	-0.07595164	-0.31107203
C	0.62727644	-1.57945802	0.19198165
C	-0.63996809	-1.57392182	-0.19403680
C	0.00977436	2.34021289	-0.00087061
H	0.85906466	2.87610196	0.42241842
H	-0.83495199	2.88236078	-0.42528532
H	-2.05520060	0.24973209	-0.60032384
H	2.05574629	0.23261015	0.60633696
H	-1.30637214	-2.41102580	-0.40013322
H	1.28735962	-2.42202281	0.39608018

$(S_0/S_1)_{CI}^{180^\circ}$

C	1.04482658	-0.08454785	0.33075642
C	0.00341674	0.74396860	0.00059377
C	-1.04383521	-0.07609974	-0.32823158
C	0.62386835	-1.58236644	0.20083201

C	-0.63742678	-1.57541422	-0.20171265
C	0.00881358	2.34382787	-0.00078879
H	0.91126851	2.87843659	0.29436546
H	-0.88991468	2.88418108	-0.29683901
H	-2.04213992	0.24970885	-0.63947336
H	2.04559729	0.23131550	0.64430890
H	-1.30425364	-2.41062323	-0.41462440
H	1.28405250	-2.42345917	0.41081321

6.1.5 XDW-CASPT2

$(S_0)_{min}$

C	1.18619660	-0.10943100	-0.00010675
C	0.00323880	0.77404324	-0.00000406
C	-1.18721164	-0.09930877	0.00007750
C	0.73565662	-1.40994152	0.00000114
C	-0.74778025	-1.40361483	-0.00000664
C	0.00905451	2.13828616	-0.00000086
H	-0.92672873	2.70570502	-0.00032751
H	0.94964184	2.69770530	0.00038754
H	-2.21777293	0.25624331	0.00010601
H	2.21975373	0.23731552	-0.00019290
H	-1.37332580	-2.29822307	-0.00004442
H	1.35355058	-2.30985148	0.00011093

$(S_1)_{min}$

C	1.13107501	-0.09165198	-0.00000139
C	0.00317413	0.75906431	0.00000192
C	-1.13194115	-0.08200160	0.00001051
C	0.68571035	-1.49508103	-0.00000750
C	-0.69856201	-1.48917795	0.00000506
C	0.00945770	2.23258033	0.00000349
H	-0.92941872	2.79022229	-0.00009229
H	0.95305580	2.78219484	0.00007889
H	-2.17149667	0.25469788	0.00002402
H	2.17346431	0.23616930	-0.00001596
H	-1.36335082	-2.35327354	0.00000995
H	1.34310541	-2.36481496	-0.00001672

$(S_0/S_1)_{MECI}$

C	1.12291553	-0.09979683	-0.15213896
C	0.00191781	0.75877175	-0.03393816
C	-1.13493616	-0.08110219	0.11481787
C	0.68845167	-1.50265060	-0.07369933
C	-0.68665164	-1.48643407	0.09078036
C	0.01036462	2.23354626	0.00194365
H	-0.42288449	2.82287964	-0.81248702
H	0.43625079	2.76840899	0.85703702
H	-2.16256604	0.26141874	0.25351949
H	2.15582261	0.22933095	-0.28745490
H	-1.34389273	-2.35161518	0.18987643
H	1.33948141	-2.37382955	-0.14825648

$(S_0/S_1)^{0^\circ}_{CI}$

C	1.07669592	-0.06755613	0.00449922
C	-0.02390924	0.74468479	0.00112812
C	-1.11361075	-0.09124491	0.00050663
C	0.65359033	-1.57670407	0.00621877
C	-0.66965288	-1.58533178	0.00172413
C	0.02653563	2.34515803	-0.00142982
H	-0.89782931	2.92151161	-0.00408593
H	0.99583130	2.84326723	-0.00047057
H	-2.16435731	0.21637068	0.00426366
H	2.11992884	0.26682179	-0.00090306
H	-1.35750672	-2.43059219	-0.00597875

H	1.35855752	-2.40745714	-0.00547239

$(S_0/S_1)^{10°}_{CI}$

C	1.07668212	-0.06900796	0.01023241
C	-0.02196635	0.74731820	0.00071265
C	-1.11258771	-0.08953346	-0.00442334
C	0.65388432	-1.57929961	0.01135484
C	-0.66870123	-1.58437283	-0.00512956
C	0.02485672	2.34570607	-0.00315829
H	-0.89992800	2.92115724	0.02321328
H	0.99383503	2.84322669	-0.03250464
H	-2.16421703	0.21602660	0.00818620
H	2.12090685	0.26375336	0.00695378
H	-1.35929565	-2.42745622	-0.01856344
H	1.36080426	-2.40859017	0.00312613

$(S_0/S_1)^{20°}_{CI}$

C	1.08090016	-0.06843512	0.03697626
C	-0.02109265	0.74979134	-0.00183336
C	-1.11227463	-0.08925592	-0.04235050
C	0.65443037	-1.57049883	0.01965393
C	-0.67214940	-1.57705746	-0.00789067
C	0.02522397	2.33255668	0.00184561
H	-0.85476873	2.90562221	0.29319670
H	0.94466260	2.84046062	-0.28951980
H	-2.16105820	0.21822013	-0.10222062
H	2.12320349	0.26511496	0.08185116
H	-1.35890393	-2.42329285	-0.01808009
H	1.35610028	-2.40429787	0.02837140

$(S_0/S_1)^{30°}_{CI}$

C	1.08806846	-0.06903258	0.05577599
C	-0.01834681	0.75276666	-0.00263051
C	-1.11186136	-0.08790167	-0.06276045
C	0.65508748	-1.56056019	0.03028184
C	-0.67587788	-1.56726338	-0.01335485
C	0.02349833	2.31636002	0.00189830
H	-0.80144902	2.88651119	0.42996009
H	0.88228652	2.83526648	-0.42539203
H	-2.15768334	0.22229293	-0.14961124
H	2.12889677	0.26463829	0.12162010
H	-1.36002195	-2.41546666	-0.03154994
H	1.35167613	-2.39868319	0.04576273

$(S_0/S_1)^{40°}_{CI}$

C	1.09970517	-0.07190131	0.07504909
C	-0.01288321	0.75615519	-0.00321246
C	-1.11031550	-0.08612296	-0.08223195
C	0.65681788	-1.54713883	0.04191113
C	-0.68079206	-1.55289441	-0.02163429
C	0.01911835	2.29582166	0.00075758
H	-0.73177382	2.86128814	0.55476726
H	0.79467370	2.82896014	-0.55174445
H	-2.15197909	0.22984657	-0.19192399
H	2.13878539	0.26089557	0.16145509
H	-1.36167874	-2.40342280	-0.04939558
H	1.34459527	-2.39255905	0.06620260

$(S_0/S_1)^{50°}_{CI}$

C	1.11305989	-0.07750635	0.09390407
C	-0.00632310	0.75932140	-0.00343497
C	-1.11005755	-0.08486546	-0.10032475
C	0.66069765	-1.53038186	0.05359025
C	-0.68669549	-1.53523123	-0.03526527
C	0.01381076	2.27305246	-0.00084972

H	-0.64411749	2.83511048	0.66562330
H	0.68612835	2.82079671	-0.66505535
H	-2.14712886	0.23849040	-0.22717306
H	2.15015370	0.25375866	0.19799909
H	-1.36262771	-2.38910383	-0.07487920
H	1.33737318	-2.38451347	0.09586563

$(S_0/S_1)_{CI}^{60^\circ}$

C	1.12562600	-0.08525539	0.11328081
C	-0.00087799	0.76098869	-0.00348333
C	-1.11334428	-0.08585434	-0.11874608
C	0.66809053	-1.51065321	0.06590710
C	-0.69250384	-1.51667537	-0.06127396
C	0.00917697	2.25181944	-0.00248338
H	-0.53779692	2.81528742	0.75828191
H	0.56399926	2.80781009	-0.76290947
H	-2.14703917	0.24506447	-0.24883439
H	2.15999964	0.24583393	0.23388067
H	-1.36223984	-2.37526051	-0.10768801
H	1.33118297	-2.37417731	0.13406816

$(S_0/S_1)_{CI}^{70^\circ}$

C	1.13484461	-0.09237490	0.13224194
C	0.00080290	0.75935001	-0.00350265
C	-1.12258434	-0.09017452	-0.13418021
C	0.67766009	-1.49124657	0.08141840
C	-0.69984832	-1.49738991	-0.07926794
C	0.00881606	2.23377211	-0.00585855
H	-0.41091625	2.79836006	0.83273163
H	0.43377777	2.79451740	-0.84448658
H	-2.15331931	0.24707122	-0.26656555
H	2.16532992	0.24250130	0.26814057
H	-1.35806217	-2.36289878	-0.15493169
H	1.32777235	-2.36255950	0.17426064

$(S_0/S_1)_{CI}^{80^\circ}$

C	1.13867925	-0.09852762	0.15731054
C	-0.00067604	0.75421148	-0.00209318
C	-1.13391042	-0.09710203	-0.15129042
C	0.68722215	-1.47756618	0.10359444
C	-0.70831505	-1.48070672	-0.08155541
C	0.01162985	2.22432350	-0.00851038
H	-0.27019039	2.79161752	0.88504434
H	0.30195974	2.78451775	-0.90408430
H	-2.16187356	0.24037574	-0.29597112
H	2.16467014	0.24554127	0.30340505
H	-1.35360455	-2.35254699	-0.19732280
H	1.32868219	-2.35520981	0.19147325

$(S_0/S_1)_{CI}^{90^\circ}$

C	1.13151000	-0.10494266	0.17708312
C	0.00068100	0.75161625	-0.00252828
C	-1.14131497	-0.09749735	-0.17810538
C	0.69843723	-1.48147375	0.11407295
C	-0.70190782	-1.46579098	-0.10568371
C	0.01118193	2.22328887	-0.00587385
H	-0.12990335	2.78944171	0.92141265
H	0.16025621	2.78368163	-0.93540573
H	-2.16556205	0.24237341	-0.33611487
H	2.15523032	0.24135171	0.33584214
H	-1.34711461	-2.34160917	-0.20057708
H	1.33277942	-2.36151176	0.21587805

$(S_0/S_1)_{CI}^{100^\circ}$

C	1.12538646	-0.10463423	0.20013915
C	0.00648019	0.75481608	-0.00327563
C	-1.13173855	-0.09103630	-0.20403122
C	0.69148903	-1.48613985	0.12740793
C	-0.69651997	-1.47320830	-0.10762182
C	0.00634774	2.22511344	-0.00857396
H	0.00382058	2.78877122	0.93059874
H	0.00876343	2.78856523	-0.94803565
H	-2.15196702	0.25916125	-0.37316261
H	2.15002238	0.22691323	0.37839527
H	-1.34169129	-2.34663821	-0.21158934
H	1.33388033	-2.36275565	0.21974915

$(S_0/S_1)_{CI}^{110^\circ}$

C	1.10977730	-0.09809323	0.21390815
C	0.00596221	0.75989201	-0.00292462
C	-1.12086933	-0.08444186	-0.22160016
C	0.68131044	-1.50233271	0.13244567
C	-0.68087650	-1.48683929	-0.12400073
C	0.00754721	2.23360697	-0.00490973
H	0.15433402	2.79666137	0.92220207
H	-0.13779347	2.79718404	-0.93205106
H	-2.14074959	0.25764282	-0.40962371
H	2.13437998	0.23195080	0.40068682
H	-1.33431313	-2.35376618	-0.23283619
H	1.32556417	-2.37253685	0.25870349

$(S_0/S_1)_{CI}^{120^\circ}$

C	1.09353751	-0.09231602	0.23156793
C	0.00520674	0.76258923	-0.00094961
C	-1.10817218	-0.07924441	-0.22936147
C	0.66902012	-1.52163031	0.15061174
C	-0.66667451	-1.50788424	-0.14316856
C	0.00745747	2.25282663	-0.00869328
H	0.30230128	2.81012500	0.88382399
H	-0.28335260	2.81506139	-0.89989250
H	-2.12902752	0.25870938	-0.42430349
H	2.11983831	0.23206038	0.42342486
H	-1.32387756	-2.36725695	-0.28367604
H	1.31801625	-2.38411218	0.30061643

$(S_0/S_1)_{CI}^{130^\circ}$

C	1.08935241	-0.09442293	0.24667911
C	0.01653514	0.75770514	0.00435580
C	-1.09106000	-0.06524368	-0.23891509
C	0.65983371	-1.54239759	0.15691612
C	-0.65654565	-1.52300624	-0.14499731
C	-0.00014331	2.27334864	-0.00551567
H	0.42821795	2.83707897	0.82734397
H	-0.44169215	2.81343597	-0.84756836
H	-2.10583846	0.27362779	-0.46556698
H	2.11541638	0.21988282	0.45770067
H	-1.32101693	-2.37106578	-0.31346097
H	1.31121422	-2.40001521	0.32302869

$(S_0/S_1)_{CI}^{140^\circ}$

C	1.08181599	-0.09476661	0.26865016
C	0.02042996	0.75560773	0.00635752
C	-1.07061600	-0.06284459	-0.25548742
C	0.64936864	-1.55832293	0.17010011
C	-0.64880568	-1.54212111	-0.15777258
C	-0.00487894	2.29597563	-0.00688156
H	0.54354643	2.86367498	0.74685695
H	-0.57363551	2.82594411	-0.77317716
H	-2.08427687	0.27905880	-0.48734331

H	2.10818552	0.21267198	0.49021001
H	-1.31784463	-2.38180014	-0.34654572
H	1.30098440	-2.41414994	0.34503299

$(S_0/S_1)_{CI}^{150°}$

C	1.07653288	-0.09617506	0.28592386
C	0.02398155	0.75246728	0.00810693
C	-1.05536443	-0.06092847	-0.26943218
C	0.64145340	-1.57190226	0.17905995
C	-0.64340075	-1.55617068	-0.16983307
C	-0.00789778	2.31660493	-0.00702351
H	0.65089699	2.88690790	0.64834862
H	-0.69381267	2.83510236	-0.67778674
H	-2.06534477	0.28055338	-0.51903295
H	2.09947480	0.20696562	0.52928016
H	-1.31595875	-2.38952971	-0.37267616
H	1.29371285	-2.42496737	0.36506509

$(S_0/S_1)_{CI}^{160°}$

C	1.07111833	-0.09706109	0.30393766
C	0.02591770	0.74936102	0.00874675
C	-1.04266225	-0.06084894	-0.28727744
C	0.63562803	-1.58185478	0.18727133
C	-0.63899793	-1.56598901	-0.18147077
C	-0.00954823	2.33293376	-0.00662352
H	0.74315712	2.90432088	0.53622235
H	-0.79441953	2.84242669	-0.56566173
H	-2.04822303	0.27997168	-0.55640085
H	2.08923505	0.20329077	0.57098040
H	-1.31572548	-2.39462206	-0.38882326
H	1.28879354	-2.43300100	0.37909907

$(S_0/S_1)_{CI}^{170°}$

C	1.06563165	-0.09742863	0.31960614
C	0.02668800	0.74749338	0.00821039
C	-1.03287967	-0.06120027	-0.30679001
C	0.63183307	-1.58750104	0.19243101
C	-0.63572558	-1.57168366	-0.19065741
C	-0.01062625	2.34311791	-0.00538813
H	0.81895211	2.91282275	0.41213808
H	-0.87455629	2.84849684	-0.43669201
H	-2.03221372	0.27874520	-0.59945820
H	2.07707536	0.20181886	0.61241149
H	-1.31373154	-2.39859668	-0.40034532
H	1.28382618	-2.43715676	0.39453398

$(S_0/S_1)_{CI}^{180°}$

C	1.05962765	-0.09619653	0.33269337
C	0.02541506	0.74681126	0.00907985
C	-1.03008786	-0.06074129	-0.31669625
C	0.62858104	-1.58814541	0.20278683
C	-0.63151109	-1.57483736	-0.20243707
C	-0.00896575	2.34442740	-0.00591370
H	0.86362804	2.91308893	0.31309090
H	-0.91478674	2.85038383	-0.33870626
H	-2.02670578	0.27796400	-0.62073954
H	2.06701488	0.20508460	0.63828000
H	-1.30694260	-2.40136701	-0.42204042
H	1.27900649	-2.43754453	0.41060230

6.2 6-methyl-Fulvene

6.2.1 CASSCF

$(S_0)_{min}$

C	1.04824486	-0.14812439	0.46472031
C	-0.09280139	0.75232061	0.21339590
C	-1.17531936	-0.11011575	-0.29936683
C	0.67517540	-1.41271755	0.13341677
C	-0.72382206	-1.38971691	-0.34747338
C	-0.17935513	2.08961873	0.39705606
H	-1.12043296	2.56614232	0.14721667
H	-2.15318593	0.24615503	-0.58112833
H	2.00863518	0.15315205	0.84550287
H	-1.27696937	-2.25502926	-0.67733469
H	1.28826029	-2.29766714	0.20435043
C	0.89901814	2.99820202	0.91569608
H	0.56222696	3.50723647	1.82086436
H	1.81403672	2.46089157	1.14701407
H	1.13128144	3.76929619	0.17857689

$(S_1)_{min}$

C	0.99093488	-0.12909160	0.44784659
C	-0.10624443	0.70930323	0.20191715
C	-1.13385789	-0.10149940	-0.28357643
C	0.62333447	-1.51111547	0.09963426
C	-0.66242203	-1.49675988	-0.34333085
C	-0.20878884	2.19320344	0.40368621
H	-1.14933563	2.65900655	0.15195250
H	-2.11862600	0.23350245	-0.57104744
H	1.95344869	0.16902929	0.82882252
H	-1.24831949	-2.33624756	-0.68041528
H	1.27465601	-2.36526443	0.18866854
C	0.90318014	3.05009483	0.92548593
H	0.61038618	3.56207353	1.84652128
H	1.79664414	2.46987530	1.14289424
H	1.18000255	3.82353370	0.20344794

$(S_0/S_1)_{MECI}$

C	1.09444710	-0.03157665	-0.06285310
C	-0.07035468	0.75226520	0.06748610
C	-1.16296767	-0.13690838	0.06452693
C	0.70867211	-1.43818421	-0.14780422
C	-0.65994742	-1.50232211	-0.06881188
C	-0.14223310	2.23056606	0.17229110
H	-0.49325338	2.77810826	-0.69389334
H	-2.20117802	0.14187370	0.15397671
H	2.10625047	0.33917812	-0.10019930
H	-1.26962540	-2.39082596	-0.09522902
H	1.39168314	-2.26551093	-0.25242494
C	0.68507003	2.96961380	1.18369599
H	0.36103889	4.00645296	1.27211333
H	0.61332631	2.49818575	2.16487278
H	1.74406439	2.97872840	0.90476005

$(S_0/S_1)_{CI}^{0^\circ}$

C	0.96729608	-0.12606878	0.43879472
C	-0.11578703	0.68883745	0.19537616
C	-1.12116462	-0.10301279	-0.27883818
C	0.60604776	-1.57218134	0.08214790
C	-0.64104806	-1.56218865	-0.34606394
C	-0.22410929	2.25916735	0.40975644
H	-1.16570965	2.71897264	0.15826591
H	-2.10942341	0.22373237	-0.56832197
H	1.93068064	0.17357288	0.81988002
H	-1.24287376	-2.38917500	-0.68661686
H	1.27709823	-2.41009327	0.17997379
C	0.90819678	3.08721691	0.93361472
H	0.63591177	3.60387523	1.85888179
H	1.78633094	2.48225067	1.14973733
H	1.21354636	3.85473834	0.21591936

$(S_0/S_1)_{CI}^{10^\circ}$

C	0.95166456	-0.13435396	0.48437925
C	-0.12215643	0.68538697	0.21703965
C	-1.11385391	-0.09919302	-0.29633067
C	0.59883877	-1.57520399	0.10092597
C	-0.62779752	-1.55474230	-0.38157620
C	-0.23551076	2.25277425	0.44478919
H	-1.19431926	2.70330813	0.24640547
H	-2.10167447	0.22794331	-0.58689235
H	1.91079802	0.16247295	0.87836374
H	-1.21994206	-2.37636284	-0.75086259
H	1.27110109	-2.41266341	0.19412574
C	0.91477723	3.09052868	0.91145432
H	0.68307794	3.62397584	1.83781863
H	1.80351784	2.49077749	1.09643068
H	1.18647169	3.84499591	0.16643637

$(S_0/S_1)_{CI}^{20^\circ}$

C	0.91680400	-0.15085023	0.57985724
C	-0.13871382	0.68122733	0.27258927
C	-1.09189905	-0.08404624	-0.34658852
C	0.59684870	-1.56870122	0.11045778
C	-0.60651206	-1.53308521	-0.43539813
C	-0.26111773	2.22253485	0.56246516
H	-1.25286687	2.64467629	0.56266759
H	-2.04089382	0.26363051	-0.72785541
H	1.83098889	0.12413293	1.08196367
H	-1.17159176	-2.34253384	-0.86840358
H	1.25875768	-2.41226058	0.22108118
C	0.92814454	3.09364745	0.83029766
H	0.79683456	3.65933362	1.75613432
H	1.84049634	2.50959197	0.93256998
H	1.09971313	3.82234638	0.03066900

$(S_0/S_1)_{CI}^{30^\circ}$

C	0.93263793	-0.13799704	0.54965692
C	-0.16069405	0.68979627	0.34316087
C	-1.11939487	-0.05754578	-0.30271696
C	0.63130673	-1.51146596	-0.01258006
C	-0.59860245	-1.46872622	-0.51533731
C	-0.29214189	2.17459933	0.75540362
H	-1.27151814	2.55445877	0.99307503
H	-2.09001225	0.29063239	-0.62346617
H	1.85835450	0.13193526	1.03324892
H	-1.15430866	-2.26297498	-0.98689193
H	1.31592358	-2.34390331	0.00346615
C	0.91803623	3.04751412	0.81778651
H	0.64698269	4.09190570	0.96774311
H	1.57900516	2.75627047	1.64086790
H	1.50941821	2.97514498	-0.10090939

$(S_0/S_1)_{CI}^{40^\circ}$

C	0.91645410	-0.17189496	0.62388910
C	-0.15320723	0.68425872	0.38192944
C	-1.09232830	-0.03709604	-0.33768803
C	0.62249304	-1.51553842	0.01374669
C	-0.58705218	-1.43947967	-0.55248000
C	-0.27707257	2.13925551	0.82074920
H	-1.23184949	2.49194735	1.17501136
H	-2.03535175	0.33961443	-0.70425536
H	1.81561922	0.06742688	1.16966317
H	-1.12212573	-2.21407367	-1.07750819
H	1.28469668	-2.36535387	0.04904647
C	0.90292765	3.05837733	0.76066214
H	0.61180442	4.08972821	0.95613832

H	1.65965634	2.78371355	1.50224996
H	1.39032851	3.01875866	-0.21864703

$(S_0/S_1)_{CI}^{50°}$

C	0.90161659	-0.21192841	0.69615579
C	-0.14043185	0.67679543	0.42017182
C	-1.06238502	-0.01133706	-0.37119188
C	0.61185173	-1.51759869	0.04314206
C	-0.57256078	-1.39928742	-0.59237072
C	-0.25346527	2.10313499	0.88379746
H	-1.17186839	2.42411667	1.35009388
H	-1.98092794	0.39194555	-0.76912298
H	1.77546666	-0.00219933	1.29262635
H	-1.09091193	-2.15392321	-1.16139123
H	1.25303858	-2.38322414	0.08242610
C	0.88016024	3.06612066	0.70636450
H	0.56748124	4.08922293	0.91273562
H	1.70996568	2.83200061	1.38041071
H	1.27796317	3.02580544	-0.31134023

$(S_0/S_1)_{CI}^{60°}$

C	0.88254808	-0.25495789	0.77351120
C	-0.12510314	0.66974577	0.45477451
C	-1.02232834	0.01399431	-0.40965541
C	0.60081344	-1.52210644	0.08548909
C	-0.55275459	-1.35970847	-0.62645977
C	-0.22753392	2.07084638	0.93547173
H	-1.09925533	2.36357262	1.50121310
H	-1.90611695	0.45016305	-0.84844365
H	1.72212355	-0.07633622	1.42634421
H	-1.04202865	-2.09421191	-1.24512758
H	1.20890193	-2.41033378	0.14469750
C	0.85044645	3.07456535	0.64978793
H	0.54840911	4.07422045	0.95974072
H	1.77612216	2.82743642	1.17745865
H	1.09074890	3.10275437	-0.41629497

$(S_0/S_1)_{CI}^{70°}$

C	0.87231078	-0.29316772	0.81493027
C	-0.11124190	0.66020673	0.48017084
C	-0.99580221	0.02944525	-0.42389602
C	0.58980885	-1.53027391	0.10656485
C	-0.53897401	-1.32744212	-0.65743097
C	-0.20523881	2.05554449	0.97663627
H	-1.03791636	2.32836693	1.60961016
H	-1.86524173	0.48654480	-0.86954808
H	1.69889733	-0.13271031	1.48870224
H	-1.01092344	-2.04592642	-1.30758388
H	1.17018557	-2.43647158	0.16762363
C	0.82841155	3.08429772	0.61679961
H	0.50761285	4.08339944	0.90994896
H	1.78498864	2.88296299	1.10878234
H	1.01811557	3.08486770	-0.45880296

$(S_0/S_1)_{CI}^{80°}$

C	0.83326966	-0.35047980	0.92527575
C	-0.08681983	0.64890844	0.51309213
C	-0.91104677	0.06513784	-0.48936974
C	0.57612224	-1.54594255	0.17867989
C	-0.49290934	-1.28719191	-0.69520630
C	-0.17061693	2.02925970	1.02755774
H	-0.92473670	2.26767863	1.76422113
H	-1.71418935	0.56406443	-1.00795145
H	1.59429199	-0.22932945	1.67946874
H	-0.92050362	-1.97827573	-1.40331032

H	1.10799617	-2.47807900	0.27922212
C	0.76766718	3.09888546	0.54756705
H	0.62487745	4.02801408	1.09777987
H	1.81051025	2.79134624	0.66035562
H	0.61108028	3.30564761	-0.51487497

$(S_0/S_1)^{90^\circ}_{CI}$

C	0.80774386	-0.39906183	0.98055974
C	-0.06058110	0.63830170	0.53805417
C	-0.85040547	0.09851240	-0.51618773
C	0.55378262	-1.56672612	0.20480482
C	-0.47098395	-1.25941410	-0.71955782
C	-0.12657328	2.01413475	1.07427775
H	-0.80054010	2.22394011	1.89269613
H	-1.61148380	0.63147193	-1.06329663
H	1.53418511	-0.31094928	1.77240115
H	-0.89173332	-1.93160438	-1.44960619
H	1.05761009	-2.51443793	0.30411429
C	0.72201234	3.11351468	0.50267947
H	0.59969112	4.03952865	1.06295600
H	1.78054779	2.84148096	0.51962150
H	0.46172076	3.31095246	-0.54100940

$(S_0/S_1)^{100^\circ}_{CI}$

C	0.78175175	-0.44362791	1.02597157
C	-0.02744804	0.63161308	0.56266125
C	-0.78514739	0.13655538	-0.53177641
C	0.52078411	-1.59451454	0.21779305
C	-0.44800282	-1.24037780	-0.73657361
C	-0.06997253	1.99903201	1.11493500
H	-0.65007860	2.18298802	2.00808659
H	-1.50365658	0.70137059	-1.10451809
H	1.47654379	-0.39382876	1.84906258
H	-0.86997659	-1.88574053	-1.48950398
H	0.99381791	-2.55767888	0.32168451
C	0.67246175	3.12982874	0.46307908
H	0.52741380	4.06300418	1.00560445
H	1.74475137	2.91913267	0.42423876
H	0.34175072	3.28188774	-0.56823751

$(S_0/S_1)^{110^\circ}_{CI}$

C	0.75068307	-0.47997762	1.06824123
C	0.00725319	0.62627847	0.58575783
C	-0.71441340	0.17408338	-0.54097840
C	0.48075687	-1.62471308	0.22591109
C	-0.41612768	-1.22912004	-0.75275072
C	-0.00975928	1.98934923	1.15185277
H	-0.48671838	2.15142438	2.10774040
H	-1.38633831	0.76889578	-1.13893241
H	1.41075528	-0.46858978	1.92122341
H	-0.82877594	-1.84468371	-1.53551998
H	0.91299311	-2.60494953	0.34479488
C	0.61918060	3.14416242	0.42778257
H	0.46937411	4.07894645	0.96636137
H	1.69496993	2.98896932	0.30671824
H	0.20115949	3.25956833	-0.57569505

$(S_0/S_1)^{120^\circ}_{CI}$

C	0.72991820	-0.50428173	1.09741173
C	0.03482530	0.62162616	0.60350456
C	-0.66644977	0.20249167	-0.54115554
C	0.44680099	-1.64811295	0.23020257
C	-0.39176902	-1.22082659	-0.76588301
C	0.03661406	1.98375801	1.17214450
H	-0.36593261	2.13619586	2.16221409

H	-1.31323961	0.81480333	-1.14920591
H	1.37421295	-0.52096375	1.96268099
H	-0.79964251	-1.80854451	-1.57230001
H	0.84951548	-2.63918713	0.36250688
C	0.58299895	3.15129246	0.40623341
H	0.40347388	4.08970634	0.92947238
H	1.66122594	3.05020031	0.24678146
H	0.12244044	3.22148651	-0.58210088

$(S_0/S_1)_{CI}^{130^\circ}$

C	0.67806556	-0.53987781	1.13886188
C	0.08085816	0.60870169	0.61395258
C	-0.56379195	0.22928122	-0.56320764
C	0.38936757	-1.69223499	0.24124784
C	-0.35131410	-1.22957055	-0.78605007
C	0.12610073	1.99153843	1.20793863
H	-0.12535193	2.11336437	2.25000987
H	-1.13748747	0.87237482	-1.21154111
H	1.26910556	-0.59091317	2.04032997
H	-0.73020864	-1.78610168	-1.62797665
H	0.72369252	-2.70335563	0.40827296
C	0.51030111	3.17992705	0.38039857
H	0.37071959	4.10606470	0.93663231
H	1.55727328	3.13012323	0.06218159
H	-0.09233732	3.24032231	-0.52854349

$(S_0/S_1)_{CI}^{140^\circ}$

C	0.62034374	-0.55579929	1.18528517
C	0.10997281	0.60152937	0.61987092
C	-0.45622857	0.24392114	-0.59942328
C	0.35807905	-1.71610514	0.26177868
C	-0.27807414	-1.23389063	-0.81111190
C	0.16382426	1.99986739	1.22788805
H	-0.00122188	2.10727052	2.28753198
H	-0.94965398	0.90606100	-1.29349363
H	1.13792109	-0.62661257	2.13008384
H	-0.60900654	-1.77415402	-1.68321266
H	0.64025727	-2.73714108	0.46128484
C	0.43980306	3.19333440	0.36964542
H	0.62262997	4.08270369	0.97180042
H	1.31028693	3.02767957	-0.27255156
H	-0.40394041	3.41097964	-0.29286905

$(S_0/S_1)_{CI}^{150^\circ}$

C	0.58194485	-0.57389084	1.21100663
C	0.14957527	0.59446969	0.62375458
C	-0.37205100	0.25889250	-0.61709719
C	0.31457607	-1.74087705	0.27149518
C	-0.24321278	-1.23679320	-0.82910076
C	0.23524802	2.00908522	1.24227390
H	0.18811231	2.10426144	2.31252058
H	-0.80426359	0.93893175	-1.33499796
H	1.05035332	-0.66518632	2.18019577
H	-0.56327315	-1.76081193	-1.71510459
H	0.53987054	-2.77158460	0.49290255
C	0.37996437	3.20610399	0.36130593
H	0.61650848	4.09851682	0.93960907
H	1.17230979	3.05964752	-0.38050801
H	-0.54066983	3.40887899	-0.19574845

$(S_0/S_1)_{CI}^{160^\circ}$

C	0.54526614	-0.58716776	1.22776208
C	0.19015636	0.58788305	0.61953594
C	-0.29578654	0.26878251	-0.63706792
C	0.26333938	-1.75446700	0.28247886

C	-0.23044138	-1.23849643	-0.83304045
C	0.31745610	2.01499535	1.24452750
H	0.39670233	2.09563878	2.31499845
H	-0.66322287	0.96149124	-1.37794040
H	0.97049411	-0.69213836	2.21512870
H	-0.53702535	-1.75292645	-1.72927526
H	0.44462563	-2.79060186	0.51833992
C	0.32644405	3.21612607	0.36041174
H	0.54082593	4.12215542	0.92567308
H	1.07625765	3.12262687	-0.43265281
H	-0.64009888	3.35574255	-0.13637222

$(S_0/S_1)_{CI}^{170^\circ}$

C	0.50980531	-0.59482236	1.24299857
C	0.23219496	0.58438399	0.61273787
C	-0.21534070	0.27606061	-0.65932111
C	0.21560342	-1.76343474	0.29483790
C	-0.21373746	-1.23843336	-0.83976277
C	0.39826537	2.01884718	1.23773781
H	0.60136942	2.09210395	2.29192481
H	-0.52091590	0.97659546	-1.42055816
H	0.88387156	-0.70886937	2.25006149
H	-0.51090259	-1.74653985	-1.74271730
H	0.34682108	-2.80331639	0.54666567
C	0.27173587	3.22085422	0.36591357
H	0.47725027	4.13492710	0.92119146
H	0.96459123	3.17524586	-0.48176917
H	-0.73561918	3.30604169	-0.05743344

$(S_0/S_1)_{CI}^{180^\circ}$

C	0.47495977	-0.59730353	1.25547549
C	0.27426141	0.58320143	0.60201282
C	-0.13636634	0.27814638	-0.68287168
C	0.16695978	-1.76630482	0.30972612
C	-0.19813581	-1.23869756	-0.84489197
C	0.47904898	2.02000029	1.21820601
H	0.80493809	2.09029945	2.24122716
H	-0.37776033	0.98099502	-1.46470866
H	0.79919813	-0.71437338	2.27933208
H	-0.48952412	-1.74451436	-1.75100743
H	0.24617956	-2.80740153	0.57795443
C	0.21915762	3.22280382	0.37825487
H	0.40404057	4.14044773	0.93477065
H	0.85548364	3.23488518	-0.51393139
H	-0.81744829	3.24745986	0.02295871

6.2.2 XMS-CASPT2

$(S_0)_{min}$

C	1.05323809	-0.12927285	0.46953182
C	-0.10118307	0.75268577	0.21075205
C	-1.18785466	-0.10557456	-0.30280112
C	0.67923444	-1.41232693	0.13480399
C	-0.71932174	-1.39906203	-0.34743975
C	-0.19144733	2.10272046	0.39491301
H	-1.14369031	2.58642525	0.14225200
H	-2.17710030	0.25629937	-0.58757908
H	2.02179705	0.18489345	0.85527499
H	-1.27264564	-2.27970117	-0.67993239
H	1.30418699	-2.30497049	0.20849597
C	0.90336175	2.98878239	0.91562511
H	0.57491550	3.50362937	1.83395357
H	1.81162086	2.41688954	1.13928281
H	1.14988114	3.76822643	0.17537419

$(S_1)_{min}$

C	0.99248099	-0.11038532	0.45136084
C	-0.11829472	0.73543193	0.20181688
C	-1.14510356	-0.10020225	-0.28729954
C	0.63996203	-1.49722412	0.10769536
C	-0.66734722	-1.49354272	-0.34446693
C	-0.22133024	2.19204652	0.39890532
H	-1.17150944	2.67223566	0.14639946
H	-2.14436425	0.23307354	-0.58011306
H	1.96509500	0.19866925	0.83755488
H	-1.25451679	-2.34743036	-0.68425039
H	1.30609639	-2.35560010	0.20123729
C	0.90520830	3.02797416	0.92264345
H	0.62530465	3.54655595	1.85820238
H	1.79321740	2.41703984	1.13299522
H	1.20009423	3.81100203	0.19982604

$(S_0/S_1)_{MECI}$

C	0.70752258	-0.22114399	1.02964081
C	-0.16790858	0.71109553	0.42431680
C	-0.88135667	-0.00134817	-0.57774753
C	0.52760745	-1.54308659	0.39302862
C	-0.43757143	-1.40631082	-0.58596855
C	-0.29094424	2.14176294	0.75747439
H	-1.17900621	2.50041870	1.28804206
H	-1.63325741	0.42974012	-1.24287496
H	1.39339941	-0.00535152	1.85192040
H	-0.80999015	-2.18000718	-1.25859603
H	1.06917730	-2.45011257	0.66491629
C	0.86424355	3.06510872	0.50468784
H	1.70802925	2.83860336	1.18621748
H	1.24837283	2.93079918	-0.52213393
H	0.58667509	4.11947633	0.64958349

$(S_0/S_1)_{CI}^{0^\circ}$

C	0.94101857	-0.10945825	0.43352261
C	-0.13951401	0.71228985	0.19097439
C	-1.11923185	-0.11036582	-0.27976232
C	0.61641956	-1.59809953	0.08300052
C	-0.63208841	-1.60562932	-0.34842043
C	-0.25359446	2.31522338	0.40793626
H	-1.20447203	2.78379126	0.15376253
H	-2.12658736	0.19887964	-0.58014351
H	1.91662010	0.20077878	0.81881635
H	-1.24846639	-2.43481936	-0.69567995
H	1.32281015	-2.42038012	0.19446009
C	0.91297863	3.09033745	0.93574535
H	0.68384624	3.60767242	1.88740113
H	1.77208793	2.43296515	1.13293277
H	1.26316609	3.86645847	0.22796140

$(S_0/S_1)_{CI}^{10^\circ}$

C	0.86102155	-0.06792073	0.61474981
C	-0.18080624	0.74879845	0.19369060
C	-1.08705042	-0.08519276	-0.38627293
C	0.58149889	-1.55521963	0.26566230
C	-0.59882921	-1.57757932	-0.33517617
C	-0.28723252	2.33632944	0.35879962
H	-1.22985649	2.83009848	0.13086546
H	-2.03744366	0.21554153	-0.84076701
H	1.77146901	0.26462798	1.12130328
H	-1.16465034	-2.42056240	-0.73203112
H	1.26064839	-2.37677959	0.49245301
C	0.94435135	3.02468952	0.82274569
H	1.23766853	2.69281685	1.84209995

H	1.80976330	2.78231644	0.16870065
H	0.82444064	4.11767972	0.84568405

$(S_0/S_1)_{CI}^{20^\circ}$

C	0.82883141	-0.08594795	0.68895295
C	-0.18851180	0.74291353	0.23519811
C	-1.06386958	-0.07573358	-0.42092826
C	0.57045988	-1.55913066	0.28167470
C	-0.58098237	-1.56401663	-0.37779690
C	-0.30386565	2.31411780	0.43997554
H	-1.27619061	2.79206201	0.33329202
H	-1.98329074	0.24053517	-0.92535361
H	1.71068293	0.23227157	1.25227768
H	-1.12598678	-2.39794112	-0.82035285
H	1.23891798	-2.38765734	0.51506732
C	0.95499496	3.03343116	0.77020659
H	1.33868479	2.74603613	1.77228144
H	1.76255051	2.77327791	0.05317263
H	0.82256785	4.12542597	0.76483984

$(S_0/S_1)_{CI}^{30^\circ}$

C	0.79967995	-0.11079442	0.76373814
C	-0.18839461	0.73704792	0.27860884
C	-1.03368970	-0.06208984	-0.45199915
C	0.55568416	-1.56369318	0.29983282
C	-0.56361887	-1.54387226	-0.41986163
C	-0.30921750	2.28366421	0.51799168
H	-1.29681956	2.74343219	0.53331846
H	-1.91718334	0.27728969	-1.00265630
H	1.64856835	0.18506952	1.38672948
H	-1.08786546	-2.36615582	-0.90709627
H	1.20652066	-2.40432751	0.54012143
C	0.95742344	3.04404687	0.71348149
H	1.43578135	2.79414020	1.68312627
H	1.70171331	2.78365081	-0.06753659
H	0.79641062	4.13223559	0.69470854

$(S_0/S_1)_{CI}^{40^\circ}$

C	0.77160917	-0.13873370	0.83921283
C	-0.18336745	0.73123401	0.32167419
C	-1.00049124	-0.04473714	-0.48184226
C	0.53852159	-1.56643442	0.32381545
C	-0.54591816	-1.51617093	-0.45749690
C	-0.30343752	2.24811273	0.59051494
H	-1.29177884	2.68856566	0.72689568
H	-1.84614065	0.32097551	-1.07261658
H	1.58201338	0.13233057	1.52163581
H	-1.04289384	-2.32476807	-0.99385426
H	1.16663293	-2.42273065	0.57014496
C	0.95294135	3.05290966	0.65241044
H	1.52654439	2.82945287	1.57477108
H	1.62548453	2.80426609	-0.19307717
H	0.75527317	4.13537177	0.64031900

$(S_0/S_1)_{CI}^{50^\circ}$

C	0.74137503	-0.16421914	0.91555697
C	-0.17721470	0.72700946	0.36005519
C	-0.96197310	-0.02653214	-0.51633584
C	0.52124542	-1.56121028	0.35525207
C	-0.52494519	-1.48240637	-0.49078668
C	-0.29150734	2.21182196	0.65536510
H	-1.26513212	2.63573011	0.90896597
H	-1.77057096	0.36325219	-1.14153702
H	1.50327008	0.08366786	1.65940684
H	-0.98577687	-2.27823259	-1.07636439

H	1.11985290	-2.43476624	0.61476897
C	0.94218225	3.05488716	0.58941613
H	1.62527709	2.82202551	1.43036996
H	1.51472326	2.84823729	-0.33584649
H	0.71418705	4.13037919	0.63422043

$(S_0/S_1)_{CI}^{60^\circ}$

C	0.71826613	-0.20312854	0.98603874
C	-0.16349646	0.72060845	0.40225036
C	-0.92123894	-0.00329555	-0.54194805
C	0.50239607	-1.55819493	0.38208954
C	-0.50229665	-1.44050029	-0.53244403
C	-0.26989800	2.17360251	0.72294885
H	-1.21464795	2.57593584	1.09580596
H	-1.69365900	0.42027455	-1.18979895
H	1.43882407	0.01642371	1.77786439
H	-0.93059147	-2.22297955	-1.15918259
H	1.06277664	-2.45558686	0.64756772
C	0.92198667	3.06033339	0.52861529
H	1.67959212	2.87559199	1.31536489
H	1.41801021	2.84217342	-0.43545525
H	0.65896935	4.12838584	0.56279034

$(S_0/S_1)_{CI}^{70^\circ}$

C	0.67800387	-0.23804082	1.07507535
C	-0.15087477	0.71480818	0.43989135
C	-0.86248108	0.01482592	-0.57643464
C	0.47717048	-1.55718721	0.44023525
C	-0.45926421	-1.39902869	-0.56558009
C	-0.25158329	2.14637337	0.77098027
H	-1.16262254	2.53211709	1.23908256
H	-1.58708100	0.46286728	-1.26036281
H	1.34457116	-0.03729658	1.91674646
H	-0.83343423	-2.16611706	-1.24475740
H	0.98507703	-2.47796821	0.73047159
C	0.89611527	3.06242124	0.46325246
H	1.79335710	2.77811124	1.04612282
H	1.18075850	2.98222857	-0.60227113
H	0.65728048	4.11152966	0.69005517

$(S_0/S_1)_{CI}^{80^\circ}$

C	0.63321636	-0.27758842	1.15872304
C	-0.13564922	0.70519515	0.47280093
C	-0.79142466	0.03403713	-0.61007031
C	0.45187445	-1.56079867	0.49725115
C	-0.41347024	-1.36485958	-0.59210573
C	-0.23179871	2.12820913	0.81583870
H	-1.10040675	2.49647373	1.37196623
H	-1.45950105	0.50929315	-1.33119224
H	1.24276024	-0.09529311	2.04593819
H	-0.73393769	-2.11893441	-1.31237730
H	0.90646052	-2.50323275	0.80645287
C	0.86288935	3.06839526	0.40093180
H	1.84881203	2.68747665	0.72546297
H	0.90636038	3.14458054	-0.70221296
H	0.71880778	4.07669019	0.81509988

$(S_0/S_1)_{CI}^{90^\circ}$

C	0.59698204	-0.32498080	1.21954378
C	-0.11206415	0.69394625	0.50556552
C	-0.71971342	0.06573806	-0.62950620
C	0.42543824	-1.57650802	0.52478726
C	-0.38631617	-1.33682899	-0.61184868
C	-0.19963028	2.11535227	0.86885356
H	-1.01044825	2.46033583	1.51934271

H	-1.33595123	0.57276410	-1.37426527
H	1.16152009	-0.17189708	2.14106756
H	-0.69724210	-2.07580525	-1.35182960
H	0.85018343	-2.53561340	0.82503475
C	0.82551204	3.07955060	0.34289150
H	1.84591708	2.71783421	0.56534444
H	0.75880220	3.16318020	-0.75850993
H	0.70200325	4.08257600	0.77603580

$(S_0/S_1)_{CI}^{100^\circ}$

C	0.56620609	-0.37107558	1.25422358
C	-0.08141740	0.69003068	0.54148633
C	-0.65572431	0.10963815	-0.62491975
C	0.38688223	-1.60721534	0.51949870
C	-0.37200721	-1.31768370	-0.62690564
C	-0.14561966	2.10304332	0.93017901
H	-0.87280080	2.42751919	1.68205582
H	-1.23462305	0.64812827	-1.37781269
H	1.10681687	-0.25664611	2.19596926
H	-0.69918493	-2.02794068	-1.38779431
H	0.79083113	-2.57988158	0.80381605
C	0.78652817	3.09308280	0.29371572
H	1.83305013	2.87411828	0.57926328
H	0.74261940	3.01802728	-0.80845697
H	0.55343612	4.12649900	0.58818883

$(S_0/S_1)_{CI}^{110^\circ}$

C	0.52221436	-0.40459650	1.29174425
C	-0.05109185	0.68440583	0.57349484
C	-0.58025526	0.14207010	-0.61695611
C	0.33963169	-1.64539161	0.52181919
C	-0.33242073	-1.31731832	-0.63992586
C	-0.09119170	2.10118517	0.98024113
H	-0.72921589	2.40704431	1.81521129
H	-1.11018693	0.70422410	-1.38931386
H	1.02615590	-0.32621565	2.25808383
H	-0.63610355	-1.99316891	-1.44060707
H	0.69195033	-2.63228714	0.82415086
C	0.74173149	3.11132417	0.24748860
H	1.82033282	2.95265316	0.44589677
H	0.60828329	3.00429209	-0.84381721
H	0.48515881	4.14142319	0.53499657

$(S_0/S_1)_{CI}^{120^\circ}$

C	0.45024017	-0.41626169	1.34349751
C	-0.02877212	0.67983020	0.59513200
C	-0.46805440	0.15263394	-0.62993615
C	0.30170055	-1.67523587	0.54928155
C	-0.24370607	-1.33228335	-0.65208019
C	-0.06510506	2.11105197	1.01234548
H	-0.63565268	2.40118047	1.89883538
H	-0.92336919	0.72431783	-1.44228241
H	0.87891901	-0.35746815	2.34727939
H	-0.47977273	-1.98630374	-1.49233548
H	0.59126684	-2.66874020	0.89364992
C	0.68289370	3.12887833	0.20718448
H	1.71766119	2.79018537	0.01170663
H	0.21065391	3.26884314	-0.78504739
H	0.71608965	4.10901575	0.70527650

$(S_0/S_1)_{CI}^{130^\circ}$

C	0.40127969	-0.43752258	1.37227324
C	0.00436877	0.67077235	0.62058338
C	-0.38045723	0.16919800	-0.62438670
C	0.25711948	-1.70863804	0.55114822

C	-0.19570179	-1.33978954	-0.66374131
C	-0.00982705	2.12487991	1.05692260
H	-0.48209655	2.39856313	2.00249994
H	-0.77030936	0.75999057	-1.45764364
H	0.77797028	-0.40697671	2.39878383
H	-0.40302766	-1.96819099	-1.53021836
H	0.48449221	-2.71139766	0.91405074
C	0.62910191	3.14619933	0.17025755
H	1.62609235	2.80572360	-0.17128615
H	0.02782037	3.30652040	-0.74677328
H	0.73816735	4.12031223	0.67003717

$(S_0/S_1)_{CI}^{140^\circ}$

C	0.34961143	-0.45114843	1.39757107
C	0.03463271	0.66279788	0.63751699
C	-0.30210234	0.17897343	-0.62309372
C	0.20143877	-1.73548972	0.55615421
C	-0.15983310	-1.34935621	-0.67345480
C	0.05338478	2.13769446	1.09514895
H	-0.30965850	2.39319912	2.09104897
H	-0.61976050	0.78292066	-1.47765194
H	0.67625333	-0.44180204	2.44239697
H	-0.31130675	-1.95446617	-1.56777043
H	0.38725488	-2.74108827	0.93328763
C	0.57423392	3.16105616	0.14184632
H	1.51130175	2.81537352	-0.33890000
H	-0.14292850	3.34302167	-0.68432336
H	0.76247089	4.12795794	0.63273036

$(S_0/S_1)_{CI}^{150^\circ}$

C	0.30975444	-0.46786594	1.41228597
C	0.07702080	0.65529197	0.65630011
C	-0.20954062	0.19171303	-0.62185831
C	0.16132445	-1.75566448	0.55317450
C	-0.12983422	-1.34922269	-0.68035808
C	0.12342253	2.15105686	1.13050441
H	-0.12814228	2.39056603	2.16300918
H	-0.45633820	0.81270270	-1.48777139
H	0.58282826	-0.47940045	2.47270483
H	-0.28839818	-1.94035202	-1.58229629
H	0.28502471	-2.76861552	0.93654566
C	0.51654508	3.17211839	0.11924247
H	1.42498468	2.85319442	-0.43298516
H	-0.27264746	3.30836547	-0.64915381
H	0.70898878	4.15575622	0.57316313

$(S_0/S_1)_{CI}^{160^\circ}$

C	0.27062330	-0.47755391	1.42567329
C	0.11948932	0.64917079	0.66800033
C	-0.12247962	0.19971086	-0.62373407
C	0.11372184	-1.76721318	0.55320003
C	-0.10432697	-1.34860302	-0.68701887
C	0.20181716	2.15936639	1.15489176
H	0.06978196	2.38360100	2.21203034
H	-0.29729000	0.83156915	-1.49929800
H	0.48275660	-0.50443878	2.50000652
H	-0.25213970	-1.92812817	-1.59804616
H	0.18312248	-2.78269484	0.94339589
C	0.45948190	3.17856041	0.10373973
H	1.32850250	2.88976185	-0.52469790
H	-0.39971897	3.27191163	-0.59463442
H	0.65165098	4.17462382	0.52899875

$(S_0/S_1)_{CI}^{170^\circ}$

C	0.23175933	-0.47496868	1.41986204

C	0.16066467	0.64230985	0.65294568
C	-0.04308044	0.18670144	-0.64700010
C	0.06237654	-1.77991287	0.56654364
C	-0.09490129	-1.37168352	-0.68695459
C	0.28696816	2.15279593	1.15732994
H	0.28136582	2.35278226	2.22037864
H	-0.13866269	0.81871319	-1.54039643
H	0.38741166	-0.48319351	2.50822126
H	-0.22285764	-1.95183086	-1.59977251
H	0.07756179	-2.79342736	0.96778654
C	0.40617725	3.20379175	0.11026980
H	1.25097739	2.97885622	-0.59442390
H	-0.50517577	3.24356103	-0.51342807
H	0.56440799	4.20514913	0.54114527

$(S_0/S_1)_{CI}^{180^\circ}$

C	0.19416295	-0.48204846	1.44096906
C	0.19905409	0.64133179	0.67384328
C	0.03842558	0.20004628	-0.63312093
C	0.01438626	-1.77223021	0.55710209
C	-0.05018274	-1.34907069	-0.69513638
C	0.35652883	2.15853149	1.16664339
H	0.45253308	2.36093827	2.23224442
H	-0.02694301	0.83505578	-1.52047136
H	0.30802313	-0.51681508	2.52963877
H	-0.18236853	-1.92226357	-1.61272730
H	-0.01963636	-2.78563185	0.95711155
C	0.35805979	3.18836098	0.09648803
H	1.14706369	2.98471981	-0.65831344
H	-0.60105064	3.18799845	-0.46692978
H	0.51693665	4.20072101	0.49516580

6.2.3 MS-CASPT2

$(S_0)_{min}$

C	1.05295824	-0.12898519	0.46945133
C	-0.10083563	0.75268060	0.21071886
C	-1.18683789	-0.10490157	-0.30249939
C	0.67918196	-1.41270019	0.13479044
C	-0.71929850	-1.39931282	-0.34745944
C	-0.19192572	2.10243620	0.39476691
H	-1.14399362	2.58564832	0.14215355
H	-2.17581376	0.25647865	-0.58726484
H	2.02135456	0.18457027	0.85494896
H	-1.27346383	-2.27907230	-0.68000682
H	1.30436593	-2.30488036	0.20872064
C	0.90290918	2.98873199	0.91552598
H	0.57518493	3.50361647	1.83401650
H	1.81131894	2.41716787	1.13913257
H	1.14988798	3.76816606	0.17551192

$(S_1)_{min}$

C	0.99135267	-0.10994864	0.45103649
C	-0.11820915	0.73378275	0.20160445
C	-1.14516005	-0.10083291	-0.28741182
C	0.63975648	-1.49785459	0.10749732
C	-0.66728376	-1.49442055	-0.34459789
C	-0.22098447	2.19308494	0.39923367
H	-1.17083634	2.67326742	0.14681860
H	-2.14417896	0.23304404	-0.58002586
H	1.96359789	0.20013364	0.83726733
H	-1.25382749	-2.34854195	-0.68420507
H	1.30583627	-2.35596155	0.20104459
C	0.90558816	3.02826671	0.92282761
H	0.62556249	3.54706534	1.85821062

H	1.79340370	2.41725497	1.13320600
H	1.20037533	3.81130440	0.20000117

$(S_0/S_1)_{MECI}$

C	0.70927782	-0.22052555	1.02845786
C	-0.16758617	0.71081540	0.42275756
C	-0.88262679	-0.00179464	-0.57791825
C	0.52866271	-1.54213224	0.39372653
C	-0.43874663	-1.40547144	-0.58564550
C	-0.29102395	2.14134523	0.75595671
H	-1.18054861	2.49956835	1.28415046
H	-1.63505842	0.42949836	-1.24227140
H	1.39519954	-0.00322375	1.85020822
H	-0.81102458	-2.18017943	-1.25678532
H	1.06937131	-2.44951045	0.66519763
C	0.86463619	3.06457681	0.50572224
H	1.70727558	2.83673365	1.18816911
H	1.25007012	2.93116847	-0.52067494
H	0.58711464	4.11877527	0.65145628

$(S_0/S_1)_{CI}^{0^\circ}$

C	0.94415714	-0.11021763	0.43352676
C	-0.13741676	0.70945649	0.19230723
C	-1.12046282	-0.10920312	-0.27692603
C	0.61649247	-1.59406254	0.08352304
C	-0.63432608	-1.60077738	-0.34457574
C	-0.25103620	2.30858694	0.40885879
H	-1.20250566	2.77724725	0.15630170
H	-2.12548870	0.20427078	-0.58100860
H	1.91839704	0.20038028	0.82266123
H	-1.24658857	-2.42911135	-0.70043143
H	1.32016810	-2.41845047	0.19405284
C	0.91284961	3.08774752	0.93401974
H	0.67959540	3.60636107	1.88402336
H	1.77363776	2.43309943	1.13209081
H	1.25752004	3.86431673	0.22408348

$(S_0/S_1)_{CI}^{10^\circ}$

C	0.86380072	-0.07043459	0.61587179
C	-0.17703098	0.74643068	0.19502438
C	-1.08616508	-0.08334076	-0.38588103
C	0.58111780	-1.55237511	0.26607766
C	-0.59960345	-1.57278713	-0.33672446
C	-0.28468975	2.32989895	0.35867893
H	-1.22808770	2.82231663	0.13000428
H	-2.03601234	0.22182567	-0.83852314
H	1.77412504	0.26157900	1.12356788
H	-1.16506118	-2.41632345	-0.73241926
H	1.25682708	-2.37698563	0.49059069
C	0.94274126	3.02489954	0.82214728
H	1.23431926	2.69671704	1.84310437
H	1.80950045	2.78079168	0.17080364
H	0.81921163	4.11743149	0.84018417

$(S_0/S_1)_{CI}^{20^\circ}$

C	0.83326731	-0.08996449	0.69191836
C	-0.18251870	0.74004684	0.23773812
C	-1.06098301	-0.07250487	-0.41900296
C	0.56812915	-1.55783620	0.28281470
C	-0.58206976	-1.55759954	-0.37946421
C	-0.30101972	2.30621642	0.43818795
H	-1.27548124	2.77994406	0.32898329
H	-1.97912526	0.25161990	-0.92071021
H	1.71931554	0.22553913	1.25031623
H	-1.12954218	-2.39180069	-0.81771705

H	1.23401060	-2.39004331	0.50932643
C	0.95233892	3.03484574	0.76820441
H	1.32843318	2.75252650	1.77411390
H	1.76549665	2.77265565	0.05868688
H	0.81474128	4.12599887	0.75911133

$(S_0/S_1)_{CI}^{30^\circ}$

C	0.80040469	-0.11138409	0.76434090
C	-0.18697760	0.73561942	0.27854316
C	-1.03478900	-0.06182564	-0.45260337
C	0.55549000	-1.56039960	0.30214930
C	-0.56419824	-1.53951685	-0.41968264
C	-0.30626750	2.27916606	0.51667299
H	-1.29407446	2.73879370	0.53161864
H	-1.91882360	0.27933675	-1.00133662
H	1.64728657	0.18579777	1.38976589
H	-1.08366700	-2.36268663	-0.91009867
H	1.20286101	-2.40336055	0.54227349
C	0.95752168	3.04289641	0.71223507
H	1.43908777	2.78641563	1.67867502
H	1.69973952	2.79046747	-0.07337990
H	0.79139893	4.13032417	0.70333391

$(S_0/S_1)_{CI}^{40^\circ}$

C	0.77388516	-0.13886126	0.83711765
C	-0.18223961	0.73044370	0.32048097
C	-1.00105396	-0.04422639	-0.48395927
C	0.54041014	-1.56269806	0.32108928
C	-0.54704754	-1.51296195	-0.45942320
C	-0.30102360	2.24245140	0.59086094
H	-1.28883384	2.68304798	0.72876109
H	-1.84963732	0.32389443	-1.06973736
H	1.58363909	0.13254643	1.52092243
H	-1.04554475	-2.32309928	-0.99163392
H	1.16653316	-2.42020592	0.56747826
C	0.95273458	3.05128476	0.65380649
H	1.52033635	2.83672723	1.58150447
H	1.63063957	2.79830383	-0.18637701
H	0.75219534	4.13299712	0.63161634

$(S_0/S_1)_{CI}^{50^\circ}$

C	0.74313532	-0.16721295	0.91634843
C	-0.17571495	0.72657029	0.36156211
C	-0.96217891	-0.02624842	-0.51488254
C	0.52075936	-1.56072731	0.35615016
C	-0.52352646	-1.47821031	-0.49593395
C	-0.28971954	2.20821972	0.65540608
H	-1.26351506	2.63145232	0.90910305
H	-1.77051363	0.36717661	-1.13853631
H	1.50593796	0.08101118	1.65960665
H	-0.98648443	-2.27596538	-1.07699063
H	1.11709799	-2.43625985	0.61316323
C	0.94161744	3.05498449	0.58950288
H	1.62330795	2.82801728	1.43312279
H	1.51555223	2.84729578	-0.33457469
H	0.70923750	4.12954056	0.62945991

$(S_0/S_1)_{CI}^{60^\circ}$

C	0.71884546	-0.20144259	0.98568873
C	-0.16585832	0.72158892	0.40162507
C	-0.92399273	-0.00387304	-0.54277843
C	0.50424119	-1.55445644	0.38241343
C	-0.50098840	-1.43789870	-0.53513732
C	-0.27115966	2.17155828	0.72438337
H	-1.21364163	2.57645805	1.09921929

H	-1.69845207	0.42087300	-1.18791023
H	1.43911304	0.02100134	1.77730466
H	-0.92454346	-2.22203852	-1.16227723
H	1.06214958	-2.45314291	0.64732876
C	0.92223240	3.05697251	0.52931200
H	1.68002938	2.87189591	1.31572168
H	1.41668534	2.83699867	-0.43494411
H	0.66033268	4.12514952	0.56255749

$(S_0/S_1)_{CI}^{70^\circ}$

C	0.69833514	-0.25416573	1.05362366
C	-0.14441478	0.70851691	0.44545399
C	-0.88114538	0.02274679	-0.56023071
C	0.47948988	-1.56296845	0.41191096
C	-0.47823659	-1.39015884	-0.57514236
C	-0.23651461	2.13687995	0.78964256
H	-1.13815316	2.51670274	1.28124903
H	-1.62063447	0.47950856	-1.22199548
H	1.38540999	-0.06187527	1.88060104
H	-0.86893056	-2.14913417	-1.25389639
H	0.98950071	-2.48890529	0.68032710
C	0.89751186	3.06622113	0.46900302
H	1.71456723	2.97337596	1.21227213
H	1.33477473	2.81517995	-0.51227740
H	0.57343283	4.11771975	0.46196601

$(S_0/S_1)_{CI}^{80^\circ}$

C	0.68483081	-0.28569208	1.08651814
C	-0.13220284	0.70079973	0.46787649
C	-0.85455897	0.03406316	-0.56744399
C	0.46466071	-1.56999205	0.42778647
C	-0.46181956	-1.36731238	-0.59525329
C	-0.22099061	2.12738189	0.81935641
H	-1.09962320	2.49747616	1.36022319
H	-1.58074069	0.51060992	-1.22832695
H	1.36108475	-0.10562462	1.92399237
H	-0.83270230	-2.11946696	-1.29277714
H	0.94371301	-2.51351607	0.69222448
C	0.88404847	3.07359560	0.44074850
H	1.72940100	3.01971866	1.15577369
H	1.28945627	2.81206193	-0.55046687
H	0.53043598	4.11554111	0.42227566

$(S_0/S_1)_{CI}^{90^\circ}$

C	0.63568229	-0.34813531	1.17750748
C	-0.10054078	0.68675805	0.51390318
C	-0.75691316	0.08180220	-0.60481817
C	0.43205214	-1.58664907	0.46785575
C	-0.42781778	-1.32325000	-0.62833812
C	-0.17147966	2.10085361	0.90348044
H	-0.95459095	2.43392984	1.59313187
H	-1.40619172	0.60307451	-1.31032863
H	1.24294169	-0.21172866	2.07404274
H	-0.77838752	-2.04812788	-1.36443522
H	0.87210231	-2.55045697	0.72748383
C	0.82441674	3.08557233	0.35930417
H	1.76784589	3.05587128	0.93993871
H	1.08438588	2.83350181	-0.68225584
H	0.44148747	4.11662826	0.39603498

$(S_0/S_1)_{CI}^{100^\circ}$

C	0.58760268	-0.38274439	1.23330596
C	-0.07535138	0.68388318	0.54432064
C	-0.67007670	0.11551314	-0.61715284
C	0.39748239	-1.61199190	0.48879426

C	-0.38277310	-1.31234890	-0.64041742
C	-0.13711688	2.09354403	0.94694862
H	-0.85214240	2.40729345	1.71510624
H	-1.26514151	0.66095891	-1.35204137
H	1.14730578	-0.27696389	2.16492623
H	-0.72264192	-2.01508567	-1.40255131
H	0.80820446	-2.58637395	0.75577775
C	0.77632934	3.09917110	0.30770803
H	1.79351496	3.02754983	0.74078251
H	0.88047581	2.89705593	-0.77226584
H	0.41932131	4.13018314	0.44926570

$(S_0/S_1)_{CI}^{110^\circ}$

C	0.52550783	-0.40431400	1.28936171
C	-0.05276582	0.68219303	0.57175437
C	-0.57022429	0.14050208	-0.62622187
C	0.35909369	-1.64113230	0.51395841
C	-0.31133288	-1.31462459	-0.65229228
C	-0.10696096	2.09512391	0.98587025
H	-0.75241771	2.39174384	1.81831958
H	-1.09989111	0.70208051	-1.39886879
H	1.02114254	-0.32487975	2.25974756
H	-0.60345412	-1.99089287	-1.45660462
H	0.71705697	-2.62654372	0.81381989
C	0.72362018	3.11310077	0.26177295
H	1.80282869	2.91140245	0.40592660
H	0.54193241	3.05952533	-0.82728331
H	0.51085742	4.13635933	0.60324671

$(S_0/S_1)_{CI}^{120^\circ}$

C	0.50176822	-0.42120068	1.31466106
C	-0.03604527	0.68050226	0.59652736
C	-0.53291252	0.16374784	-0.61462761
C	0.33017069	-1.66221392	0.51721645
C	-0.28086565	-1.30790624	-0.66382194
C	-0.08114073	2.09423372	1.00789048
H	-0.68238999	2.39133341	1.87040698
H	-1.05199682	0.73534856	-1.38811067
H	0.99070940	-0.36678048	2.29126364
H	-0.55601510	-1.95570535	-1.49703332
H	0.66235400	-2.65320796	0.82772119
C	0.69989170	3.11392253	0.23767236
H	1.79051466	2.94104861	0.33485821
H	0.47244345	3.03764309	-0.84100685
H	0.47850681	4.13887863	0.56888980

$(S_0/S_1)_{CI}^{130^\circ}$

C	0.40810199	-0.44162877	1.36621720
C	0.00355418	0.66714992	0.61825337
C	-0.37672605	0.17153601	-0.63116486
C	0.27374794	-1.70609685	0.53780343
C	-0.18105603	-1.33290762	-0.67723092
C	-0.02082138	2.11463124	1.06366128
H	-0.49625586	2.37746864	2.01088983
H	-0.77111981	0.76494448	-1.46041909
H	0.78459377	-0.41262795	2.39285821
H	-0.37710594	-1.95863081	-1.54801293
H	0.51622130	-2.70861031	0.89097287
C	0.61081347	3.14861999	0.18800587
H	1.60542217	2.81159043	-0.16272372
H	0.00421021	3.31695323	-0.72429291
H	0.72141290	4.11725240	0.69768949

$(S_0/S_1)_{CI}^{140^\circ}$

C	0.36081895	-0.46038623	1.38876896

C	0.03787029	0.65868395	0.63923717
C	-0.29336070	0.18502921	-0.62731890
C	0.22536570	-1.73416108	0.53922930
C	-0.14847065	-1.33956394	-0.68538511
C	0.04166615	2.12918162	1.10354976
H	-0.32612916	2.37509251	2.10080971
H	-0.60901433	0.79725426	-1.47630222
H	0.68723748	-0.45633133	2.43289813
H	-0.31209266	-1.94415126	-1.57722250
H	0.41876760	-2.74307264	0.90405803
C	0.55424137	3.16361008	0.15743367
H	1.50286743	2.83312594	-0.31033077
H	-0.15563097	3.33265366	-0.67791170
H	0.72085637	4.13267928	0.65099360

$(S_0/S_1)_{CI}^{150^\circ}$

C	0.31950300	-0.47370695	1.40542056
C	0.07826711	0.65120954	0.65382468
C	-0.20342018	0.19561999	-0.62777019
C	0.18189606	-1.75320496	0.53800651
C	-0.11345748	-1.34121042	-0.69422170
C	0.11237973	2.14035809	1.13646426
H	-0.14235630	2.37037918	2.17055281
H	-0.45287901	0.82214122	-1.48908537
H	0.58953905	-0.48893106	2.46648540
H	-0.27128839	-1.93060650	-1.59699672
H	0.31282444	-2.76834530	0.91266321
C	0.49790598	3.17415261	0.13615003
H	1.40482020	2.86406869	-0.42290994
H	-0.29601308	3.31445112	-0.62677540
H	0.68727174	4.15326875	0.60069899

$(S_0/S_1)_{CI}^{160^\circ}$

C	0.27993840	-0.48694485	1.42239819
C	0.12067970	0.64388754	0.67016153
C	-0.11955664	0.20783761	-0.62528235
C	0.12982172	-1.76568398	0.53737584
C	-0.09031484	-1.33697774	-0.69953076
C	0.19251480	2.14944528	1.16072189
H	0.05918503	2.36734142	2.22006167
H	-0.30417961	0.84611621	-1.49351794
H	0.49490547	-0.52344574	2.49531082
H	-0.22929032	-1.91009725	-1.61568804
H	0.20912973	-2.78470336	0.91613244
C	0.44209797	3.17845805	0.11735771
H	1.31800729	2.90736857	-0.50856586
H	-0.41423801	3.26291704	-0.58492072
H	0.61629217	4.17412519	0.55049272

$(S_0/S_1)_{CI}^{170^\circ}$

C	0.24455563	-0.48878998	1.42341287
C	0.16579293	0.63996896	0.66861538
C	-0.03000407	0.20520724	-0.63545132
C	0.08730729	-1.77405262	0.54142742
C	-0.08046771	-1.34345544	-0.69938457
C	0.27448465	2.14761925	1.16896311
H	0.26399711	2.34913951	2.23995013
H	-0.13553591	0.84425531	-1.51537362
H	0.39571962	-0.52406827	2.50682277
H	-0.21494476	-1.91524605	-1.61665326
H	0.10863162	-2.79284566	0.92674085
C	0.38455925	3.19050738	0.11465556
H	1.22532080	2.97063111	-0.57796966
H	-0.52442374	3.22753417	-0.52256935
H	0.54000013	4.19323908	0.53932083

$(S_0/S_1)^{180^\circ}_{CI}$

C	0.20504993	-0.48507622	1.42990381
C	0.20922448	0.63744720	0.66435359
C	0.05552297	0.20221546	-0.64540096
C	0.03348269	-1.76985850	0.54425429
C	-0.05631584	-1.34165570	-0.70538370
C	0.36015707	2.14576880	1.17087018
H	0.47404004	2.33592805	2.23675555
H	0.01218286	0.84005807	-1.53270305
H	0.30255971	-0.51815319	2.52052089
H	-0.18245628	-1.91377939	-1.62388276
H	0.00002433	-2.78558963	0.93765503
C	0.33187049	3.19106345	0.11811207
H	1.12977594	3.02651152	-0.63730446
H	-0.62467591	3.16286233	-0.44822846
H	0.45455035	4.20190174	0.53298513

6.2.4 RMS-CASPT2

$(S_0)_{min}$

C	1.05265206	-0.12867298	0.46939447
C	-0.10089913	0.75269415	0.21070102
C	-1.18711393	-0.10518613	-0.30263912
C	0.67913920	-1.41263449	0.13478519
C	-0.71932231	-1.39937796	-0.34747886
C	-0.19181530	2.10246920	0.39481034
H	-1.14383529	2.58579987	0.14223299
H	-2.17612314	0.25607027	-0.58743632
H	2.02100499	0.18499544	0.85489829
H	-1.27330347	-2.27926517	-0.67998326
H	1.30450383	-2.30468183	0.20879864
C	0.90309584	2.98867920	0.91558204
H	0.57540985	3.50356318	1.83408616
H	1.81148490	2.41708125	1.13917735
H	1.15011466	3.76811001	0.17557825

$(S_1)_{min}$

C	0.99224699	-0.11085610	0.45120054
C	-0.11732475	0.73366826	0.20189171
C	-1.14436264	-0.10011559	-0.28702065
C	0.63968007	-1.49822870	0.10741079
C	-0.66737693	-1.49429868	-0.34461050
C	-0.22111169	2.19289812	0.39916081
H	-1.17126816	2.67232827	0.14651933
H	-2.14321273	0.23442726	-0.57947059
H	1.96469340	0.19863853	0.83740595
H	-1.25449556	-2.34796365	-0.68434340
H	1.30522597	-2.35678857	0.20070080
C	0.90501301	3.02869224	0.92269709
H	0.62471939	3.54750712	1.85799500
H	1.79302778	2.41799124	1.13319608
H	1.19953862	3.81174426	0.19977424

$(S_0/S_1)_{MECI}$

C	0.70958282	-0.22042535	1.02823592
C	-0.16736040	0.71087003	0.42225224
C	-0.88249659	-0.00181189	-0.57810267
C	0.52851950	-1.54212398	0.39380652
C	-0.43891278	-1.40562156	-0.58551818
C	-0.29095221	2.14128639	0.75584612
H	-1.18044180	2.49920800	1.28429789
H	-1.63490504	0.42936585	-1.24257774
H	1.39534126	-0.00304454	1.85009360
H	-0.81127159	-2.18034799	-1.25658509

H	1.06899980	-2.44956187	0.66558562
C	0.86459824	3.06469841	0.50580561
H	1.70732316	2.83663140	1.18808110
H	1.24994618	2.93169656	-0.52067848
H	0.58702225	4.11882457	0.65196471

$(S_0/S_1)_{CI}^{0^\circ}$

C	0.94303980	-0.11004088	0.43497438
C	-0.13692182	0.70988565	0.19264486
C	-1.12000838	-0.10953247	-0.27755720
C	0.61561358	-1.59438306	0.08525075
C	-0.63337061	-1.60055045	-0.34793610
C	-0.25067254	2.30861701	0.40821366
H	-1.20171021	2.77752416	0.15445915
H	-2.12604156	0.20362233	-0.57810152
H	1.91807435	0.20054537	0.82223885
H	-1.24662218	-2.42992794	-0.69976093
H	1.32002153	-2.41853495	0.19269903
C	0.91271878	3.08791260	0.93429989
H	0.67959274	3.60650118	1.88428522
H	1.77367174	2.43350014	1.13233749
H	1.25760756	3.86450530	0.22445964

$(S_0/S_1)_{CI}^{10^\circ}$

C	0.86170771	-0.06872543	0.61551523
C	-0.17804213	0.74616207	0.19438524
C	-1.08761118	-0.08540188	-0.38683268
C	0.58053993	-1.55234346	0.26683281
C	-0.60037701	-1.57356139	-0.33540363
C	-0.28369861	2.33041406	0.35848395
H	-1.22643662	2.82413311	0.12992278
H	-2.03697448	0.21864319	-0.84084537
H	1.77182273	0.26436220	1.12311585
H	-1.16422971	-2.41784586	-0.73200682
H	1.25683808	-2.37605265	0.49264508
C	0.94430601	3.02471769	0.82215870
H	1.23734335	2.69391085	1.84175306
H	1.81051251	2.78421209	0.16884024
H	0.81929220	4.11701941	0.84394273

$(S_0/S_1)_{CI}^{20^\circ}$

C	0.82712310	-0.08271402	0.69052155
C	-0.18964424	0.74153364	0.23435631
C	-1.06547259	-0.07707542	-0.42222504
C	0.56869401	-1.55336933	0.28479233
C	-0.58026149	-1.55860196	-0.37964621
C	-0.30162964	2.30861316	0.43874194
H	-1.27307445	2.78921058	0.33165164
H	-1.98538319	0.23949572	-0.92521195
H	1.70889954	0.23893231	1.25259681
H	-1.12303454	-2.39570128	-0.81892531
H	1.23756624	-2.38167443	0.51557541
C	0.95616160	3.02922184	0.76992692
H	1.34051777	2.73895528	1.77090342
H	1.76321442	2.77208415	0.05237010
H	0.82131623	4.12073375	0.76707925

$(S_0/S_1)_{CI}^{30^\circ}$

C	0.80014390	-0.10955058	0.76470450
C	-0.18755798	0.73567932	0.27826363
C	-1.03554542	-0.06204855	-0.45298025
C	0.55528870	-1.55935093	0.30251677
C	-0.56481074	-1.53969937	-0.41855185
C	-0.30650405	2.27909675	0.51646205
H	-1.29391111	2.73948720	0.53158095

H	-1.91959822	0.27840970	-1.00209563
H	1.64664587	0.18843472	1.39028963
H	-1.08393117	-2.36307350	-0.90906381
H	1.20502917	-2.40092565	0.54110450
C	0.95780877	3.04156613	0.71187752
H	1.43753455	2.78640715	1.67969148
H	1.70095543	2.78601114	-0.07152961
H	0.79344508	4.12920048	0.70023731

$(S_0/S_1)_{CI}^{40^\circ}$

C	0.77196151	-0.13450496	0.83763004
C	-0.18440484	0.73148296	0.31870235
C	-1.00156710	-0.04509277	-0.48603974
C	0.54197799	-1.55979674	0.32361121
C	-0.54669152	-1.51204362	-0.45511925
C	-0.30313259	2.24419821	0.58910798
H	-1.29008265	2.68716276	0.72650805
H	-1.84750436	0.32052560	-1.07675357
H	1.57560758	0.13870476	1.52736675
H	-1.04185974	-2.32362379	-0.98806619
H	1.16856630	-2.41637105	0.57136038
C	0.95374979	3.04770070	0.65203474
H	1.53103748	2.81426432	1.56927164
H	1.62189536	2.80717468	-0.19957342
H	0.75543957	4.12986294	0.65246623

$(S_0/S_1)_{CI}^{50^\circ}$

C	0.74311933	-0.16604103	0.91455283
C	-0.17527976	0.72675669	0.35958620
C	-0.96146704	-0.02538188	-0.51768341
C	0.52227128	-1.55907964	0.35468931
C	-0.52514594	-1.47844019	-0.49224390
C	-0.28879355	2.20676871	0.65427449
H	-1.26229330	2.63062870	0.90796725
H	-1.77008426	0.36747028	-1.14125114
H	1.50423527	0.08242830	1.65931183
H	-0.98881885	-2.27747123	-1.07083369
H	1.11705583	-2.43528524	0.61360055
C	0.94212652	3.05422225	0.58985936
H	1.62278630	2.82647180	1.43360868
H	1.51698530	2.84803459	-0.33426560
H	0.70829566	4.12856187	0.63133445

$(S_0/S_1)_{CI}^{60^\circ}$

C	0.71974795	-0.20670139	0.99116930
C	-0.15962461	0.72014776	0.40453361
C	-0.91832598	-0.00163805	-0.54124095
C	0.50074213	-1.56000466	0.38731999
C	-0.50256003	-1.43825113	-0.53222170
C	-0.26774973	2.17079970	0.71865009
H	-1.21523613	2.57029207	1.08706768
H	-1.68768238	0.42443885	-1.19085324
H	1.43849006	0.01172642	1.78493627
H	-0.92954474	-2.21874138	-1.16210299
H	1.05755313	-2.45882509	0.65477857
C	0.92062396	3.06241580	0.52624131
H	1.68900751	2.86078462	1.29768136
H	1.40391818	2.86464792	-0.44927892
H	0.65563346	4.12855254	0.58582684

$(S_0/S_1)_{CI}^{70^\circ}$

C	0.67987109	-0.23883141	1.07446793
C	-0.15121318	0.71417541	0.43967136
C	-0.86463261	0.01550356	-0.57569896
C	0.47802478	-1.55616881	0.43990727

C	-0.46131007	-1.39667181	-0.56612098
C	-0.25086084	2.14517458	0.77196737
H	-1.16101250	2.53066629	1.24192014
H	-1.58957802	0.46452123	-1.25857990
H	1.34671487	-0.03731150	1.91561387
H	-0.83444549	-2.16364531	-1.24565390
H	0.98513814	-2.47774618	0.72815799
C	0.89647849	3.06138832	0.46309124
H	1.79063115	2.78508618	1.05454849
H	1.18784133	2.97171802	-0.59965595
H	0.65334566	4.11178541	0.67887126

$(S_0/S_1)_{CI}^{80^\circ}$

C	0.66491607	-0.27051306	1.10918618
C	-0.13958608	0.70705487	0.46420546
C	-0.83781399	0.02787827	-0.58103700
C	0.46207047	-1.56245158	0.45758721
C	-0.44284190	-1.37277002	-0.58636442
C	-0.23761244	2.13692407	0.80216950
H	-1.12691858	2.51245367	1.32067647
H	-1.54948947	0.49660729	-1.26294641
H	1.32239438	-0.08145364	1.95946336
H	-0.79414179	-2.13138608	-1.28696738
H	0.93996882	-2.50136502	0.73995561
C	0.88256589	3.06759719	0.43294121
H	1.80496098	2.82397050	0.99562407
H	1.13314767	2.96040166	-0.63740173
H	0.62337277	4.11669587	0.63541508

$(S_0/S_1)_{CI}^{90^\circ}$

C	0.61362244	-0.33419982	1.20069017
C	-0.10840747	0.69269868	0.51065726
C	-0.73830462	0.07483890	-0.61700048
C	0.42827617	-1.58007785	0.49935470
C	-0.40549190	-1.32835306	-0.62014023
C	-0.18847705	2.11063478	0.88699967
H	-0.98596705	2.45084836	1.55585341
H	-1.37123346	0.58840663	-1.34283400
H	1.19887963	-0.18798040	2.11025179
H	-0.73311740	-2.06010082	-1.35991195
H	0.86082525	-2.54127707	0.78030419
C	0.82571080	3.07963206	0.34958441
H	1.83097893	2.85999370	0.75789314
H	0.90737648	2.98499071	-0.74830621
H	0.57032206	4.11958918	0.59911137

$(S_0/S_1)_{CI}^{100^\circ}$

C	0.57222917	-0.37399798	1.24801623
C	-0.08003767	0.68810357	0.54253153
C	-0.65932538	0.11137360	-0.62411469
C	0.39192100	-1.60771926	0.51029948
C	-0.37242649	-1.31563700	-0.63296997
C	-0.14511062	2.10001263	0.93618721
H	-0.86950429	2.42050112	1.69237300
H	-1.24312978	0.65203898	-1.37142515
H	1.11742293	-0.26193376	2.18733393
H	-0.70089411	-2.02394982	-1.39479620
H	0.79762332	-2.58078722	0.79006537
C	0.78191109	3.09453242	0.29927910
H	1.82402394	2.91454417	0.62662947
H	0.77709400	2.98240239	-0.80003898
H	0.51319571	4.13016014	0.55313692

$(S_0/S_1)_{CI}^{110^\circ}$

C	0.51959149	-0.40165808	1.29461750

C	-0.05315952	0.68434922	0.57223431
C	-0.57128100	0.14010290	-0.62458594
C	0.34930334	-1.64096969	0.52385755
C	-0.31827694	-1.31613544	-0.64455708
C	-0.10248295	2.09886836	0.98016493
H	-0.74788878	2.40184128	1.81036700
H	-1.09808007	0.70073812	-1.39991179
H	1.01478624	-0.32075125	2.26514003
H	-0.61238590	-1.99393813	-1.44685871
H	0.70384488	-2.62629896	0.82811050
C	0.73328108	3.11019763	0.25262936
H	1.81142583	2.90005200	0.39233542
H	0.54660230	3.05815613	-0.83574304
H	0.52971281	4.13508990	0.59470721

$(S_0/S_1)_{CI}^{120°}$

C	0.49587244	-0.41850341	1.31971810
C	-0.03654689	0.68247145	0.59735030
C	-0.53381263	0.16356179	-0.61299418
C	0.32088330	-1.66118615	0.52682724
C	-0.28748452	-1.30922723	-0.65627396
C	-0.07729962	2.09860575	1.00316505
H	-0.67854777	2.40204266	1.86344792
H	-1.05063100	0.73434576	-1.38860005
H	0.98425410	-0.36234719	2.29650627
H	-0.56408545	-1.95929908	-1.48723835
H	0.64871288	-2.65243186	0.84113455
C	0.70935724	3.11067859	0.22846427
H	1.79877701	2.92853734	0.32207620
H	0.47752846	3.03421815	-0.84936565
H	0.49801526	4.13817742	0.55828955

$(S_0/S_1)_{CI}^{130°}$

C	0.41841706	-0.44834290	1.35805294
C	0.00894944	0.66713542	0.62049261
C	-0.40207003	0.17620759	-0.61789961
C	0.25425396	-1.71032064	0.53383791
C	-0.22331372	-1.33105018	-0.66981854
C	0.00842896	2.11832070	1.06784917
H	-0.44417542	2.38334897	2.02515612
H	-0.81131425	0.77331254	-1.43747000
H	0.82060342	-0.42286844	2.37536825
H	-0.44710277	-1.95509635	-1.53523509
H	0.49245836	-2.71630070	0.87918580
C	0.63168173	3.14838479	0.17867618
H	1.70989735	2.94574959	0.01203328
H	0.16062687	3.13978407	-0.82173030
H	0.52765185	4.16137952	0.59400852

$(S_0/S_1)_{CI}^{140°}$

C	0.35732327	-0.45741907	1.39190546
C	0.03871545	0.66018169	0.63878248
C	-0.29150037	0.18402014	-0.62628417
C	0.21965847	-1.73265691	0.54533557
C	-0.14974611	-1.33971109	-0.68059690
C	0.04597635	2.13167704	1.09968679
H	-0.32239555	2.38179729	2.09522841
H	-0.60985321	0.79471435	-1.47600281
H	0.68154737	-0.45075591	2.43731659
H	-0.32470510	-1.94650402	-1.56881520
H	0.39961321	-2.74234674	0.91453350
C	0.56312697	3.16126184	0.15144360
H	1.51276367	2.82695879	-0.31244446
H	-0.14492348	3.32493139	-0.68649566
H	0.72939186	4.13349520	0.63891405

$(S_0/S_1)_{CI}^{150^\circ}$

C	0.31354032	-0.46999915	1.40849352
C	0.07780767	0.65240894	0.65013591
C	-0.20097232	0.19153408	-0.62912581
C	0.17456485	-1.75334446	0.54544802
C	-0.12192116	-1.34636963	-0.68829137
C	0.11587419	2.14201758	1.12990410
H	-0.14127515	2.37289494	2.16253561
H	-0.45193839	0.81581385	-1.49242571
H	0.58844785	-0.47844855	2.46887205
H	-0.28635819	-1.93940516	-1.58761487
H	0.29912398	-2.76696228	0.92609923
C	0.50771212	3.17521834	0.13141934
H	1.41640307	2.86363765	-0.42449363
H	-0.28223753	3.31637126	-0.63503309
H	0.69622149	4.15427656	0.59658393

$(S_0/S_1)_{CI}^{160^\circ}$

C	0.27477182	-0.48162653	1.42172179
C	0.11937822	0.64628117	0.66498828
C	-0.12014995	0.20272671	-0.62702686
C	0.12309477	-1.76493924	0.54589410
C	-0.09916539	-1.34272862	-0.69336136
C	0.19593549	2.15048311	1.15633427
H	0.06266701	2.37061261	2.21438275
H	-0.29422327	0.83794182	-1.50057115
H	0.48867369	-0.50866621	2.49555092
H	-0.24482347	-1.92180964	-1.60469245
H	0.19873008	-2.78257521	0.92892247
C	0.45030283	3.17952092	0.11369078
H	1.32571969	2.90292368	-0.51136244
H	-0.40541516	3.26707096	-0.58891539
H	0.62949645	4.17442843	0.54695153

$(S_0/S_1)_{CI}^{170^\circ}$

C	0.23580728	-0.48453344	1.42910583
C	0.16240007	0.64105373	0.66550550
C	-0.03299539	0.20207493	-0.63539685
C	0.07409818	-1.77069543	0.54904234
C	-0.07550395	-1.34611592	-0.69782909
C	0.27769628	2.15199445	1.16646670
H	0.26664347	2.35625703	2.23555156
H	-0.13988087	0.83941672	-1.51819604
H	0.39112768	-0.51524685	2.51305373
H	-0.21038691	-1.92113064	-1.61345306
H	0.09265580	-2.78777414	0.94018683
C	0.39403469	3.18819106	0.10912139
H	1.22556097	2.95303359	-0.58938185
H	-0.52148930	3.23264226	-0.51940850
H	0.56522478	4.19047661	0.52813875

$(S_0/S_1)_{CI}^{180^\circ}$

C	0.20073092	-0.48853976	1.43701303
C	0.19583023	0.63858374	0.67310410
C	0.03712268	0.20543339	-0.63398067
C	0.03007441	-1.77020162	0.54918669
C	-0.03588204	-1.34013799	-0.70233243
C	0.34211511	2.15332934	1.17015075
H	0.42407688	2.35676189	2.23616790
H	-0.05751174	0.84659789	-1.51502456
H	0.31679137	-0.52620402	2.52551355
H	-0.16743576	-1.91155058	-1.62051117
H	0.00395258	-2.78733346	0.93970046
C	0.35426656	3.18572198	0.10499486
H	1.14694332	2.97837989	-0.64359599

H	-0.60148987	3.19264848	-0.46549573
H	0.51540815	4.19615478	0.50761645

6.2.5 XDW-CASPT2

$(S_0)_{min}$

C	1.07229501	-0.14858720	0.47298185
C	-0.08146963	0.74958535	0.21690082
C	-1.16745511	-0.08904045	-0.29327719
C	0.67698797	-1.42277639	0.13242908
C	-0.71967786	-1.39600909	-0.34707001
C	-0.19432413	2.09884557	0.39336021
H	-1.15302074	2.56540270	0.13582747
H	-2.15304356	0.28601066	-0.57471606
H	2.04440621	0.15302871	0.85787904
H	-1.28776491	-2.26514774	-0.68270736
H	1.28980724	-2.32449418	0.20062269
C	0.88965620	2.99831187	0.91247203
H	0.55583807	3.51296063	1.82898131
H	1.80218127	2.43393251	1.13859620
H	1.13057675	3.77762104	0.17022710

$(S_1)_{min}$

C	1.00425616	-0.12457105	0.45314619
C	-0.10913068	0.73225726	0.20447423
C	-1.13194720	-0.08802780	-0.28078970
C	0.63841791	-1.50577259	0.10577703
C	-0.66618393	-1.49560804	-0.34438173
C	-0.22477930	2.19358529	0.39796053
H	-1.17884165	2.66337424	0.14239893
H	-2.12939214	0.25360808	-0.57161225
H	1.97907045	0.17737640	0.83894498
H	-1.26260285	-2.34178295	-0.68611828
H	1.29788723	-2.36978875	0.19609448
C	0.89691687	3.03530000	0.92096351
H	0.61489595	3.55416286	1.85581838
H	1.78664092	2.42700733	1.13240819
H	1.18978505	3.81852374	0.19742272

$(S_0/S_1)_{MECI}$

C	0.71615926	-0.22368766	1.03485830
C	-0.15195768	0.71082888	0.41203203
C	-0.86138053	-0.00300069	-0.59035352
C	0.51572952	-1.54247931	0.40253600
C	-0.44434077	-1.40998293	-0.58869948
C	-0.28448423	2.13702173	0.75865898
H	-1.16731430	2.47840057	1.30898804
H	-1.60445231	0.43107885	-1.26352561
H	1.39242168	-0.00896127	1.86482406
H	-0.82085383	-2.18528072	-1.25641983
H	1.04226260	-2.45560123	0.68488063
C	0.85802108	3.07456948	0.50036355
H	1.72057994	2.83289566	1.15257106
H	1.21600582	2.97108169	-0.53950362
H	0.57859653	4.12276099	0.68129660

$(S_0/S_1)_{CI}^{0^\circ}$

C	0.95066126	-0.11974449	0.43378942
C	-0.12562534	0.71079059	0.19553383
C	-1.11175386	-0.10259015	-0.27541703
C	0.61624904	-1.60341633	0.08024597
C	-0.63299520	-1.60279824	-0.35012060
C	-0.25326696	2.31012490	0.40813502
H	-1.20875104	2.76809001	0.15179059
H	-2.11706890	0.21760748	-0.57175658

H	1.92918257	0.18116833	0.81920921
H	-1.25680039	-2.42647590	-0.69685550
H	1.31555895	-2.43199266	0.18944127
C	0.90626349	3.09619834	0.93456305
H	0.67024179	3.61933833	1.88135860
H	1.76766765	2.44416787	1.13990857
H	1.25542971	3.86917591	0.22268136

$(S_0/S_1)_{CI}^{10^\circ}$

C	0.93517494	-0.12843227	0.48055056
C	-0.13216742	0.70683805	0.21870763
C	-1.10450869	-0.09872377	-0.29226008
C	0.60887025	-1.60666661	0.09900721
C	-0.61863316	-1.59485194	-0.38906825
C	-0.26531619	2.30316115	0.44554137
H	-1.23812461	2.75169715	0.24235673
H	-2.10756591	0.22304382	-0.59452050
H	1.90898696	0.16955750	0.88001259
H	-1.23299567	-2.41335650	-0.76382148
H	1.30651806	-2.43640465	0.21006682
C	0.91245689	3.09957910	0.91375049
H	0.72156193	3.64027361	1.86029815
H	1.78697318	2.45421343	1.08138817
H	1.22376220	3.85971591	0.17049777

$(S_0/S_1)_{CI}^{20^\circ}$

C	0.83866955	-0.09047742	0.68660370
C	-0.18072793	0.74069409	0.24114667
C	-1.05903630	-0.07379375	-0.41680330
C	0.58152945	-1.56019832	0.27344819
C	-0.57119035	-1.56275445	-0.38465733
C	-0.31140181	2.30842999	0.44575522
H	-1.28892552	2.77619544	0.34079275
H	-1.98097225	0.24677673	-0.91402509
H	1.72168970	0.22354851	1.25044405
H	-1.11700602	-2.39454020	-0.83017049
H	1.25069845	-2.39002780	0.49952926
C	0.94151152	3.03873939	0.77345687
H	1.32769475	2.75719059	1.77630845
H	1.75009862	2.78002078	0.05695767
H	0.80236089	4.12984041	0.76372055

$(S_0/S_1)_{CI}^{30^\circ}$

C	0.81148532	-0.11870272	0.76019070
C	-0.17763151	0.73350560	0.28672715
C	-1.02983981	-0.05686474	-0.44746100
C	0.56936320	-1.56608053	0.28992208
C	-0.55226189	-1.54032461	-0.42762540
C	-0.31646656	2.27704235	0.52497944
H	-1.30986566	2.72401192	0.54182466
H	-1.91934205	0.28878970	-0.98477198
H	1.66129577	0.17074923	1.38497791
H	-1.07864019	-2.35869165	-0.91912700
H	1.22137874	-2.40874549	0.51900829
C	0.94207347	3.05009893	0.71695961
H	1.41524623	2.82087130	1.69460140
H	1.69239882	2.77700963	-0.05392282
H	0.77579887	4.13697508	0.67622413

$(S_0/S_1)_{CI}^{40^\circ}$

C	0.78385704	-0.15103697	0.83217969
C	-0.17097510	0.72648272	0.33187991
C	-0.99756601	-0.03793684	-0.47721396
C	0.55590083	-1.57046617	0.31087893
C	-0.53020883	-1.50931661	-0.46953535

C	-0.31115223	2.24159665	0.60042916
H	-1.30522248	2.66941915	0.73931255
H	-1.85370591	0.33466814	-1.04769679
H	1.59560278	0.11081345	1.51599325
H	-1.03292736	-2.31323398	-1.00781881
H	1.18201233	-2.43081308	0.54611219
C	0.93672059	3.05944625	0.65795789
H	1.49839442	2.87845652	1.59751768
H	1.62307071	2.78248768	-0.16690085
H	0.73119197	4.13907707	0.59941167

$(S_0/S_1)_{CI}^{50^\circ}$

C	0.75069013	-0.18214728	0.90447201
C	-0.16614868	0.72116246	0.37448097
C	-0.96664834	-0.01913074	-0.50659109
C	0.53721617	-1.57191963	0.34328281
C	-0.50872426	-1.47301730	-0.50388084
C	-0.29650156	2.20425203	0.66976515
H	-1.27329052	2.61739839	0.92951803
H	-1.78828125	0.38062314	-1.10845906
H	1.52068956	0.05489084	1.64409169
H	-0.97092641	-2.26165190	-1.09862087
H	1.14365616	-2.44631258	0.57819162
C	0.92656406	3.06267218	0.59560988
H	1.57939124	2.90689476	1.47771963
H	1.53647311	2.80127153	-0.29029310
H	0.68083333	4.13465808	0.55322032

$(S_0/S_1)_{CI}^{60^\circ}$

C	0.71796778	-0.21024437	0.97609475
C	-0.16227254	0.71922751	0.41013881
C	-0.92682289	-0.00304243	-0.53955198
C	0.51832273	-1.56709210	0.37820340
C	-0.48544810	-1.43367034	-0.53856196
C	-0.27795700	2.16991015	0.73366643
H	-1.22318850	2.56896274	1.11102328
H	-1.70729918	0.42140796	-1.17665378
H	1.44203250	0.00700645	1.76611901
H	-0.90709300	-2.21163185	-1.17654157
H	1.09476685	-2.45866009	0.62449861
C	0.90951634	3.06282402	0.53543993
H	1.66418772	2.90011231	1.33015852
H	1.41248404	2.83601172	-0.42254804
H	0.63579598	4.12852230	0.55102174

$(S_0/S_1)_{CI}^{70^\circ}$

C	0.68073395	-0.24781998	1.05139349
C	-0.15266087	0.71379130	0.44532418
C	-0.87682106	0.01361581	-0.57117029
C	0.49279347	-1.56672970	0.42482188
C	-0.46401095	-1.39509045	-0.56465122
C	-0.25372906	2.14111854	0.78991477
H	-1.15824708	2.52311786	1.27347744
H	-1.60679655	0.46601904	-1.24702369
H	1.36476773	-0.04901409	1.88065822
H	-0.83233700	-2.15688049	-1.25329452
H	1.03426389	-2.47826523	0.67914465
C	0.88501138	3.06643170	0.47622337
H	1.74872331	2.87815021	1.14385677
H	1.24561863	2.89883011	-0.55453652
H	0.59768292	4.12236935	0.58836863

$(S_0/S_1)_{CI}^{80^\circ}$

C	0.65021571	-0.31424326	1.12723643
C	-0.12038540	0.69553903	0.50173718

C	-0.81184583	0.04961641	-0.58391371
C	0.46451559	-1.58484931	0.46501270
C	-0.40281676	-1.33928784	-0.61172892
C	-0.22855772	2.11653177	0.84615661
H	-1.09142824	2.47118443	1.42015549
H	-1.49054098	0.55789107	-1.27448896
H	1.31587024	-0.15368541	1.97869535
H	-0.73894664	-2.08344942	-1.33372903
H	0.90923228	-2.53779002	0.75719413
C	0.84443544	3.07904768	0.41018507
H	1.79885319	2.91339682	0.94652227
H	1.06090074	2.93556352	-0.66238352
H	0.54549109	4.12417852	0.57585606

$(S_0/S_1)_{CI}^{90^\circ}$

C	0.61011142	-0.32848528	1.19970892
C	-0.11329636	0.69216097	0.50666006
C	-0.72840340	0.06617996	-0.62762946
C	0.44789413	-1.58255684	0.50327434
C	-0.37460667	-1.32798164	-0.62409207
C	-0.20537862	2.10862236	0.88781485
H	-1.00962184	2.44209213	1.55182017
H	-1.36066357	0.56805752	-1.36157419
H	1.18431983	-0.17117720	2.11555386
H	-0.68002324	-2.06033613	-1.37422417
H	0.87960794	-2.54095578	0.79162458
C	0.80777414	3.08341566	0.36013315
H	1.83055007	2.78617684	0.66007051
H	0.79726865	3.08902359	-0.74569238
H	0.61946021	4.10540782	0.71905899

$(S_0/S_1)_{CI}^{100^\circ}$

C	0.56906629	-0.36922468	1.25353102
C	-0.08362030	0.68646019	0.53995680
C	-0.64420232	0.10460262	-0.63409589
C	0.40957885	-1.60571788	0.51577029
C	-0.34261899	-1.31819524	-0.63745567
C	-0.16466020	2.09813531	0.93412932
H	-0.90024664	2.41270663	1.68185263
H	-1.22536140	0.63929204	-1.38788002
H	1.10168130	-0.25278060	2.19967379
H	-0.64781300	-2.02791891	-1.40797448
H	0.81912193	-2.57521641	0.80229738
C	0.76347895	3.09738128	0.30680630
H	1.81595265	2.82340387	0.50875621
H	0.64918080	3.10096290	-0.79364653
H	0.58545475	4.11575288	0.68078602

$(S_0/S_1)_{CI}^{110^\circ}$

C	0.52620775	-0.40509505	1.28454151
C	-0.05128919	0.68151581	0.57507443
C	-0.57075338	0.14430586	-0.62863194
C	0.36814182	-1.64747614	0.51542178
C	-0.30266794	-1.31186015	-0.64637565
C	-0.11102376	2.09599702	0.98786390
H	-0.75747857	2.39288543	1.81938597
H	-1.10442449	0.70530358	-1.39860075
H	1.02599100	-0.32865818	2.25340126
H	-0.59590995	-1.98723913	-1.45195332
H	0.72448313	-2.63179752	0.81931411
C	0.71786267	3.11360213	0.26035738
H	1.79825917	2.91629516	0.40376222
H	0.53518436	3.05394084	-0.82815712
H	0.50241008	4.13792433	0.59710340

$(S_0/S_1)_{CI}^{120^\circ}$

C	0.46595197	-0.42456387	1.32877162
C	-0.02357902	0.67567596	0.60143946
C	-0.46750591	0.16269549	-0.63283762
C	0.33010634	-1.68049681	0.53300969
C	-0.23119434	-1.32257112	-0.65778257
C	-0.07456602	2.10577426	1.02611887
H	-0.64446541	2.38521618	1.91576093
H	-0.92915949	0.74085855	-1.43657258
H	0.90157149	-0.37391613	2.33031725
H	-0.47499706	-1.97252394	-1.49934989
H	0.62086591	-2.67591581	0.86862970
C	0.66018936	3.13173881	0.21958324
H	1.71667798	2.83413832	0.07490473
H	0.22372753	3.21586659	-0.79464635
H	0.63136938	4.12766750	0.68516072

$(S_0/S_1)^{130°}_{CI}$

C	0.41492573	-0.44988040	1.36138916
C	0.00937642	0.66528612	0.63015521
C	-0.37992172	0.18010527	-0.62394139
C	0.28043160	-1.71467332	0.53869724
C	-0.18435319	-1.33123750	-0.66840569
C	-0.01916934	2.11916622	1.06686156
H	-0.49203985	2.38492998	2.01401470
H	-0.77390624	0.77995389	-1.44855908
H	0.80385999	-0.43050645	2.38365374
H	-0.38602146	-1.95066669	-1.54289034
H	0.51469299	-2.71946067	0.88988936
C	0.60542159	3.14898458	0.18030250
H	1.63142461	2.85315397	-0.11607634
H	0.03329693	3.25627721	-0.76228957
H	0.64697465	4.13821179	0.65970612

$(S_0/S_1)^{140°}_{CI}$

C	0.36425633	-0.47788227	1.38909935
C	0.04708896	0.65835846	0.66400237
C	-0.28438080	0.19538148	-0.60829469
C	0.23024189	-1.74074295	0.53941029
C	-0.13474891	-1.33722590	-0.68211027
C	0.04014908	2.13574767	1.10099782
H	-0.33274029	2.39171715	2.09171963
H	-0.61560051	0.81279540	-1.44575487
H	0.70331016	-0.49428672	2.42966149
H	-0.30842901	-1.93207502	-1.57963009
H	0.40959149	-2.75223946	0.90126878
C	0.54717293	3.16559370	0.14552837
H	1.52365905	2.86633256	-0.28722059
H	-0.14459053	3.28336372	-0.71189530
H	0.66001286	4.15480618	0.61572488

$(S_0/S_1)^{150°}_{CI}$

C	0.32424940	-0.50522968	1.41060932
C	0.08988573	0.64351273	0.69559719
C	-0.19343309	0.21399132	-0.59532169
C	0.18816530	-1.76997892	0.54206534
C	-0.12113479	-1.33366704	-0.67638211
C	0.10889038	2.15159045	1.12937523
H	-0.15201722	2.40780204	2.15564926
H	-0.43147204	0.86678832	-1.44198937
H	0.58843682	-0.55795078	2.47071170
H	-0.27004742	-1.89743124	-1.59682481
H	0.33453133	-2.79174289	0.89142905
C	0.48577847	3.17171964	0.10995884
H	1.43181727	2.90556474	-0.40518094
H	-0.28260727	3.25553292	-0.68672220
H	0.60394982	4.16914239	0.55953237

$(S_0/S_1)_{CI}^{160^\circ}$

C	0.28047632	-0.47815428	1.41726247
C	0.11711930	0.64738010	0.65968792
C	-0.11415292	0.19748327	-0.63351874
C	0.14315527	-1.76801737	0.53897745
C	-0.07776737	-1.34940031	-0.70103643
C	0.18031891	2.15060070	1.16216214
H	0.04197461	2.36179866	2.22133785
H	-0.29107157	0.82750402	-1.50992834
H	0.49112646	-0.50214611	2.49212878
H	-0.21271222	-1.92860065	-1.61425378
H	0.23034135	-2.78323798	0.92619455
C	0.42999855	3.18537134	0.12362306
H	1.31319185	2.92177871	-0.49648110
H	-0.42145257	3.26629087	-0.58492077
H	0.59444673	4.18099303	0.56127210

$(S_0/S_1)_{CI}^{170^\circ}$

C	0.24349026	-0.48299826	1.42586321
C	0.16257833	0.64366443	0.66519650
C	-0.02466617	0.20181729	-0.63850022
C	0.09598774	-1.77392636	0.54006542
C	-0.05676667	-1.34874295	-0.70564409
C	0.26320246	2.15414971	1.17520033
H	0.24675677	2.35321568	2.24505152
H	-0.13962772	0.83761735	-1.52087896
H	0.39440629	-0.51522390	2.51051703
H	-0.18392227	-1.92217300	-1.62354387
H	0.12289641	-2.79056689	0.93245629
C	0.37392275	3.18995115	0.11689955
H	1.22242993	2.97303094	-0.56774313
H	-0.53158042	3.21258618	-0.52724632
H	0.51588502	4.19724262	0.53481392

$(S_0/S_1)_{CI}^{180^\circ}$

C	0.20627034	-0.48403511	1.43154571
C	0.20761943	0.64018418	0.66593359
C	0.06099419	0.20068619	-0.64380674
C	0.04433663	-1.77369946	0.54342061
C	-0.04218690	-1.34744751	-0.70742047
C	0.34913193	2.15211101	1.17484913
H	0.45793968	2.34315544	2.24057196
H	0.02030548	0.83622532	-1.53291229
H	0.30205090	-0.51794738	2.52248853
H	-0.16154228	-1.91800638	-1.62809832
H	0.01670311	-2.78862873	0.94008087
C	0.31865443	3.19263491	0.11681948
H	1.13292728	3.03895116	-0.62441303
H	-0.62711513	3.14840618	-0.46595002
H	0.41890361	4.20705417	0.52939816

6.3 6,6-dimethyl-Fulvene

6.3.1 CASSCF

$(S_0)_{min}$

C	1.14692151	-0.16847965	0.04816053
C	-0.02204199	0.73632451	0.04783710
C	-1.19981395	-0.15693643	0.05725412
C	0.70523785	-1.45302549	0.05677894
C	-0.77073503	-1.44576532	0.06250266
C	-0.01539153	2.09422856	0.04040513
H	-2.22636418	0.16308378	0.05953969
H	2.17655793	0.14142459	0.04251708

H	-1.39434275	-2.32602543	0.06965036
H	1.32019294	-2.33937752	0.05915894
C	1.25276887	2.91186618	0.03110294
H	1.28301085	3.56402291	0.90645091
H	2.15039899	2.30463836	0.02902970
H	1.27446231	3.55731979	-0.84943605
C	-1.27548065	2.92430257	0.04081632
H	-1.29135592	3.57859011	0.91494383
H	-1.29882018	3.56806032	-0.84092919
H	-2.17902169	2.32594800	0.04824291

$(S_1)_{min}$

C	1.09553919	-0.16696407	0.04842617
C	-0.02232825	0.67857938	0.04750842
C	-1.14842059	-0.15592979	0.05657467
C	0.64620071	-1.56877517	0.05841336
C	-0.71282865	-1.56209226	0.06335048
C	-0.01494402	2.18754524	0.03755656
H	-2.17838528	0.15738684	0.05840546
H	2.12852159	0.13620945	0.04284644
H	-1.37305795	-2.41402695	0.07086509
H	1.29805687	-2.42716205	0.06116675
C	1.26729527	2.96541871	0.03035476
H	1.33459515	3.61722682	0.90762236
H	2.14776393	2.33017708	0.02677861
H	1.32567190	3.61479708	-0.84928084
C	-1.28948416	2.97799889	0.04039914
H	-1.34176668	3.63299239	0.91629337
H	-1.35009748	3.62535580	-0.84059738
H	-2.17614820	2.35146246	0.04734254

$(S_0/S_1)_{MECI}$

C	0.85700024	-0.02209712	0.69429895
C	-0.07708932	0.71714478	-0.06925222
C	-0.95065146	-0.21494057	-0.65391888
C	0.54576474	-1.44109889	0.57426803
C	-0.54870487	-1.56145136	-0.24638042
C	-0.13505012	2.19524612	-0.22836166
H	-1.77692023	0.01900766	-1.30650027
H	1.66892624	0.38923332	1.27225625
H	-1.03675559	-2.47467906	-0.54600598
H	1.08846681	-2.24065238	1.05185306
C	1.13757782	2.91997135	-0.57160687
H	1.78211894	3.03137948	0.30827210
H	1.70713955	2.37975008	-1.32744068
H	0.92673287	3.92233740	-0.94559403
C	-1.18544544	2.94440997	0.54719904
H	-0.90465844	3.03036052	1.60383205
H	-1.31633022	3.95481051	0.15829150
H	-2.14593817	2.43146803	0.50881596

$(S_0/S_1)_{CI}^{0^\circ}$

C	1.07557205	-0.17552020	0.05261043
C	-0.02235546	0.64801582	0.04957548
C	-1.12836100	-0.16451233	0.05957887
C	0.62521577	-1.64426111	0.06573425
C	-0.69255561	-1.63769020	0.06936663
C	-0.01463252	2.25152993	0.03679685
H	-2.16041510	0.14774511	0.06471170
H	2.11068228	0.12649723	0.05222749
H	-1.37233618	-2.47453738	0.07339258
H	1.29663696	-2.48783333	0.06496801
C	1.27960731	3.00531245	0.02665791
H	1.39145629	3.62644770	0.92323780
H	2.14692547	2.35144657	-0.01893851
H	1.33956219	3.68183019	-0.83322204

C	-1.30149723	3.01794360	0.03482097
H	-1.39139439	3.65537136	0.92224514
H	-1.37013464	3.68027887	-0.83554034
H	-2.17579285	2.37213555	0.01580272

$(S_0/S_1)_{CI}^{10^\circ}$

C	1.06500852	-0.12366398	0.24422908
C	-0.02184805	0.69991040	0.04711313
C	-1.11543409	-0.11640672	-0.14296591
C	0.62008333	-1.58243183	0.17255093
C	-0.68312953	-1.57805850	-0.05528167
C	-0.01532257	2.27839049	0.04034828
H	-2.12977084	0.20243744	-0.32371085
H	2.08215383	0.18834940	0.42105970
H	-1.34788742	-2.41974588	-0.16337900
H	1.27762516	-2.42859068	0.28958255
C	1.30905382	2.96511509	0.03898386
H	1.88188302	2.74795921	0.94829228
H	1.93118717	2.64486753	-0.80486656
H	1.19146928	4.04606408	-0.02506044
C	-1.33398936	2.97594575	0.03578011
H	-1.20772538	4.05622064	0.09449424
H	-1.90713315	2.75879035	-0.87335743
H	-1.96004039	2.66504704	0.88021362

$(S_0/S_1)_{CI}^{20^\circ}$

C	1.05126365	-0.12421947	0.30991172
C	-0.02302586	0.69786122	0.05400808
C	-1.10547407	-0.11716019	-0.18865275
C	0.60810530	-1.57865424	0.22091630
C	-0.66847288	-1.57469052	-0.11374811
C	-0.01559252	2.27023152	0.04119686
H	-2.11268506	0.20285497	-0.40384760
H	2.06009754	0.18922764	0.52720131
H	-1.32146059	-2.41892693	-0.26220707
H	1.25716527	-2.42691713	0.36346038
C	1.30681379	2.96224952	-0.00756267
H	1.91111911	2.76331370	0.88355652
H	1.90325538	2.63277153	-0.86668091
H	1.18007815	4.04105536	-0.08953335
C	-1.33149258	2.97511656	0.07857801
H	-1.19515477	4.05383851	0.14471441
H	-1.93732655	2.76834921	-0.80966613
H	-1.93102994	2.66389857	0.94238091

$(S_0/S_1)_{CI}^{30^\circ}$

C	1.00900125	-0.12080867	0.46020753
C	-0.02045900	0.70736574	0.04673156
C	-1.05699306	-0.11419905	-0.36137906
C	0.58722814	-1.56728885	0.29965690
C	-0.65174314	-1.56346744	-0.18405906
C	-0.01520982	2.25929714	0.04112587
H	-2.01251342	0.20592195	-0.74598983
H	1.96997018	0.19228123	0.83705837
H	-1.28052820	-2.40852629	-0.41296487
H	1.20642502	-2.41649000	0.53930322
C	1.29220453	2.95860577	-0.15212564
H	1.95100035	2.83679158	0.71507972
H	1.83445326	2.55789670	-1.01519923
H	1.15355831	4.02768780	-0.30956147
C	-1.31756237	2.96816433	0.22924726
H	-1.17298613	4.03848102	0.37224875
H	-1.98037526	2.83801372	-0.63387080
H	-1.85928727	2.58047316	1.09851667

$(S_0/S_1)_{CI}^{40^\circ}$

C	0.96337111	-0.11772228	0.56630115
C	-0.02170463	0.71200190	0.04507942
C	-1.01293762	-0.11531680	-0.47053125
C	0.56420987	-1.55423750	0.36455803
C	-0.62471612	-1.55205642	-0.24868755
C	-0.01544329	2.24200439	0.04019687
H	-1.92630601	0.20457038	-0.94710356
H	1.87894675	0.19947820	1.04012194
H	-1.22080744	-2.40203336	-0.53849450
H	1.14991880	-2.40622120	0.66976187
C	1.27381135	2.94993387	-0.24294320
H	1.99281430	2.82391504	0.57371309
H	1.75556758	2.56132645	-1.14577171
H	1.11846123	4.01985808	-0.37628151
C	-1.30008888	2.96247817	0.31904861
H	-1.13366133	4.03138196	0.44904088
H	-2.01965279	2.83950199	-0.49724838
H	-1.78559949	2.58133697	1.22326569

$(S_0/S_1)^{50^\circ}_{CI}$

C	0.91402461	-0.11797091	0.66401455
C	-0.02114313	0.71696168	0.04634046
C	-0.96228127	-0.11667611	-0.56439051
C	0.53510423	-1.53675663	0.42516703
C	-0.59805672	-1.53558130	-0.30664869
C	-0.01545066	2.22561654	0.04025578
H	-1.81615061	0.20425410	-1.13991806
H	1.77118345	0.20165432	1.23528319
H	-1.15360493	-2.38978367	-0.65817904
H	1.08205874	-2.39196143	0.78760008
C	1.24527316	2.94362600	-0.34216739
H	2.00127629	2.87825981	0.44778227
H	1.69105733	2.50906570	-1.24098237
H	1.06173214	4.00092873	-0.53192057
C	-1.27107783	2.95522001	0.41695075
H	-1.07766195	4.00980858	0.61136930
H	-2.02293495	2.90073265	-0.37787500
H	-1.72716450	2.52280178	1.31134412

$(S_0/S_1)^{60^\circ}_{CI}$

C	0.85355657	-0.12187952	0.75905777
C	-0.02167221	0.71902453	0.04716916
C	-0.90353455	-0.12043502	-0.65801903
C	0.50110364	-1.52052218	0.48483985
C	-0.56322530	-1.51957533	-0.37170289
C	-0.01574278	2.20820163	0.04127216
H	-1.70139449	0.20376492	-1.30719154
H	1.65364623	0.20112383	1.40608136
H	-1.07456796	-2.37899972	-0.77403854
H	1.00505510	-2.38080107	0.89459819
C	1.21016957	2.93600453	-0.43389003
H	1.99515306	2.94403766	0.33030214
H	1.63400163	2.45845460	-1.31958805
H	0.98574813	3.97380406	-0.68003070
C	-1.23557005	2.94975115	0.51052526
H	-1.00462037	3.99001876	0.73942983
H	-2.02388983	2.94990420	-0.25023038
H	-1.65803297	2.48832281	1.40544135

$(S_0/S_1)^{70^\circ}_{CI}$

C	0.78504312	-0.12841828	0.84707874
C	-0.02242445	0.71660920	0.04787204
C	-0.83763360	-0.12801462	-0.74368957
C	0.46248957	-1.50595444	0.54328912
C	-0.52350450	-1.50578414	-0.43158642
C	-0.01551396	2.19437482	0.04132261

H	-1.56841049	0.19937324	-1.46591252
H	1.51815845	0.19892039	1.56695930
H	-0.98003407	-2.37036474	-0.88553213
H	0.91276282	-2.37064085	1.00322814
C	1.16711270	2.93100691	-0.52613425
H	1.98103566	3.01357477	0.20294220
H	1.56978171	2.41661304	-1.39936353
H	0.89308927	3.94417108	-0.82143714
C	-1.19184941	2.94568155	0.60250400
H	-0.91311948	3.96334284	0.87689955
H	-2.01045640	3.01657139	-0.12247348
H	-1.59034350	2.44913769	1.48805927

$(S_0/S_1)^{80^\circ}_{CI}$

C	0.70811741	-0.13604320	0.92601380
C	-0.02241595	0.71101634	0.04797153
C	-0.76090534	-0.13658998	-0.82272383
C	0.41941408	-1.49786715	0.59585323
C	-0.48046584	-1.49829281	-0.48471326
C	-0.01554098	2.18844766	0.04149343
H	-1.41766105	0.19427042	-1.61137567
H	1.36746842	0.19536804	1.71226940
H	-0.88535081	-2.36691381	-0.97819972
H	0.81802880	-2.36605020	1.09517932
C	1.11758473	2.93006847	-0.61482042
H	1.95407527	3.08628748	0.07574648
H	1.50499626	2.38074529	-1.47288368
H	0.79304196	3.91383318	-0.95651903
C	-1.14228813	2.94513137	0.69158077
H	-0.81477165	3.93619205	1.00814917
H	-1.98534440	3.08541013	0.00563591
H	-1.52179933	2.41518657	1.56536849

$(S_0/S_1)^{90^\circ}_{CI}$

C	0.61993819	-0.13861859	0.99462739
C	-0.02154564	0.70828736	0.04711464
C	-0.67021038	-0.14066502	-0.89368666
C	0.36759038	-1.49617608	0.63906312
C	-0.43075046	-1.49742266	-0.52637571
C	-0.01527990	2.18843221	0.04125430
H	-1.24851264	0.19095404	-1.74123466
H	1.20126999	0.19482287	1.83938247
H	-0.79670795	-2.36768173	-1.04663874
H	0.72543707	-2.36534102	1.16674396
C	1.06409312	2.93212465	-0.69699675
H	1.92656873	3.12714869	-0.04989411
H	1.42452307	2.36241554	-1.55361185
H	0.70364246	3.89781351	-1.05526226
C	-1.08887572	2.94582714	0.77395644
H	-0.72687098	3.91944861	1.10806034
H	-1.95818442	3.12501707	0.13146750
H	-1.43994146	2.39381327	1.64605650

$(S_0/S_1)^{100^\circ}_{CI}$

C	0.52354012	-0.13588034	1.05010596
C	-0.02063037	0.70942071	0.04669433
C	-0.57126698	-0.13935813	-0.95025886
C	0.30696731	-1.50287804	0.66913500
C	-0.37207198	-1.50493678	-0.55503825
C	-0.01502454	2.18630585	0.04100674
H	-1.06378486	0.18935048	-1.85154037
H	1.01950989	0.19592284	1.94835257
H	-0.69621519	-2.37304238	-1.10575938
H	0.62147384	-2.36925056	1.22810921
C	1.00280316	2.93466327	-0.77479852
H	1.89016903	3.16626345	-0.17534110

H	1.33496085	2.34945617	-1.63216260
H	0.60299555	3.88242885	-1.13995335
C	-1.02779207	2.94717890	0.85153520
H	-0.62731765	3.90247185	1.19544043
H	-1.92120533	3.16489384	0.25588194
H	-1.35092735	2.37718987	1.72261697

$(S_0/S_1)_{CI}^{110^\circ}$

C	0.41945212	-0.13480452	1.09283786
C	-0.02164808	0.70615338	0.04709362
C	-0.46980866	-0.13968022	-0.99190517
C	0.23951841	-1.52160419	0.69015016
C	-0.30267015	-1.52442511	-0.57714521
C	-0.01541449	2.18651572	0.04126103
H	-0.87555160	0.18485046	-1.93714369
H	0.82810996	0.19406202	2.03530032
H	-0.56680922	-2.38846341	-1.16504928
H	0.49591403	-2.38299587	1.28534675
C	0.93464326	2.94003285	-0.84780636
H	1.83856481	3.23225948	-0.30126052
H	1.24966339	2.33793015	-1.69907540
H	0.47838623	3.85678190	-1.22714276
C	-0.95943759	2.95414534	0.92468202
H	-0.50203138	3.87858637	1.28311135
H	-1.86913458	3.23387860	0.38114722
H	-1.26556301	2.36697690	1.78962398

$(S_0/S_1)_{CI}^{120^\circ}$

C	0.31496060	-0.13839191	1.12266342
C	-0.02259457	0.69674277	0.04764886
C	-0.36883216	-0.14344835	-1.02127267
C	0.17229284	-1.54741992	0.70334971
C	-0.23452029	-1.55041792	-0.59293306
C	-0.01502671	2.19273213	0.04162747
H	-0.68735715	0.17785764	-2.00062125
H	0.63540770	0.18690403	2.10003929
H	-0.43142245	-2.40941534	-1.21366239
H	0.36068776	-2.40354392	1.33072707
C	0.85943119	2.95024995	-0.91786237
H	1.74995454	3.35339882	-0.42179544
H	1.19891507	2.31946135	-1.73756268
H	0.32180302	3.80071793	-1.34455158
C	-0.88231820	2.96535419	0.99558925
H	-0.34834173	3.83317764	1.39004647
H	-1.78684523	3.34366512	0.50510380
H	-1.20001077	2.35257563	1.83749201

$(S_0/S_1)_{CI}^{130^\circ}$

C	0.28354619	-0.12231541	1.12753211
C	-0.02383968	0.71031398	0.04784758
C	-0.34049237	-0.12446251	-1.02719021
C	0.15127200	-1.54275884	0.70201399
C	-0.21559443	-1.54399659	-0.59620412
C	-0.01552091	2.21820287	0.04343299
H	-0.63187235	0.19537167	-2.01531384
H	0.57726089	0.19987450	2.11425681
H	-0.39068033	-2.39923545	-1.22856731
H	0.32135100	-2.39665937	1.33754518
C	0.71773625	2.94436684	-1.04603061
H	0.95973348	3.96623151	-0.75206272
H	1.64808728	2.43531334	-1.30665067
H	0.11876316	3.00340087	-1.96195506
C	-0.73966346	2.96205875	1.12709476
H	-0.11342190	3.08230197	2.01874292
H	-1.02971284	3.96163421	0.80074877
H	-1.64076853	2.43055750	1.43878531

$(S_0/S_1)_{CI}^{140^\circ}$

C	0.18416989	-0.11468467	1.14678911
C	-0.02309068	0.70975930	0.04718338
C	-0.23751424	-0.12242759	-1.04510737
C	0.09341716	-1.55188334	0.71366886
C	-0.15057638	-1.55672634	-0.60172282
C	-0.01635451	2.24142119	0.04100974
H	-0.43401476	0.19289166	-2.05783659
H	0.38256065	0.20754710	2.15698037
H	-0.26339258	-2.40964592	-1.25118363
H	0.20461522	-2.40020514	1.36939621
C	0.62656790	2.95099279	-1.11082055
H	0.79353219	4.00449438	-0.88708536
H	1.58999123	2.50130734	-1.36818253
H	0.00569882	2.90325376	-2.01253840
C	-0.65347693	2.96429378	1.18796388
H	-0.02848437	2.92700077	2.08737561
H	-0.82177165	4.01518935	0.95329043
H	-1.61569352	2.51762143	1.45484553

$(S_0/S_1)_{CI}^{150^\circ}$

C	0.07229091	-0.11521328	1.15780635
C	-0.02300197	0.70508282	0.04719320
C	-0.12495320	-0.12327513	-1.05691264
C	0.02682331	-1.56501485	0.71867851
C	-0.08358446	-1.56979836	-0.60675686
C	-0.01669406	2.25713582	0.04151486
H	-0.21610016	0.18975335	-2.08513481
H	0.16607521	0.20555547	2.18341692
H	-0.13025140	-2.41911302	-1.26898311
H	0.07137258	-2.40961362	1.38707093
C	0.54124440	2.95713814	-1.15626777
H	0.69480566	4.01822105	-0.96147194
H	1.50103124	2.52674480	-1.45918318
H	-0.12589479	2.87602685	-2.02237910
C	-0.56874294	2.97129692	1.23381771
H	0.10401374	2.90379182	2.09682628
H	-0.72710629	4.02933307	1.02633648
H	-1.52514431	2.54214802	1.54845404

$(S_0/S_1)_{CI}^{160^\circ}$

C	-0.03462154	-0.11519703	1.15959856
C	-0.02233476	0.70216398	0.04753424
C	-0.01562798	-0.12391415	-1.05807227
C	-0.03574321	-1.57288774	0.71908349
C	-0.01864395	-1.57806417	-0.60633202
C	-0.01700863	2.27055274	0.04124456
H	-0.00265895	0.18788609	-2.09049727
H	-0.04586309	0.20476902	2.18957136
H	-0.00569756	-2.42584017	-1.27203356
H	-0.05250625	-2.41535954	1.39136454
C	0.44752341	2.96139088	-1.19826926
H	0.52614941	4.03705600	-1.04526937
H	1.42797846	2.59439728	-1.52159594
H	-0.23887895	2.79798021	-2.03736261
C	-0.47676734	2.97482812	1.27506825
H	0.21119429	2.81786199	2.11416227
H	-0.55296781	4.04914636	1.11170483
H	-1.45734209	2.61343002	1.60412606

$(S_0/S_1)_{CI}^{170^\circ}$

C	-0.14384159	-0.11450053	1.15156224
C	-0.02143261	0.69987366	0.04706898
C	0.09658839	-0.12487099	-1.05023080
C	-0.09934183	-1.57644234	0.71465060

C	0.04529091	-1.58276562	-0.60011517
C	-0.01725493	2.27852590	0.04058533
H	0.21268175	0.18583272	-2.07657322
H	-0.25999775	0.20589973	2.17486278
H	0.12232316	-2.42976098	-1.26244501
H	-0.17797159	-2.41708330	1.38485633
C	0.35398498	2.96465133	-1.23112401
H	0.39074560	4.04520789	-1.09877093
H	1.33540451	2.63829187	-1.59446323
H	-0.36157612	2.75300603	-2.03435015
C	-0.38495184	2.97571001	1.30701764
H	0.32572242	2.76024407	2.11353644
H	-0.40902371	4.05587059	1.16875113
H	-1.37116627	2.66250985	1.66920690

$(S_0/S_1)_{CI}^{180^\circ}$

C	-0.25449109	-0.11468987	1.13309148
C	-0.02027913	0.69933438	0.04727059
C	0.20995022	-0.12538909	-1.03141586
C	-0.16459359	-1.57877622	0.70392922
C	0.10705360	-1.58502695	-0.59035570
C	-0.01655502	2.28139767	0.04094378
H	0.43262900	0.18480652	-2.04008755
H	-0.47107414	0.20543899	2.13992248
H	0.24485486	-2.43149900	-1.24350808
H	-0.31469812	-2.41874839	1.36272843
C	0.26247966	2.96578022	-1.25466884
H	0.25931643	4.04841423	-1.13561175
H	1.23925621	2.67917188	-1.66220656
H	-0.48094403	2.71130538	-2.01928203
C	-0.29233469	2.97694658	1.33100667
H	0.45943400	2.73873367	2.09301207
H	-0.30105901	4.05825366	1.20033046
H	-1.26276167	2.68474623	1.74892702

6.3.2 XMS-CASPT2

$(S_0)_{min}$

C	1.15611651	-0.15789462	0.04824223
C	-0.02204773	0.73355791	0.04824648
C	-1.20892809	-0.14628904	0.05754810
C	0.70490271	-1.45937069	0.05664232
C	-0.77045792	-1.45212775	0.06249016
C	-0.01535063	2.10367791	0.04049784
H	-2.24641602	0.18252389	0.06004607
H	2.19678673	0.16066367	0.04257725
H	-1.40084689	-2.34386033	0.06952772
H	1.32653173	-2.35725612	0.05875009
C	1.25592912	2.91045428	0.03110138
H	1.28422797	3.57063473	0.91504357
H	2.15308623	2.28355824	0.02894377
H	1.27534661	3.56406850	-0.85791460
C	-1.27864957	2.92292014	0.04070423
H	-1.29245202	3.58548662	0.92318447
H	-1.29967497	3.57454672	-0.84975508
H	-2.18192041	2.30490578	0.04814993

$(S_1)_{min}$

C	1.10268498	-0.15753105	0.04837884
C	-0.02220350	0.70360331	0.04781350
C	-1.15547205	-0.14642465	0.05682719
C	0.65771981	-1.56115871	0.05789503
C	-0.72427585	-1.55436131	0.06308800
C	-0.01496724	2.18145723	0.03918705
H	-2.19881919	0.17198876	0.05904360

H	2.14910133	0.15060503	0.04288321
H	-1.39329673	-2.41561443	0.07029047
H	1.31827183	-2.42895127	0.06014720
C	1.26755412	2.95375420	0.03063034
H	1.33552945	3.61725487	0.91490451
H	2.15185762	2.30463742	0.02782016
H	1.32634404	3.61252753	-0.85774274
C	-1.28985621	2.96633522	0.04053035
H	-1.34283366	3.63289629	0.92349334
H	-1.35066888	3.62321728	-0.84914014
H	-2.18048651	2.32596413	0.04797603

$(S_0/S_1)_{MECI}$

C	0.80834107	-0.01687914	0.76200884
C	-0.08595231	0.73145588	-0.05440462
C	-0.90944666	-0.20933834	-0.70834106
C	0.52499004	-1.45443913	0.60571438
C	-0.51926729	-1.57526174	-0.28915327
C	-0.13444034	2.20041156	-0.19188417
H	-1.69782687	0.03112719	-1.42536774
H	1.58156018	0.40648681	1.40720804
H	-0.98537028	-2.49710651	-0.63984114
H	1.04978972	-2.25968341	1.12137440
C	1.10955368	2.91392268	-0.63385586
H	1.81325879	3.04275331	0.21633708
H	1.64019530	2.34203704	-1.41110740
H	0.87972453	3.92210763	-1.01492662
C	-1.16198703	2.95410148	0.60295872
H	-0.84221350	3.04946268	1.66305290
H	-1.30647031	3.97326598	0.20964982
H	-2.12825535	2.42577587	0.60460363

$(S_0/S_1)_{CI}^{0°}$

C	1.05895191	-0.18158737	0.05161325
C	-0.02213146	0.66178450	0.04869047
C	-1.11151105	-0.17056725	0.05892579
C	0.62544390	-1.69409221	0.06494142
C	-0.69337623	-1.68745982	0.06651830
C	-0.01439485	2.30488557	0.03579106
H	-2.16012088	0.14059767	0.07123560
H	2.11071621	0.11895468	0.05814409
H	-1.40084172	-2.51639702	0.06806429
H	1.32442063	-2.53020680	0.06159743
C	1.29256120	3.02758368	0.02567679
H	1.43077036	3.66289983	0.92644677
H	2.15041865	2.34076372	-0.00749243
H	1.39055002	3.70528049	-0.84751779
C	-1.31432717	3.04021820	0.03446441
H	-1.43152645	3.68906012	0.92843668
H	-1.42020515	3.70691370	-0.84634356
H	-2.17921458	2.36156814	0.02483338

$(S_0/S_1)_{CI}^{10°}$

C	1.05237330	-0.11779467	0.23819902
C	-0.02149614	0.72278568	0.04662577
C	-1.10264095	-0.10998849	-0.13845713
C	0.62075342	-1.61834160	0.16972746
C	-0.68520635	-1.61346703	-0.05139114
C	-0.01481234	2.33278274	0.04032591
H	-2.13304480	0.21116960	-0.31728322
H	2.08611747	0.19580277	0.41111432
H	-1.37652654	-2.44901290	-0.15984410
H	1.30437544	-2.45901644	0.28706929
C	1.32173657	2.97534133	0.03578929
H	1.91027738	2.73267624	0.94810305
H	1.94478282	2.63722910	-0.82153844

H	1.23735434	4.07177283	-0.02071354
C	-1.34616692	2.98596084	0.03979265
H	-1.25333138	4.08181116	0.09432164
H	-1.93427315	2.74641765	-0.87372843
H	-1.97408881	2.65407101	0.89591356

$(S_0/S_1)_{CI}^{20^\circ}$

C	1.02963193	-0.11745376	0.34603456
C	-0.02147554	0.72323500	0.04511798
C	-1.07869956	-0.11087572	-0.25154116
C	0.60791065	-1.61454777	0.23186344
C	-0.67216767	-1.61057228	-0.11706928
C	-0.01535792	2.32221088	0.04113231
H	-2.08606963	0.21000649	-0.53287813
H	2.03977135	0.19687776	0.62479392
H	-1.34871229	-2.44808104	-0.28656133
H	1.27663416	-2.45652562	0.41037954
C	1.31595102	2.97324569	-0.05751186
H	1.94541917	2.78836509	0.84034812
H	1.90261936	2.58733320	-0.91962927
H	1.22194414	4.06441243	-0.17264171
C	-1.34112078	2.98511175	0.13646341
H	-1.23689716	4.07769178	0.22610484
H	-1.97863932	2.78568982	-0.75216409
H	-1.92455854	2.62407612	1.01178468

$(S_0/S_1)_{CI}^{30^\circ}$

C	0.99784108	-0.11684321	0.45260449
C	-0.01884475	0.72533936	0.04401599
C	-1.04420570	-0.10991155	-0.36012308
C	0.58779255	-1.60605973	0.29643228
C	-0.65672801	-1.60067270	-0.17102274
C	-0.01288616	2.30588890	0.03894402
H	-2.01551040	0.20993409	-0.74783259
H	1.97315296	0.19719784	0.83532585
H	-1.31478255	-2.43979950	-0.39644902
H	1.23355198	-2.45039309	0.53695737
C	1.30340209	2.97236651	-0.15481711
H	1.98133469	2.82064721	0.71236948
H	1.84422078	2.56711217	-1.03636186
H	1.19113599	4.05878811	-0.29476506
C	-1.32680951	2.97832325	0.22879990
H	-1.21115829	4.06302589	0.37908738
H	-2.00152996	2.83784426	-0.64280468
H	-1.87379343	2.56741202	1.10366535

$(S_0/S_1)_{CI}^{40^\circ}$

C	0.95625274	-0.11487396	0.55641985
C	-0.01863011	0.72900263	0.04382402
C	-1.00108973	-0.10866211	-0.46588137
C	0.56432289	-1.59220169	0.35774131
C	-0.63402455	-1.58726184	-0.23378760
C	-0.01377460	2.28506263	0.04002393
H	-1.92055325	0.21246266	-0.96369230
H	1.88511854	0.20153837	1.03958846
H	-1.26459463	-2.43008139	-0.51714939
H	1.17957922	-2.43994732	0.65929526
C	1.28174908	2.96563171	-0.24655380
H	2.00568910	2.85024585	0.58752642
H	1.77828857	2.53480754	-1.14051226
H	1.14800669	4.04565065	-0.41559954
C	-1.30661562	2.97233359	0.32361050
H	-1.16812316	4.05317968	0.48321390
H	-2.03346283	2.85254872	-0.50691141
H	-1.80195499	2.55076412	1.22286998

$(S_0/S_1)_{CI}^{50^\circ}$

C	0.90615662	-0.11131366	0.65884491
C	-0.01903814	0.73386717	0.04487840
C	-0.95098040	-0.10854139	-0.56392449
C	0.53374247	-1.57136282	0.42195556
C	-0.60454431	-1.56894177	-0.29561221
C	-0.01395311	2.25990897	0.03953981
H	-1.81149422	0.21534295	-1.15584699
H	1.77549902	0.20971585	1.23930083
H	-1.19222538	-2.41977643	-0.64096111
H	1.10935048	-2.42475684	0.78130883
C	1.25350978	2.95571537	-0.34098692
H	2.02702526	2.85410326	0.44787825
H	1.69585358	2.52148873	-1.25957597
H	1.09504169	4.03270915	-0.50668091
C	-1.27819283	2.96371691	0.41560077
H	-1.11345922	4.03929325	0.58452183
H	-2.04883819	2.86932305	-0.37713280
H	-1.72726974	2.52970809	1.33091815

$(S_0/S_1)_{CI}^{60^\circ}$

C	0.84864878	-0.11097021	0.75575349
C	-0.02024900	0.73659944	0.04615870
C	-0.89556549	-0.11086741	-0.65691831
C	0.50036558	-1.54746033	0.48360071
C	-0.56569175	-1.54724395	-0.36260567
C	-0.01513946	2.23345366	0.03995076
H	-1.69661625	0.21718914	-1.32444659
H	1.65405715	0.21645629	1.41833781
H	-1.10263550	-2.40685755	-0.76508469
H	1.02598315	-2.40772905	0.89946874
C	1.21556566	2.94375478	-0.43438207
H	2.02571079	2.89703759	0.32205024
H	1.62089544	2.47461815	-1.35016188
H	1.02006106	4.00767630	-0.64034837
C	-1.24092203	2.95650513	0.50832758
H	-1.03516200	4.01811153	0.71657891
H	-2.04697282	2.91979158	-0.25312304
H	-1.65614995	2.49013475	1.42086963

$(S_0/S_1)_{CI}^{70^\circ}$

C	0.78429601	-0.11388875	0.84805261
C	-0.02218744	0.73454469	0.04807360
C	-0.83486898	-0.11748554	-0.74272026
C	0.46248903	-1.52243258	0.54453495
C	-0.52208004	-1.52441975	-0.42443542
C	-0.01681105	2.20820309	0.04011840
H	-1.56848338	0.21545214	-1.48076099
H	1.51947637	0.22204388	1.58315675
H	-0.99155411	-2.39404262	-0.88624269
H	0.92616584	-2.39004279	1.01588781
C	1.16902286	2.93177202	-0.52831814
H	2.01359943	2.95067231	0.19063017
H	1.54103817	2.42749821	-1.43671337
H	0.92421749	3.97699213	-0.77435886
C	-1.19569873	2.94956914	0.59988263
H	-0.93212557	3.98630512	0.86251017
H	-2.02870289	2.99712570	-0.13135264
H	-1.59160966	2.44233343	1.49608122

$(S_0/S_1)_{CI}^{80^\circ}$

C	0.70997538	-0.12187740	0.93201647
C	-0.02373071	0.72813088	0.04907651
C	-0.76405270	-0.12718860	-0.82321939
C	0.41944998	-1.50548683	0.60108279

C	-0.47561218	-1.50848021	-0.48262291
C	-0.01764016	2.19358966	0.04041216
H	-1.42433349	0.21083968	-1.62477403
H	1.37146252	0.22089274	1.73054124
H	-0.87837165	-2.38608043	-0.99062390
H	0.81957385	-2.38002437	1.11636610
C	1.11796557	2.92495265	-0.61841067
H	1.98856581	3.02423432	0.06203924
H	1.47022365	2.38408025	-1.51164643
H	0.81583235	3.94293985	-0.91202718
C	-1.14525967	2.94577319	0.68941165
H	-0.82115099	3.95241993	0.99903421
H	-2.00181214	3.07697065	-0.00342225
H	-1.52490210	2.40451388	1.57079230

$(S_0/S_1)_{CI}^{90^\circ}$

C	0.62166291	-0.12476012	1.00299103
C	-0.02121791	0.72608131	0.04773031
C	-0.67008407	-0.12994975	-0.89871135
C	0.36863075	-1.49982820	0.64467177
C	-0.43066606	-1.50298864	-0.52354879
C	-0.01616633	2.19424705	0.04000222
H	-1.25221421	0.20972011	-1.75763312
H	1.20680909	0.21965617	1.85795745
H	-0.80585681	-2.38312793	-1.04808195
H	0.73587950	-2.37716266	1.17943079
C	1.06782902	2.92708363	-0.69845675
H	1.96617111	3.05696319	-0.06112784
H	1.38739052	2.36685092	-1.59233560
H	0.73597640	3.93204370	-1.00583526
C	-1.09429618	2.94379927	0.77013863
H	-0.74631362	3.94026367	1.08773985
H	-1.98181271	3.09664382	0.12269185
H	-1.43553807	2.38466429	1.65640265

$(S_0/S_1)_{CI}^{100^\circ}$

C	0.52133479	-0.12240504	1.05555156
C	-0.02073450	0.72723175	0.04738108
C	-0.56843597	-0.12896458	-0.95155029
C	0.30508232	-1.51406967	0.67325199
C	-0.36889944	-1.51827299	-0.55059816
C	-0.01578835	2.19489311	0.03961683
H	-1.06672866	0.20616299	-1.86381341
H	1.02378394	0.21893152	1.96320589
H	-0.70184810	-2.39369252	-1.11019700
H	0.62979882	-2.38584756	1.24329983
C	1.00701310	2.93281786	-0.77588171
H	1.93379108	3.09403507	-0.18846348
H	1.29100829	2.35772602	-1.67210752
H	0.64005328	3.92464482	-1.08713553
C	-1.03348991	2.94859416	0.84716629
H	-0.65027058	3.93060184	1.17038343
H	-1.94851966	3.13444842	0.24864014
H	-1.34096713	2.37336461	1.73527594

$(S_0/S_1)_{CI}^{110^\circ}$

C	0.41691754	-0.12588554	1.09325619
C	-0.02247803	0.71922333	0.04877782
C	-0.46796156	-0.13381592	-0.98588041
C	0.23809467	-1.54686740	0.69325320
C	-0.29514701	-1.55189019	-0.57180594
C	-0.01724841	2.19950725	0.04003640
H	-0.88536210	0.19367292	-1.94106556
H	0.83487046	0.20939976	2.04550344
H	-0.55659602	-2.41859616	-1.18027813
H	0.49517557	-2.40875407	1.31034273

C	0.93533302	2.94174644	-0.85188754
H	1.87203672	3.20883538	-0.31955978
H	1.22040651	2.33547624	-1.72580871
H	0.49026418	3.88671223	-1.20714635
C	-0.96330497	2.96159009	0.92194617
H	-0.49535641	3.88830443	1.29618845
H	-1.87972453	3.26584152	0.37460421
H	-1.28373632	2.35569950	1.78354970

$(S_0/S_1)_{CI}^{120^\circ}$

C	0.36529330	-0.12436808	1.10627248
C	-0.02377402	0.71530910	0.04946673
C	-0.42098215	-0.13806022	-0.99348693
C	0.20319938	-1.56578531	0.70364471
C	-0.24913624	-1.57468808	-0.57774032
C	-0.01671763	2.21286075	0.03683119
H	-0.80517846	0.18351688	-1.96532800
H	0.74641639	0.20966335	2.07521613
H	-0.47048688	-2.43753279	-1.20691604
H	0.42224716	-2.42008286	1.34491490
C	0.81118759	2.95248671	-0.97051144
H	1.34690157	3.80814731	-0.51866140
H	1.55069133	2.28790442	-1.44445932
H	0.17469223	3.37005719	-1.77759905
C	-0.84026952	2.97024934	1.03482209
H	-0.21883727	3.31083530	1.88916514
H	-1.29183677	3.87617892	0.59140379
H	-1.64722669	2.34350786	1.44699123

$(S_0/S_1)_{CI}^{130^\circ}$

C	0.28848472	-0.10681166	1.11917372
C	-0.02579158	0.73263621	0.04951881
C	-0.35121282	-0.11565384	-1.01057698
C	0.15267534	-1.56899288	0.70740172
C	-0.20779734	-1.57407016	-0.58978539
C	-0.01594235	2.25878939	0.04011349
H	-0.66711937	0.20331233	-2.00771031
H	0.59923805	0.21993131	2.11540694
H	-0.37682961	-2.42875328	-1.24527036
H	0.32224422	-2.41850999	1.36943362
C	0.71311926	2.94851956	-1.06796091
H	0.88959650	4.01264456	-0.84484585
H	1.68953469	2.46594082	-1.26855367
H	0.14654147	2.90276309	-2.02135268
C	-0.73545397	2.97312231	1.13879057
H	-0.17253416	2.92593944	2.09426609
H	-0.89107524	4.03809082	0.90461024
H	-1.72149447	2.51130178	1.34136683

$(S_0/S_1)_{CI}^{140^\circ}$

C	0.18166206	-0.10627723	1.13704150
C	-0.02629091	0.72917297	0.04926479
C	-0.24421173	-0.11764329	-1.02935572
C	0.08914543	-1.58704963	0.71901970
C	-0.14027912	-1.59334829	-0.59833931
C	-0.01720890	2.28545761	0.04008054
H	-0.45739924	0.19622962	-2.05536746
H	0.38895411	0.21796375	2.16099403
H	-0.23796711	-2.44225262	-1.27522057
H	0.19327127	-2.42944120	1.40305183
C	0.62755927	2.95681558	-1.12573131
H	0.77394838	4.03381233	-0.94778153
H	1.61430940	2.50601851	-1.35888075
H	0.02238354	2.85673970	-2.05123132
C	-0.65291932	2.98149332	1.19586837
H	-0.06318121	2.86021142	2.12855980

H	-0.75631357	4.06276517	1.01427905
H	-1.65927899	2.56953208	1.41777425

$(S_0/S_1)_{CI}^{150^\circ}$

C	0.07029360	-0.10747133	1.14804316
C	-0.02531554	0.72686346	0.04944081
C	-0.13099717	-0.11820135	-1.04006458
C	0.02162345	-1.60013597	0.72423053
C	-0.07854687	-1.60654614	-0.60267119
C	-0.01598787	2.30778520	0.03920033
H	-0.22567380	0.19444895	-2.08390527
H	0.16468146	0.21457475	2.18908582
H	-0.11626881	-2.45164381	-1.29003987
H	0.06245412	-2.43873056	1.41939062
C	0.54426505	2.96404861	-1.17219300
H	0.67805694	4.04706301	-1.02292623
H	1.52808027	2.53088022	-1.45157099
H	-0.10690603	2.83317121	-2.06339901
C	-0.56830184	2.98661392	1.24181170
H	0.07682984	2.85179264	2.13662600
H	-0.68056542	4.07037411	1.08133543
H	-1.56153800	2.57531287	1.52163162

$(S_0/S_1)_{CI}^{160^\circ}$

C	-0.03823300	-0.10746776	1.14854962
C	-0.02310415	0.72382375	0.04853355
C	-0.01529237	-0.11993138	-1.04243302
C	-0.04025331	-1.60781811	0.72467350
C	-0.01329442	-1.61537223	-0.60146591
C	-0.01611031	2.32298369	0.03911308
H	-0.00090083	0.19129514	-2.09109155
H	-0.05618630	0.21611492	2.19342976
H	0.00684260	-2.45883742	-1.29149540
H	-0.06805659	-2.44304924	1.42434491
C	0.45561083	2.96874987	-1.21210680
H	0.54910494	4.05938892	-1.09175437
H	1.44377695	2.57314669	-1.53264434
H	-0.23211135	2.78830842	-2.06732939
C	-0.48179530	2.98900841	1.28180964
H	0.19865402	2.80398719	2.14172914
H	-0.55352298	4.07996919	1.15012129
H	-1.47894505	2.61589974	1.60204217

$(S_0/S_1)_{CI}^{170^\circ}$

C	-0.15085825	-0.10652958	1.13927366
C	-0.02350542	0.72281937	0.04769068
C	0.09648527	-0.12105099	-1.03384637
C	-0.10875859	-1.61268803	0.72033134
C	0.05918625	-1.62137223	-0.59392971
C	-0.01645208	2.33113109	0.03823704
H	0.20942130	0.18949710	-2.07682683
H	-0.27424490	0.21764613	2.17686302
H	0.14463315	-2.46377706	-1.28016446
H	-0.19751476	-2.44607640	1.41711007
C	0.36501509	2.97171990	-1.24429263
H	0.42099372	4.06669693	-1.14258491
H	1.35169539	2.61268868	-1.61119826
H	-0.35724497	2.75006039	-2.06121718
C	-0.39220805	2.98991989	1.31308604
H	0.31581129	2.75026078	2.13702321
H	-0.41336791	4.08552410	1.20509231
H	-1.39290315	2.66372973	1.67337886

$(S_0/S_1)_{CI}^{180^\circ}$

C	-0.26026633	-0.10658202	1.12083571

C	-0.02180385	0.72219174	0.04798795
C	0.20986551	-0.12125950	-1.01485084
C	-0.17358090	-1.61284704	0.70830635
C	0.10994768	-1.62170926	-0.58490360
C	-0.01530054	2.33582481	0.03856202
H	0.43804492	0.18931298	-2.03876202
H	-0.48221470	0.21798101	2.14178537
H	0.25813879	-2.46393274	-1.26057981
H	-0.32852507	-2.44554378	1.39418078
C	0.27418353	2.97222836	-1.26844320
H	0.26989191	4.07027905	-1.18653997
H	1.26561117	2.66892583	-1.67215838
H	-0.46636480	2.68852019	-2.04896576
C	-0.29956090	2.99006197	1.33792745
H	0.44328174	2.71731444	2.12026114
H	-0.29534461	4.08687200	1.24080718
H	-1.28982018	2.69256172	1.74857551

6.3.3 MS-CASPT2

$(S_0)_{min}$

C	1.15548123	-0.15755290	0.04811856
C	-0.02205405	0.73347054	0.04798452
C	-1.20826813	-0.14592802	0.05734474
C	0.70487950	-1.45977882	0.05676120
C	-0.77044390	-1.45252251	0.06253001
C	-0.01534757	2.10321066	0.04040239
H	-2.24544273	0.18246484	0.05969515
H	2.19582578	0.16061288	0.04241826
H	-1.40138804	-2.34355323	0.06969174
H	1.32706677	-2.35697166	0.05906009
C	1.25588067	2.91026996	0.03109576
H	1.28462137	3.57060182	0.91487606
H	2.15337746	2.28397228	0.02903252
H	1.27589592	3.56396065	-0.85782025
C	-1.27860760	2.92273570	0.04078751
H	-1.29283638	3.58531459	0.92322235
H	-1.30025378	3.57458126	-0.84945959
H	-2.18220317	2.30531181	0.04828488

$(S_1)_{min}$

C	1.10226091	-0.15759600	0.04844058
C	-0.02221337	0.70182941	0.04761105
C	-1.15504940	-0.14649555	0.05667198
C	0.65760525	-1.56193911	0.05805697
C	-0.72416829	-1.55514427	0.06308290
C	-0.01496530	2.18220224	0.03879856
H	-2.19807005	0.17258739	0.05866446
H	2.14835657	0.15121461	0.04297780
H	-1.39293573	-2.41631869	0.07051191
H	1.31790729	-2.42964927	0.06064885
C	1.26726588	2.95402673	0.03048671
H	1.33516804	3.61716233	0.91507053
H	2.15151589	2.30502827	0.02719947
H	1.32568396	3.61342227	-0.85747230
C	-1.28956578	2.96660639	0.04054741
H	-1.34226209	3.63294302	0.92372600
H	-1.35020609	3.62396727	-0.84880517
H	-2.18014432	2.32635282	0.04780824

$(S_0/S_1)_{MECI}$

C	0.81184586	-0.01751919	0.75980544
C	-0.08475277	0.73102566	-0.05305533
C	-0.91210645	-0.20852281	-0.70522284
C	0.52676100	-1.45383102	0.60423957

C	-0.52126925	-1.57415955	-0.28862888
C	-0.13291324	2.19994406	-0.19021656
H	-1.70208758	0.03378030	-1.41976009
H	1.58710759	0.40560119	1.40272003
H	-0.98825312	-2.49593842	-0.63762345
H	1.05165515	-2.26002476	1.11781512
C	1.11092833	2.91375239	-0.63142061
H	1.81325513	3.04394107	0.21965542
H	1.64305728	2.34138796	-1.40725158
H	0.88081622	3.92130610	-1.01383567
C	-1.16359618	2.95338073	0.60042479
H	-0.84707220	3.05046071	1.66127262
H	-1.30804176	3.97177505	0.20523856
H	-2.12915063	2.42384037	0.59986938

$(S_0/S_1)_{CI}^{0^\circ}$

C	1.05942199	-0.17861928	0.05371749
C	-0.02251089	0.66279185	0.04991961
C	-1.11239788	-0.16807641	0.05953057
C	0.62597563	-1.68648935	0.06696594
C	-0.69340586	-1.68038738	0.07216910
C	-0.01469241	2.29638792	0.03682098
H	-2.16072530	0.14504222	0.05827428
H	2.11064258	0.12471195	0.04898555
H	-1.39680642	-2.51239461	0.08398889
H	1.32159250	-2.52507100	0.06745073
C	1.28953608	3.02163550	0.02747359
H	1.43786853	3.63946195	0.93838152
H	2.14878682	2.33897830	-0.03593550
H	1.36846192	3.71819754	-0.83292519
C	-1.31134421	3.03519166	0.03442764
H	-1.43524390	3.67601073	0.93263720
H	-1.40031902	3.71214858	-0.84093060
H	-2.17865679	2.36067966	0.00307416

$(S_0/S_1)_{CI}^{10^\circ}$

C	1.06394704	-0.10641734	0.23012453
C	-0.02916704	0.71799240	0.06853463
C	-1.11375512	-0.12399880	0.00954471
C	0.63965609	-1.60382746	0.27835425
C	-0.67889192	-1.61690677	0.14594374
C	-0.02670255	2.32099391	-0.02907624
H	-2.15875565	0.18381541	-0.09229702
H	2.10315493	0.22233557	0.32511319
H	-1.36832492	-2.46062636	0.13215500
H	1.33376034	-2.43611922	0.39197934
C	1.30730651	2.95362189	-0.16564762
H	1.94141199	2.80843330	0.73753450
H	1.88339809	2.51439990	-1.00902057
H	1.22164954	4.03760222	-0.33875289
C	-1.34424838	3.00263106	0.02640388
H	-1.81516879	2.96507842	1.03537321
H	-1.24869111	4.06282669	-0.25573066
H	-2.07439567	2.53836501	-0.66651001

$(S_0/S_1)_{CI}^{20^\circ}$

C	1.05271488	-0.09648565	0.34831820
C	-0.02011435	0.72441767	0.07324276
C	-1.09836941	-0.12178706	-0.07913215
C	0.62286233	-1.59081131	0.37347671
C	-0.67927984	-1.60515834	0.12179278
C	-0.01514362	2.31179164	-0.04362832
H	-2.12479407	0.18519394	-0.30228560
H	2.08171305	0.23256466	0.52049089
H	-1.36324021	-2.45155751	0.06280222
H	1.30148994	-2.42320436	0.55836544

C	1.29833883	2.95671392	-0.29333695
H	1.99582431	2.82497989	0.56175878
H	1.81335252	2.51440339	-1.17333601
H	1.19112367	4.03840485	-0.46889845
C	-1.32870497	2.98746745	0.10950054
H	-1.85026473	2.66311981	1.03639884
H	-1.21721712	4.08257863	0.14172540
H	-2.02410782	2.74756822	-0.72322910

$(S_0/S_1)_{CI}^{30^\circ}$

C	1.02810923	-0.08617659	0.45664446
C	-0.02191029	0.72580109	0.07122900
C	-1.07421997	-0.13198814	-0.19277250
C	0.61162910	-1.57823425	0.43645564
C	-0.66671759	-1.60320077	0.06450160
C	-0.01746728	2.29565814	-0.04220908
H	-2.07060943	0.16468470	-0.53357974
H	2.03014594	0.25360198	0.73448527
H	-1.33366331	-2.45699722	-0.05310455
H	1.27380424	-2.40725014	0.68525405
C	1.27824913	2.94345251	-0.38358431
H	2.02658118	2.83424029	0.42996471
H	1.74184597	2.48691003	-1.28376100
H	1.15374706	4.02129990	-0.57059824
C	-1.30957068	2.99204633	0.20207889
H	-1.78365405	2.65602731	1.14848286
H	-1.17691758	4.08397506	0.25205865
H	-2.05319828	2.78634960	-0.59751970

$(S_0/S_1)_{CI}^{40^\circ}$

C	0.99618256	-0.08164597	0.56025815
C	-0.01699319	0.72998028	0.06933644
C	-1.03681772	-0.13463549	-0.30324815
C	0.59082771	-1.56024510	0.49421740
C	-0.64819567	-1.59103257	-0.00900680
C	-0.01428036	2.27808900	-0.04142379
H	-1.99262498	0.15897421	-0.74617445
H	1.95729516	0.26491565	0.95058157
H	-1.29610749	-2.44944775	-0.18458530
H	1.21955091	-2.38846189	0.82006206
C	1.25153777	2.93471326	-0.47479666
H	2.03982242	2.87530765	0.30547490
H	1.67496171	2.44029234	-1.37293721
H	1.09975189	4.00031117	-0.70682688
C	-1.28164932	2.98751559	0.29541949
H	-1.69317835	2.63605492	1.26416159
H	-1.13573490	4.07706936	0.35893424
H	-2.07816477	2.80244517	-0.45542059

$(S_0/S_1)_{CI}^{50^\circ}$

C	0.95416191	-0.07007138	0.66826393
C	-0.01809265	0.73561875	0.07153392
C	-0.99512787	-0.14098283	-0.40374078
C	0.56439667	-1.53684329	0.56106675
C	-0.62756620	-1.57804202	-0.06705564
C	-0.01531352	2.25168871	-0.03876594
H	-1.89692110	0.14816645	-0.95065091
H	1.86682440	0.28722364	1.15344456
H	-1.23677618	-2.44914109	-0.30878738
H	1.16503285	-2.36846354	0.93038521
C	1.21549307	2.91869543	-0.56395230
H	2.04782063	2.87150991	0.16777810
H	1.58678874	2.41945990	-1.48069124
H	1.03874316	3.98108728	-0.79236477
C	-1.24620566	2.98614259	0.38548791
H	-1.61208067	2.62681224	1.36761838

H	-1.07426302	4.07144065	0.45203977
H	-2.08073119	2.82589844	-0.32758356

$(S_0/S_1)_{CI}^{60^\circ}$

C	0.90540284	-0.06130099	0.76891652
C	-0.01955946	0.73794819	0.07277524
C	-0.94833405	-0.15112374	-0.49945968
C	0.53749903	-1.50798306	0.62427433
C	-0.59446596	-1.56157197	-0.13368390
C	-0.01731237	2.22769640	-0.03732489
H	-1.79665416	0.13458195	-1.12663212
H	1.75910255	0.30875649	1.34238437
H	-1.15412851	-2.44613502	-0.43880038
H	1.09420557	-2.34078252	1.05485764
C	1.17004548	2.89944061	-0.65567569
H	2.03860931	2.89621336	0.03414136
H	1.49848622	2.36813855	-1.56801867
H	0.95860848	3.94866991	-0.91370632
C	-1.20277024	2.98608242	0.47545515
H	-1.54394790	2.58311899	1.44668609
H	-0.98285157	4.05845635	0.59416110
H	-2.06575189	2.89999390	-0.21632416

$(S_0/S_1)_{CI}^{70^\circ}$

C	0.84688757	-0.05925904	0.86543601
C	-0.02110354	0.73666442	0.07419758
C	-0.89378991	-0.16282068	-0.59099044
C	0.50270082	-1.48150091	0.68493904
C	-0.55511054	-1.54488442	-0.20445576
C	-0.01751545	2.20432551	-0.03532097
H	-1.68100799	0.12288300	-1.29237376
H	1.63657977	0.32268839	1.51642236
H	-1.05630529	-2.44203812	-0.56974356
H	1.00098556	-2.31764372	1.17698924
C	1.11870156	2.88309360	-0.74302121
H	2.01723208	2.94670790	-0.09552580
H	1.41863337	2.31715883	-1.64138319
H	0.85384928	3.91032248	-1.03837347
C	-1.15082166	2.98505314	0.56385070
H	-1.45906371	2.55371560	1.53134484
H	-0.87848039	4.04159879	0.71290771
H	-2.04618817	2.96413505	-0.09087333

$(S_0/S_1)_{CI}^{80^\circ}$

C	0.77693652	-0.06042862	0.95688079
C	-0.02181462	0.73095669	0.07579271
C	-0.82562142	-0.17755378	-0.67860188
C	0.46331313	-1.46126503	0.74371530
C	-0.51199061	-1.53396424	-0.26773156
C	-0.01832748	2.19320679	-0.03407328
H	-1.54548370	0.10853540	-1.44799626
H	1.49771675	0.33231207	1.67677698
H	-0.95218909	-2.44198122	-0.68210691
H	0.90178931	-2.30073343	1.28503299
C	1.06427749	2.87114759	-0.82563110
H	1.99497363	2.98204148	-0.23218979
H	1.32560196	2.28303523	-1.72049841
H	0.75321301	3.88031646	-1.13859765
C	-1.09707584	2.98777718	0.64661119
H	-1.37005144	2.53453726	1.61344258
H	-0.77591009	4.02765357	0.81635665
H	-2.02317416	3.02460644	0.03684364

$(S_0/S_1)_{CI}^{90^\circ}$

C	0.69375721	-0.05648524	1.03400516

C	-0.02075670	0.72899330	0.07384901
C	-0.73821635	-0.18680540	-0.76030066
C	0.41641592	-1.45225344	0.79098734
C	-0.46836134	-1.53239593	-0.31218293
C	-0.01816132	2.19357290	-0.03455130
H	-1.38379911	0.09596461	-1.59397439
H	1.34085821	0.34371823	1.81684704
H	-0.87924047	-2.44487837	-0.74652815
H	0.82312539	-2.29220376	1.35595159
C	1.00739405	2.86679128	-0.90188572
H	1.96433587	3.00052071	-0.35749417
H	1.23093654	2.26009103	-1.79493734
H	0.66919708	3.86480557	-1.22349419
C	-1.04049521	2.99186983	0.72337962
H	-1.27632633	2.51779405	1.69033593
H	-0.69194653	4.02110953	0.90549146
H	-1.99253357	3.05999095	0.15852767

$(S_0/S_1)_{CI}^{100^\circ}$

C	0.59857016	-0.05011722	1.09426609
C	-0.01966820	0.72981459	0.07336679
C	-0.64040956	-0.18962980	-0.82169929
C	0.35615251	-1.46297427	0.82515973
C	-0.41065091	-1.54804940	-0.34223327
C	-0.01755539	2.19371312	-0.03509756
H	-1.20545115	0.08506558	-1.71482889
H	1.16648998	0.35114680	1.93620206
H	-0.78298353	-2.45861135	-0.81362212
H	0.72283659	-2.29549216	1.42718293
C	0.94108190	2.86715910	-0.97477369
H	1.92182037	3.03174237	-0.48377939
H	1.12969567	2.24372133	-1.86401185
H	0.56695036	3.85122281	-1.30007575
C	-0.97429762	3.00058642	0.79512191
H	-1.17578436	2.50776233	1.76015434
H	-0.59090110	4.01620153	0.98463428
H	-1.94971237	3.10693808	0.27805965

$(S_0/S_1)_{CI}^{110^\circ}$

C	0.49581643	-0.05022822	1.14061997
C	-0.02175303	0.72287048	0.07528449
C	-0.54258077	-0.19698066	-0.86418347
C	0.28947521	-1.49185689	0.85253574
C	-0.33731825	-1.58133053	-0.36826866
C	-0.01898931	2.19657158	-0.03442304
H	-1.03140863	0.06847986	-1.80490729
H	0.98450500	0.34955285	2.03266691
H	-0.64088026	-2.48802847	-0.89276589
H	0.59153580	-2.31237827	1.50434990
C	0.86456839	2.87027847	-1.04387065
H	1.85644979	3.12255386	-0.61395382
H	1.04595366	2.21928962	-1.91336339
H	0.41644312	3.81469477	-1.39571653
C	-0.89866661	3.01828013	0.86253067
H	-1.10060776	2.49641705	1.81082001
H	-0.43537868	3.99398825	1.08674558
H	-1.88098074	3.22802598	0.38992544

$(S_0/S_1)_{CI}^{120^\circ}$

C	0.38533632	-0.05396907	1.17702693
C	-0.02402567	0.71247788	0.07668004
C	-0.43834388	-0.20648353	-0.89814713
C	0.21879471	-1.52367225	0.87532578
C	-0.26143894	-1.61638016	-0.38927363
C	-0.01958763	2.21035800	-0.03478954
H	-0.84073184	0.05010585	-1.88201855

H	0.78299972	0.34385502	2.11475962
H	-0.48881147	-2.51833005	-0.95801228
H	0.44780891	-2.33306673	1.56893771
C	0.79092181	2.87829662	-1.10700708
H	1.79747605	3.18801628	-0.75240410
H	0.94327126	2.21307999	-1.97050224
H	0.28439648	3.79316550	-1.45980949
C	-0.82399540	3.03846153	0.92398438
H	-0.99362321	2.50613383	1.87220576
H	-0.30221145	3.98521984	1.14707086
H	-1.82205240	3.31293130	0.51999893

$(S_0/S_1)_{CI}^{130^\circ}$

C	0.37364300	-0.02960034	1.17478165
C	-0.02412505	0.73360250	0.07560647
C	-0.42799648	-0.18379899	-0.89759287
C	0.21032413	-1.51241559	0.87508148
C	-0.25238624	-1.60588525	-0.38781690
C	-0.01894296	2.25019235	-0.03892214
H	-0.81924518	0.06862923	-1.88712081
H	0.76005440	0.36572718	2.11832607
H	-0.47164207	-2.50469305	-0.96430712
H	0.43308198	-2.31664467	1.57642555
C	0.62081150	2.86327932	-1.24281801
H	0.83406427	3.93385954	-1.09550967
H	1.56646447	2.34819338	-1.49929404
H	-0.02955398	2.77842919	-2.13836129
C	-0.65354947	3.04293879	1.05818411
H	-1.61987667	2.59974827	1.36726318
H	-0.01814359	3.05911488	1.96802915
H	-0.82679870	4.08952310	0.76207117

$(S_0/S_1)_{CI}^{140^\circ}$

C	0.26649337	-0.03162134	1.20265951
C	-0.02363912	0.73027001	0.07872017
C	-0.32022816	-0.18337020	-0.92424818
C	0.14514019	-1.53200367	0.88945679
C	-0.18650073	-1.62437652	-0.40359588
C	-0.01914747	2.27519025	-0.03815012
H	-0.61398192	0.06497875	-1.94828836
H	0.55222346	0.36014490	2.18327369
H	-0.34684328	-2.51738153	-1.00746096
H	0.30597691	-2.32980314	1.61438357
C	0.53488207	2.86565940	-1.29122065
H	0.70421934	3.94951712	-1.19401040
H	1.49379655	2.38609032	-1.57457977
H	-0.14672671	2.71575344	-2.15503057
C	-0.56717243	3.05843725	1.10660222
H	-1.53485418	2.64406742	1.45544472
H	0.11009422	3.03144099	1.98633988
H	-0.71754876	4.11720638	0.84373032

$(S_0/S_1)_{CI}^{150^\circ}$

C	0.15709221	-0.02746097	1.21972808
C	-0.02452426	0.72760831	0.07556386
C	-0.21258934	-0.18865889	-0.94378516
C	0.07854880	-1.54030134	0.90411486
C	-0.12443724	-1.63900388	-0.40802801
C	-0.01878762	2.29761067	-0.04358064
H	-0.38724469	0.05316341	-1.99629185
H	0.33844982	0.36756013	2.22353857
H	-0.21845064	-2.53130308	-1.02668101
H	0.17160178	-2.33098096	1.64828670
C	0.44455354	2.87374838	-1.33485339
H	0.59370532	3.96252997	-1.26417838
H	1.40054823	2.41503198	-1.66377561

H	-0.27742584	2.69272714	-2.15994450
C	-0.47748959	3.06112491	1.14672030
H	-1.44382196	2.67294568	1.53173303
H	0.23733476	2.98211788	1.99433086
H	-0.60087994	4.13174050	0.92112827

$(S_0/S_1)_{CI}^{160^\circ}$

C	0.04953331	-0.02663955	1.22808983
C	-0.02237451	0.72458985	0.07419464
C	-0.09920251	-0.19035060	-0.95487987
C	0.01576113	-1.54838625	0.90856248
C	-0.05997913	-1.64680019	-0.41296500
C	-0.01761092	2.31287895	-0.04371166
H	-0.17292026	0.04952639	-2.01981893
H	0.11182493	0.36967216	2.24597956
H	-0.09684198	-2.53747464	-1.03976696
H	0.04972547	-2.33547556	1.66128342
C	0.35363286	2.87419630	-1.36717342
H	0.43821215	3.97131745	-1.33072796
H	1.32018524	2.46447391	-1.73260482
H	-0.39366619	2.62504381	-2.15171085
C	-0.38468656	3.06739922	1.18130028
H	-1.35987145	2.73270971	1.59707815
H	0.35535915	2.92525539	1.99846978
H	-0.45089738	4.14826350	0.98242729

$(S_0/S_1)_{CI}^{170^\circ}$

C	-0.06195094	-0.02642983	1.22862329
C	-0.02216778	0.72439975	0.07495180
C	0.01327007	-0.18962340	-0.95451028
C	-0.05101276	-1.55224407	0.90947224
C	0.00342657	-1.65120208	-0.41188357
C	-0.01797492	2.32334202	-0.04491489
H	0.04936303	0.05076562	-2.02134013
H	-0.10404776	0.36979418	2.24764205
H	0.03495467	-2.54020555	-1.04129000
H	-0.08181794	-2.33782621	1.66409461
C	0.25993766	2.87433668	-1.39294436
H	0.30665894	3.97404625	-1.37482133
H	1.22188226	2.49655962	-1.80378339
H	-0.51701772	2.58388667	-2.13432411
C	-0.29242630	3.07072128	1.20551322
H	-1.26254156	2.77445230	1.66184873
H	0.47671809	2.87894427	1.98598782
H	-0.31907026	4.15648238	1.02570427

$(S_0/S_1)_{CI}^{180^\circ}$

C	-0.17057840	-0.02774161	1.21848879
C	-0.02534994	0.72169530	0.07344590
C	0.11118049	-0.19165346	-0.94670754
C	-0.12268190	-1.55496450	0.90255897
C	0.07818876	-1.65389032	-0.40381652
C	-0.01718730	2.32508356	-0.04523651
H	0.22482090	0.04877187	-2.00816654
H	-0.30205977	0.36887132	2.22979636
H	0.17622987	-2.54295092	-1.02604581
H	-0.20856826	-2.34037425	1.65293648
C	0.18595610	2.87566011	-1.40618289
H	0.23801631	3.97513895	-1.38884482
H	1.11833653	2.49580706	-1.87700374
H	-0.63733888	2.58911571	-2.09889773
C	-0.21373575	3.07281819	1.21970518
H	-1.14953135	2.77583448	1.74071057
H	0.60712549	2.88495292	1.94774829
H	-0.25663954	4.15802547	1.03953752

6.3.4 RMS-CASPT2

$(S_0)_{min}$

C	1.15548219	-0.15755320	0.04811875
C	-0.02205370	0.73347061	0.04798574
C	-1.20826798	-0.14592786	0.05734534
C	0.70487955	-1.45977873	0.05676044
C	-0.77044372	-1.45252200	0.06253020
C	-0.01534780	2.10321087	0.04040254
H	-2.24544270	0.18246496	0.05969716
H	2.19582674	0.16061328	0.04241885
H	-1.40138796	-2.34355272	0.06969099
H	1.32706566	-2.35697253	0.05905857
C	1.25588050	2.91026998	0.03109575
H	1.28462038	3.57060272	0.91487537
H	2.15337692	2.28397183	0.02903361
H	1.27589562	3.56395973	-0.85782096
C	-1.27860790	2.92273571	0.04078724
H	-1.29283657	3.58531428	0.92322229
H	-1.30025265	3.57458151	-0.84945971
H	-2.18220321	2.30531141	0.04828374

$(S_1)_{min}$

C	1.10226093	-0.15759602	0.04844058
C	-0.02221335	0.70182941	0.04761105
C	-1.15504938	-0.14649553	0.05667198
C	0.65760524	-1.56193912	0.05805697
C	-0.72416829	-1.55514427	0.06308290
C	-0.01496530	2.18220224	0.03879856
H	-2.19807003	0.17258742	0.05866447
H	2.14835659	0.15121459	0.04297780
H	-1.39293574	-2.41631868	0.07051190
H	1.31790728	-2.42964929	0.06064884
C	1.26726587	2.95402674	0.03048671
H	1.33516803	3.61716234	0.91507053
H	2.15151589	2.30502827	0.02719947
H	1.32568395	3.61342227	-0.85747230
C	-1.28956579	2.96660639	0.04054741
H	-1.34226210	3.63294302	0.92372599
H	-1.35020609	3.62396726	-0.84880517
H	-2.18014432	2.32635281	0.04780824

$(S_0/S_1)_{MECI}$

C	0.81190453	-0.01820314	0.76033294
C	-0.08534888	0.73097234	-0.05139900
C	-0.91254509	-0.20813742	-0.70410627
C	0.52781147	-1.45445474	0.60304753
C	-0.52078216	-1.57420463	-0.28918373
C	-0.13295325	2.19989711	-0.18891576
H	-1.70257368	0.03453500	-1.41845153
H	1.58738779	0.40457849	1.40319514
H	-0.98790412	-2.49577704	-0.63856328
H	1.05317731	-2.26095409	1.11566448
C	1.11076994	2.91317996	-0.63130927
H	1.81235354	3.04691975	0.21983480
H	1.64387632	2.33857119	-1.40479820
H	0.88017498	3.91929502	-1.01728971
C	-1.16379302	2.95433494	0.60049386
H	-0.84870331	3.05119656	1.66178166
H	-1.30683797	3.97282054	0.20503495
H	-2.12983101	2.42562996	0.59865733

$(S_0/S_1)_{CI}^{0^\circ}$

C	1.05981680	-0.17881248	0.05354129
C	-0.02237707	0.66233556	0.04977432
C	-1.11275936	-0.16806740	0.05927552

C	0.62590586	-1.68680726	0.06665373
C	-0.69356712	-1.68019475	0.06928486
C	-0.01442525	2.29658570	0.03680242
H	-2.16104593	0.14505587	0.06095909
H	2.11108808	0.12421508	0.04933502
H	-1.39737390	-2.51185215	0.08224796
H	1.32149100	-2.52544023	0.06660255
C	1.28982179	3.02225058	0.02755839
H	1.43486347	3.64429288	0.93612053
H	2.14925352	2.33932173	-0.02967184
H	1.37179827	3.71464808	-0.83583193
C	-1.31150283	3.03510381	0.03442435
H	-1.43337600	3.67756212	0.93181721
H	-1.40298578	3.71007005	-0.84204793
H	-2.17844218	2.35993264	0.00718043

$(S_0/S_1)_{CI}^{10^\circ}$

C	1.06655055	-0.10474891	0.23610417
C	-0.02164927	0.72298889	0.07273758
C	-1.11401247	-0.11404079	0.02925719
C	0.63227821	-1.60005950	0.30281446
C	-0.68809539	-1.60528656	0.18428647
C	-0.01706106	2.32259681	-0.04240026
H	-2.15526875	0.19996076	-0.09029893
H	2.10962291	0.21787415	0.30759132
H	-1.38199915	-2.44543759	0.18832669
H	1.32154683	-2.43531360	0.42362309
C	1.31238757	2.96151772	-0.19495956
H	1.96571118	2.78888044	0.68869779
H	1.87115155	2.55244476	-1.06509124
H	1.22047394	4.04994864	-0.33215739
C	-1.34229019	2.98533207	0.01642841
H	-1.89974386	2.71701467	0.94068272
H	-1.24311845	4.08134828	-0.01308278
H	-2.00030079	2.68517958	-0.82853377

$(S_0/S_1)_{CI}^{20^\circ}$

C	1.05213511	-0.09649071	0.34718011
C	-0.02118098	0.72414531	0.07142078
C	-1.09853406	-0.12242322	-0.08450557
C	0.62361701	-1.59130268	0.36896495
C	-0.68112019	-1.60603660	0.12268883
C	-0.01650251	2.31250376	-0.04257724
H	-2.12414669	0.18367979	-0.31140702
H	2.08022702	0.23350784	0.52379670
H	-1.36593150	-2.45177991	0.06549842
H	1.30265950	-2.42279738	0.55593575
C	1.30003591	2.95359705	-0.28864895
H	2.00413830	2.80397843	0.55865615
H	1.80798200	2.52718998	-1.18076569
H	1.19545375	4.03895636	-0.44057069
C	-1.32941647	2.98905916	0.10896460
H	-1.84294257	2.68788366	1.04764716
H	-1.21835903	4.08437301	0.11548564
H	-2.03193123	2.73215599	-0.71373795

$(S_0/S_1)_{CI}^{30^\circ}$

C	1.02871930	-0.08917921	0.45313998
C	-0.02068706	0.72597912	0.07035765
C	-1.07383648	-0.12936874	-0.19514934
C	0.61000178	-1.57972912	0.42914356
C	-0.66821131	-1.60264673	0.05584371
C	-0.01689047	2.29637455	-0.03980357
H	-2.07171556	0.17042438	-0.52875580
H	2.02982720	0.24893077	0.73678736
H	-1.33581962	-2.45557003	-0.06397123

H	1.27042244	-2.41049697	0.67672008
C	1.27876446	2.94370745	-0.38072668
H	2.01678028	2.86401049	0.44628876
H	1.75737629	2.46199361	-1.25935061
H	1.15106039	4.01475117	-0.60223671
C	-1.30873530	2.99244821	0.20675291
H	-1.77595148	2.66672540	1.16045417
H	-1.17771546	4.08503752	0.24378298
H	-2.05720602	2.77680798	-0.58525122

$(S_0/S_1)_{CI}^{40^\circ}$

C	0.99584147	-0.07711825	0.56177710
C	-0.01986651	0.73035009	0.06969822
C	-1.03967522	-0.13679753	-0.30098309
C	0.59110630	-1.55974110	0.49838244
C	-0.64983066	-1.59324233	-0.00125572
C	-0.01532316	2.27569094	-0.04200503
H	-1.99569932	0.15433421	-0.74595140
H	1.95678968	0.27144872	0.95123239
H	-1.29663383	-2.45350275	-0.17336106
H	1.22675147	-2.38759589	0.81221445
C	1.25141834	2.93179978	-0.47664798
H	2.04560082	2.84938442	0.29435840
H	1.66472266	2.45563476	-1.38978969
H	1.10398467	4.00330728	-0.68257367
C	-1.28070144	2.98980014	0.29440919
H	-1.71630422	2.61355911	1.24259233
H	-1.12404469	4.07539402	0.39189858
H	-2.06195299	2.83749423	-0.47996947

$(S_0/S_1)_{CI}^{50^\circ}$

C	0.95354281	-0.07076747	0.66630021
C	-0.01943865	0.73512416	0.07013684
C	-0.99704281	-0.14075049	-0.40691896
C	0.56421148	-1.53729970	0.55717048
C	-0.62668797	-1.57822638	-0.07324214
C	-0.01563218	2.25301753	-0.03756697
H	-1.90288418	0.14851061	-0.94686421
H	1.86539235	0.28605167	1.15312705
H	-1.23633779	-2.44929095	-0.31371014
H	1.16407344	-2.36913494	0.92663468
C	1.21672966	2.91695331	-0.56145083
H	2.04440720	2.87851564	0.17655011
H	1.59398829	2.40806993	-1.47014990
H	1.04073428	3.97704491	-0.80135096
C	-1.24562225	2.98825310	0.38747760
H	-1.60794486	2.63610068	1.37362501
H	-1.07283325	4.07394602	0.44613935
H	-2.08247222	2.82408221	-0.32188122

$(S_0/S_1)_{CI}^{60^\circ}$

C	0.90425512	-0.06253610	0.76760320
C	-0.02008089	0.73796027	0.07157286
C	-0.94859156	-0.15041713	-0.50225728
C	0.53630957	-1.50917206	0.62127294
C	-0.59437162	-1.56162581	-0.13862272
C	-0.01671596	2.22742946	-0.03641748
H	-1.79862387	0.13641560	-1.12671690
H	1.75745443	0.30704450	1.34226250
H	-1.15652629	-2.44557656	-0.44098204
H	1.09116532	-2.34270959	1.05277320
C	1.17170747	2.89928465	-0.65298238
H	2.03868610	2.89829953	0.03898692
H	1.50305240	2.36734778	-1.56387165
H	0.95967777	3.94796754	-0.91289567
C	-1.20195275	2.98659594	0.47641681

H	-1.53754168	2.59064600	1.45261263
H	-0.98379150	4.06025862	0.58573293
H	-2.06792870	2.89298722	-0.21046186

$(S_0/S_1)_{CI}^{70^\circ}$

C	0.84782761	-0.06078626	0.86401217
C	-0.02016823	0.73601416	0.07389695
C	-0.89381427	-0.16262009	-0.59176935
C	0.50355711	-1.48313718	0.68309900
C	-0.55827102	-1.54495383	-0.20317477
C	-0.01681367	2.20390610	-0.03514122
H	-1.68195617	0.12457291	-1.29237451
H	1.63878565	0.32082022	1.51451344
H	-1.06218851	-2.44116791	-0.56700069
H	1.00154556	-2.31940938	1.17542882
C	1.11777634	2.88439281	-0.74265335
H	2.01383380	2.95108630	-0.09213298
H	1.42044380	2.31601930	-1.63887043
H	0.85268789	3.91096112	-1.04069529
C	-1.14902464	2.98498652	0.56435982
H	-1.46780401	2.54425831	1.52428317
H	-0.87227152	4.03842744	0.72763164
H	-2.03796234	2.97682932	-0.09938641

$(S_0/S_1)_{CI}^{80^\circ}$

C	0.77848283	-0.06177501	0.95373562
C	-0.02276015	0.72945008	0.07494549
C	-0.82871688	-0.17869268	-0.67749323
C	0.46430673	-1.46303185	0.74125145
C	-0.51455866	-1.53524016	-0.26720545
C	-0.01840278	2.19125101	-0.03343353
H	-1.54967317	0.10743550	-1.44598523
H	1.50004612	0.33111130	1.67290336
H	-0.95590148	-2.44299004	-0.68101692
H	0.90394965	-2.30238023	1.28182917
C	1.06172935	2.87098108	-0.82675485
H	1.98273663	3.01140592	-0.22442381
H	1.34332654	2.26932847	-1.70603025
H	0.73741317	3.86841567	-1.16379742
C	-1.09315498	2.98885464	0.64968441
H	-1.39125793	2.51674453	1.59962048
H	-0.75439446	4.01766115	0.85139003
H	-2.00698713	3.06167046	0.02480669

$(S_0/S_1)_{CI}^{90^\circ}$

C	0.69489973	-0.05740857	1.03312652
C	-0.02078408	0.72821139	0.07386986
C	-0.73921045	-0.18779758	-0.75927554
C	0.41735939	-1.45326395	0.79060271
C	-0.46929661	-1.53346899	-0.31120555
C	-0.01827085	2.19274449	-0.03447785
H	-1.38581421	0.09492460	-1.59217246
H	1.34215174	0.34310856	1.81569001
H	-0.88040468	-2.44605525	-0.74515932
H	0.82433004	-2.29326219	1.35529219
C	1.00581243	2.86698861	-0.90279923
H	1.96200748	3.00457372	-0.35807181
H	1.23138624	2.25943693	-1.79472749
H	0.66484467	3.86353357	-1.22621113
C	-1.03890995	2.99271392	0.72404807
H	-1.29442289	2.50549048	1.67920499
H	-0.67770154	4.01351070	0.92879180
H	-1.98179305	3.08621941	0.14750026

$(S_0/S_1)_{CI}^{100^\circ}$

C	0.59791140	-0.04891375	1.09480119
C	-0.01928540	0.73145533	0.07345734
C	-0.63920308	-0.18790797	-0.82268912
C	0.35606721	-1.46226554	0.82551019
C	-0.41086484	-1.54615405	-0.34144999
C	-0.01749494	2.19437061	-0.03515611
H	-1.20610218	0.08651666	-1.71483781
H	1.16822205	0.35226998	1.93523743
H	-0.78488947	-2.45621596	-0.81246934
H	0.72420158	-2.29476494	1.42672249
C	0.94255990	2.86619784	-0.97444260
H	1.92900411	3.00948340	-0.48827315
H	1.11689835	2.24962111	-1.87194016
H	0.58085965	3.85910182	-1.28616646
C	-0.97619089	3.00024577	0.79483603
H	-1.18018202	2.50500850	1.75824679
H	-0.59504789	4.01618205	0.98751379
H	-1.95028012	3.10596898	0.27512553

$(S_0/S_1)_{CI}^{110^\circ}$

C	0.49424589	-0.04895237	1.14156016
C	-0.02193925	0.72405297	0.07526618
C	-0.54073847	-0.19610861	-0.86476700
C	0.28909617	-1.49049223	0.85344808
C	-0.33603838	-1.58037427	-0.36838537
C	-0.01955204	2.19779718	-0.03441416
H	-1.02778344	0.06890282	-1.80652190
H	0.98105380	0.35108683	2.03450944
H	-0.63871080	-2.48710754	-0.89333320
H	0.59107067	-2.31070796	1.50567466
C	0.86634350	2.86926411	-1.04324440
H	1.87040091	3.08746699	-0.62314009
H	1.02032223	2.23034062	-1.92718408
H	0.43706231	3.83040189	-1.37150253
C	-0.90134835	3.01824066	0.86156037
H	-1.09869605	2.49837635	1.81200094
H	-0.44263741	3.99688461	1.08168680
H	-1.88596786	3.22112780	0.39081214

$(S_0/S_1)_{CI}^{120^\circ}$

C	0.38491635	-0.05259146	1.17895438
C	-0.02398705	0.71329233	0.07740072
C	-0.43606241	-0.20637245	-0.89728382
C	0.22036180	-1.52226226	0.87798814
C	-0.25778021	-1.61613218	-0.38771784
C	-0.02122986	2.21142381	-0.03551971
H	-0.83732593	0.04908756	-1.88185199
H	0.78187547	0.34601988	2.11663462
H	-0.48345146	-2.51830057	-0.95677592
H	0.45153119	-2.33088718	1.57175785
C	0.79036566	2.87712516	-1.10795880
H	1.81370396	3.14425728	-0.76701302
H	0.90520568	2.22672359	-1.98879478
H	0.30777452	3.81527720	-1.43088665
C	-0.82814841	3.03860483	0.92196065
H	-1.01229425	2.49966885	1.86369934
H	-0.30005222	3.97861751	1.15898424
H	-1.81921940	3.32664793	0.51044863

$(S_0/S_1)_{CI}^{130^\circ}$

C	0.35238399	0.02519956	1.23546447
C	-0.00417484	0.73532306	0.08690493
C	-0.27680452	-0.22896758	-0.88599015
C	0.30068822	-1.47154460	0.96844190
C	-0.05682669	-1.62462273	-0.32246666
C	-0.08016127	2.24606752	-0.07246256

H	-0.60525970	-0.02609672	-1.90914289
H	0.64038091	0.46700575	2.19348177
H	-0.17745014	-2.54920648	-0.88715779
H	0.51670333	-2.24281908	1.70787086
C	0.61333531	2.86533591	-1.24308374
H	1.61877138	2.42634140	-1.39247407
H	0.05697982	2.68694563	-2.18673186
H	0.72049785	3.95538201	-1.12823818
C	-0.84321550	3.02613782	0.94928432
H	-1.79663312	2.52555137	1.20631798
H	-0.27689350	3.11607150	1.89961904
H	-1.06613810	4.04809548	0.60438868

$(S_0/S_1)_{CI}^{140^\circ}$

C	0.24602760	0.02445425	1.25304568
C	-0.00374444	0.73200263	0.08580909
C	-0.16705217	-0.22809989	-0.90497496
C	0.24069467	-1.48979243	0.98345131
C	0.01704306	-1.64055514	-0.32731169
C	-0.08341415	2.27084821	-0.07642037
H	-0.39463466	-0.02903699	-1.95636446
H	0.42918640	0.46241683	2.23855325
H	-0.03653401	-2.55907118	-0.91150282
H	0.39115935	-2.25459163	1.74527700
C	0.52673366	2.86414631	-1.30099256
H	1.52890153	2.43389188	-1.50165348
H	-0.07987738	2.65659717	-2.20778691
H	0.62800214	3.95778101	-1.22020854
C	-0.76360412	3.04251361	1.00311976
H	-1.74492552	2.59671615	1.26599470
H	-0.17118385	3.04755644	1.94187474
H	-0.92659468	4.09242260	0.71411629

$(S_0/S_1)_{CI}^{150^\circ}$

C	0.13079562	0.01837734	1.26121061
C	-0.00368860	0.72831156	0.08310000
C	-0.05457427	-0.22510064	-0.91734399
C	0.16858963	-1.50683829	0.99054871
C	0.08043177	-1.65252401	-0.33050387
C	-0.08180879	2.29036032	-0.07941407
H	-0.16455773	-0.02361935	-1.98714134
H	0.20023498	0.45193981	2.26329845
H	0.08422542	-2.56508209	-0.92633722
H	0.25325832	-2.26895903	1.76497876
C	0.44750932	2.86449003	-1.34590147
H	1.44743598	2.45138255	-1.59357213
H	-0.20138013	2.62661028	-2.21660391
H	0.53170298	3.96104247	-1.29162776
C	-0.67845205	3.05560996	1.04846293
H	-1.62937052	2.59765341	1.39150048
H	-0.01384829	3.07480738	1.93944208
H	-0.88032023	4.10173815	0.76992977

$(S_0/S_1)_{CI}^{160^\circ}$

C	0.02838220	0.01076492	1.26464154
C	-0.00070426	0.72659483	0.08651605
C	0.05769873	-0.21831061	-0.91695336
C	0.11229857	-1.51776422	0.99182056
C	0.13852093	-1.65474211	-0.32847870
C	-0.08402827	2.30844737	-0.08131898
H	0.06754407	-0.00955022	-1.99104998
H	-0.00719312	0.43696334	2.27176070
H	0.20442587	-2.56163870	-0.92898182
H	0.12512951	-2.28247778	1.76817933
C	0.35220920	2.85769313	-1.38972229
H	1.36763513	2.50165473	-1.66815993

H	-0.31558514	2.54288267	-2.22106856
H	0.36737170	3.95849937	-1.37907955
C	-0.58963042	3.06832289	1.08927941
H	-1.57125398	2.68030313	1.43870312
H	0.09162871	2.99540934	1.96454923
H	-0.70826602	4.13714776	0.85338926

$(S_0/S_1)_{CI}^{170^\circ}$

C	-0.08316449	0.00392763	1.25622898
C	-0.00001087	0.72498773	0.08620401
C	0.17080528	-0.21167117	-0.90846198
C	0.04561203	-1.52561563	0.98613540
C	0.20522675	-1.65587969	-0.32336899
C	-0.08426782	2.31739877	-0.08181475
H	0.27907973	0.00254612	-1.97592536
H	-0.22836786	0.42340639	2.25625431
H	0.33559098	-2.55818609	-0.92025444
H	-0.00160896	-2.29225898	1.75904148
C	0.26281996	2.85132903	-1.42078981
H	1.27617397	2.52833739	-1.74553633
H	-0.43354472	2.49240092	-2.21048639
H	0.23794544	3.95188676	-1.43171632
C	-0.49989505	3.07919851	1.12037784
H	-1.48119979	2.73218347	1.51221405
H	0.21754538	2.96080013	1.96203933
H	-0.58255656	4.15540853	0.90388500

$(S_0/S_1)_{CI}^{180^\circ}$

C	-0.19409677	-0.00397333	1.23792697
C	0.00020941	0.72457403	0.08662623
C	0.28225033	-0.20337738	-0.88975155
C	-0.02121889	-1.53170371	0.97501292
C	0.26614701	-1.65233774	-0.31282759
C	-0.08429637	2.32078690	-0.08209661
H	0.49430283	0.01856413	-1.93998060
H	-0.43788381	0.40848982	2.22161554
H	0.46186202	-2.54936199	-0.89964712
H	-0.12881237	-2.30233639	1.73782521
C	0.17140715	2.84409490	-1.44481281
H	1.18157274	2.56420566	-1.81720334
H	-0.54620111	2.43664555	-2.19085439
H	0.09606354	3.94202886	-1.47181855
C	-0.40828242	3.08714431	1.14432618
H	-1.38864057	2.78831449	1.57694058
H	0.33861001	2.92003308	1.95156536
H	-0.44680933	4.16840863	0.94117960

6.3.5 XDW-CASPT2

$(S_0)_{min}$

C	1.15548386	-0.15754388	0.04811921
C	-0.02205391	0.73347489	0.04798713
C	-1.20826979	-0.14591886	0.05734513
C	0.70487918	-1.45976645	0.05675942
C	-0.77044300	-1.45250991	0.06253124
C	-0.01534797	2.10321249	0.04040218
H	-2.24544248	0.18247660	0.05969910
H	2.19582627	0.16062570	0.04242075
H	-1.40139119	-2.34353274	0.06968907
H	1.32706956	-2.35695222	0.05905781
C	1.25588569	2.91026030	0.03109553
H	1.28463097	3.57058915	0.91487791
H	2.15337361	2.28394848	0.02903197
H	1.27590488	3.56394868	-0.85782206
C	-1.27861322	2.92272650	0.04078667

H	-1.29284613	3.58530287	0.92322334
H	-1.30026257	3.57456940	-0.84946231
H	-2.18220041	2.30528882	0.04828384

$(S_1)_{min}$

C	1.10226203	-0.15759089	0.04836703
C	-0.02221292	0.70183612	0.04770650
C	-1.15505036	-0.14648953	0.05675017
C	0.65760338	-1.56192909	0.05794864
C	-0.72416668	-1.55513364	0.06307893
C	-0.01496447	2.18220018	0.03901675
H	-2.19807024	0.17259405	0.05882328
H	2.14835687	0.15121957	0.04281873
H	-1.39294303	-2.41629647	0.07045585
H	1.31791381	-2.42962820	0.06040631
C	1.26727037	2.95401906	0.03058762
H	1.33503994	3.61748586	0.91492763
H	2.15151812	2.30501353	0.02766721
H	1.32582059	3.61307643	-0.85761884
C	-1.28956995	2.96659826	0.04054959
H	-1.34228969	3.63313037	0.92357525
H	-1.35018994	3.62375617	-0.84895792
H	-2.18014448	2.32633804	0.04792322

$(S_0/S_1)_{MECI}$

$(S_0/S_1)_{CI}^{0^\circ}$

C	1.07426174	-0.19447353	0.04818669
C	-0.00120128	0.66265669	0.04674991
C	-1.09732943	-0.15761672	0.05566620
C	0.62467788	-1.69593656	0.05898096
C	-0.69524766	-1.68209889	0.06913381
C	-0.01802556	2.30339197	0.03675346
H	-2.14177533	0.16806008	0.06844854
H	2.12994902	0.09097913	0.04322718
H	-1.41332254	-2.50131622	0.08269329
H	1.31236271	-2.54160901	0.06681446
C	1.28571124	3.03191835	0.02766227
H	1.41595850	3.68282998	0.91765902
H	2.14509384	2.34647692	0.00852724
H	1.38782922	3.69495088	-0.85694800
C	-1.32053272	3.03383785	0.03689819
H	-1.45013450	3.66412021	0.94259831
H	-1.41713301	3.71821439	-0.83088812
H	-2.18495877	2.35581432	0.00186256

$(S_0/S_1)_{CI}^{10^\circ}$

C	1.06846685	-0.19415917	0.14435073
C	0.00045729	0.66348752	0.06113414
C	-1.09673660	-0.15162368	-0.00442934
C	0.62000813	-1.69599553	0.13127752
C	-0.69112455	-1.67533025	-0.00891310
C	-0.01436553	2.29889547	0.04090223
H	-2.13924111	0.17626900	-0.05615789
H	2.12286021	0.09116291	0.20260400
H	-1.40696934	-2.49509910	-0.06392740
H	1.30509966	-2.54236710	0.16999625
C	1.28461704	3.03223720	-0.03932229
H	1.46414341	3.68874640	0.83531451
H	2.14447055	2.35072255	-0.11110572
H	1.32729485	3.69170305	-0.93409770
C	-1.31608533	3.02706948	0.10247337
H	-1.41459627	3.65143784	1.01621301
H	-1.44300615	3.71718997	-0.75682432
H	-2.17910974	2.34585327	0.09453796

$(S_0/S_1)_{CI}^{20^\circ}$

C	1.04247821	-0.12553178	0.33325687
C	-0.03045484	0.70079513	0.05483982
C	-1.08088689	-0.14549017	-0.18778153
C	0.64091496	-1.62824443	0.26184743
C	-0.64893039	-1.64736019	-0.03830458
C	-0.04779687	2.30650568	0.02152171
H	-2.09779205	0.15506548	-0.45863055
H	2.05661075	0.20490072	0.57657877
H	-1.32543020	-2.49241234	-0.16473080
H	1.32659534	-2.45824279	0.43086020
C	1.28184736	2.95150893	-0.13721202
H	1.90245514	2.88611425	0.78462676
H	1.87521352	2.45628167	-0.93481416
H	1.19059893	4.01934432	-0.39422027
C	-1.34039321	3.04508113	0.14589275
H	-1.31445601	3.83086796	0.92689991
H	-1.62504956	3.55386992	-0.79920270
H	-2.16934082	2.36714634	0.40259835

$(S_0/S_1)^{30^\circ}_{CI}$

C	1.00872880	-0.13386608	0.45195084
C	0.00059603	0.72617070	0.05155051
C	-1.03631450	-0.09217448	-0.35552740
C	0.58732367	-1.61262367	0.28972185
C	-0.65575379	-1.59269472	-0.18654917
C	-0.01282397	2.30362719	0.04184537
H	-2.00625776	0.24639727	-0.73044955
H	1.98822674	0.16289457	0.83790419
H	-1.33028836	-2.42039237	-0.40484161
H	1.21791654	-2.46834930	0.53155983
C	1.29712476	2.97832501	-0.15789422
H	1.96312777	2.86186470	0.72513358
H	1.85440897	2.53949208	-1.01207786
H	1.17745431	4.05882301	-0.33428648
C	-1.32926010	2.97030466	0.23260026
H	-1.21148029	4.06138669	0.32385652
H	-2.01833773	2.78698563	-0.61850145
H	-1.85820771	2.60402894	1.13803075

$(S_0/S_1)^{40^\circ}_{CI}$

C	0.95598223	-0.13435725	0.55286742
C	-0.00683150	0.73065950	0.05087172
C	-0.99704996	-0.09544877	-0.46529305
C	0.56192633	-1.59705583	0.34750970
C	-0.63084952	-1.57781331	-0.25150044
C	-0.01427716	2.28527657	0.04689207
H	-1.92145186	0.24168979	-0.94389067
H	1.88717122	0.15996512	1.04345411
H	-1.27258985	-2.40997986	-0.53819341
H	1.17358497	-2.45272595	0.63365436
C	1.28240932	2.96588685	-0.24222213
H	1.98305935	2.92973144	0.61881709
H	1.81364012	2.48687434	-1.08967769
H	1.13535164	4.02971456	-0.48428906
C	-1.30757262	2.96994016	0.33031616
H	-1.18178491	4.06201904	0.38849984
H	-2.06306252	2.75978060	-0.45631728
H	-1.76147191	2.62604283	1.28252722

$(S_0/S_1)^{50^\circ}_{CI}$

C	0.90493312	-0.13190753	0.65231300
C	-0.00880475	0.73465784	0.04982656
C	-0.95209109	-0.09054994	-0.56905414
C	0.53162437	-1.58207978	0.40940451
C	-0.60385225	-1.56000845	-0.31683562
C	-0.00930212	2.25961199	0.04676424

H	-1.81386785	0.24971676	-1.14964436
H	1.77511885	0.17249596	1.24022387
H	-1.20089384	-2.40138054	-0.66958084
H	1.10193610	-2.44434839	0.75523042
C	1.25374113	2.96096371	-0.33289186
H	1.99088969	2.96066746	0.49785433
H	1.75017551	2.45574228	-1.18344036
H	1.07555242	4.01361970	-0.60425012
C	-1.27619935	2.95888556	0.42462485
H	-1.14047722	4.05079873	0.45526241
H	-2.08928913	2.74340125	-0.29664579
H	-1.65301020	2.62991322	1.41486485

$(S_0/S_1)^{60°}_{CI}$

C	0.83950633	-0.12533404	0.74899644
C	-0.01721342	0.73721278	0.04838719
C	-0.90194217	-0.09353349	-0.66807784
C	0.49515658	-1.55618108	0.47467258
C	-0.56593397	-1.53457909	-0.37873342
C	-0.01101669	2.23466997	0.04729728
H	-1.70162490	0.24635711	-1.33082085
H	1.64237246	0.19111638	1.42035451
H	-1.09892385	-2.38856805	-0.79889842
H	1.02180258	-2.42189319	0.87611090
C	1.22174635	2.94511955	-0.42200371
H	2.00241337	2.97179250	0.36625900
H	1.67227328	2.42695678	-1.28796932
H	1.01122400	3.98856067	-0.70549030
C	-1.23906424	2.95236965	0.51601410
H	-1.08648452	4.04192894	0.54432256
H	-2.09942489	2.74875451	-0.15149566
H	-1.54868292	2.61544992	1.52510092

$(S_0/S_1)^{70°}_{CI}$

C	0.76809215	-0.12456699	0.84392337
C	-0.02071132	0.73910824	0.04817949
C	-0.83679489	-0.09774005	-0.75974231
C	0.45026215	-1.53035077	0.53884643
C	-0.52413623	-1.50881963	-0.44166253
C	-0.00982238	2.21480730	0.04597463
H	-1.56003139	0.24589117	-1.50255085
H	1.49997635	0.19970364	1.58796070
H	-0.99090122	-2.37242651	-0.91823531
H	0.91398615	-2.40283597	0.99954943
C	1.18386443	2.93470226	-0.50987915
H	2.01457983	2.97543855	0.22442468
H	1.57732549	2.41528361	-1.40044862
H	0.93833208	3.97328438	-0.78172816
C	-1.19766829	2.94293853	0.60192620
H	-1.03982457	4.03181460	0.60955518
H	-2.10656765	2.73243797	0.00492103
H	-1.42377730	2.61152949	1.63301176

$(S_0/S_1)^{80°}_{CI}$

C	0.69277053	-0.13062730	0.93313475
C	-0.01687956	0.73687349	0.04823125
C	-0.75357755	-0.10068576	-0.84323665
C	0.39684094	-1.50820041	0.59130375
C	-0.48086985	-1.48961261	-0.50670974
C	-0.00231982	2.20461961	0.04317136
H	-1.39411629	0.25216470	-1.65427215
H	1.35083869	0.19499192	1.74150642
H	-0.89978067	-2.35809880	-1.01706661
H	0.77721218	-2.38960363	1.10963829
C	1.14430749	2.93680270	-0.59504791
H	2.01391210	3.01831725	0.08879314

H	1.49624660	2.40608934	-1.49488546
H	0.85260827	3.96130131	-0.87483427
C	-1.15023056	2.92777455	0.68514436
H	-1.00784275	4.01844679	0.66274755
H	-2.10205798	2.69029753	0.17036792
H	-1.28087838	2.60934914	1.73603998

$(S_0/S_1)_{CI}^{90^\circ}$

C	0.60305665	-0.13035768	1.00944345
C	-0.01847692	0.73469003	0.04887178
C	-0.65439018	-0.11058595	-0.91215150
C	0.34291737	-1.49307864	0.62910410
C	-0.43419526	-1.49472410	-0.55613463
C	-0.00853786	2.20439939	0.04556582
H	-1.21866569	0.24848433	-1.77650130
H	1.17453435	0.19464596	1.88009987
H	-0.79208260	-2.36778906	-1.10101700
H	0.69266602	-2.37681686	1.16763555
C	1.09255004	2.93005248	-0.67345465
H	2.00224342	3.01236370	-0.04373733
H	1.38912241	2.38920612	-1.58745896
H	0.78714122	3.95259411	-0.94573027
C	-1.10635131	2.93445705	0.76561149
H	-0.95211407	4.02350414	0.73714889
H	-2.09227083	2.71197968	0.31380430
H	-1.17096336	2.61717510	1.82292639

$(S_0/S_1)_{CI}^{100^\circ}$

C	0.49725457	-0.11890535	1.05994327
C	-0.02266139	0.73830544	0.04101008
C	-0.55434733	-0.11144011	-0.96979886
C	0.27628361	-1.50231587	0.65845979
C	-0.36766221	-1.50285482	-0.58615880
C	-0.00744034	2.20583663	0.04386549
H	-1.02931115	0.22823130	-1.89283844
H	0.98792637	0.21849313	1.97549596
H	-0.69451789	-2.37202806	-1.15858263
H	0.57701919	-2.38067661	1.23172906
C	1.03869758	2.93572335	-0.74838385
H	1.97622312	3.04437739	-0.16568202
H	1.29865279	2.38200305	-1.66555179
H	0.70458756	3.94816259	-1.02549732
C	-1.04732820	2.93248365	0.84571729
H	-0.88979894	4.02118291	0.82675903
H	-2.06068518	2.71869371	0.45421204
H	-1.04670876	2.59492748	1.89932770

$(S_0/S_1)_{CI}^{110^\circ}$

C	0.38061587	-0.11777124	1.09794374
C	-0.02634138	0.73474172	0.03676014
C	-0.44929402	-0.11009038	-1.00953547
C	0.19408564	-1.52707242	0.67044109
C	-0.29847697	-1.52763316	-0.61525748
C	-0.00415623	2.21309774	0.04473627
H	-0.83749438	0.22707451	-1.97428102
H	0.77021958	0.21205114	2.06410201
H	-0.54896774	-2.38945007	-1.23454264
H	0.42517139	-2.39899943	1.28372269
C	0.98484201	2.94338042	-0.81630928
H	1.95507023	3.08765795	-0.29516076
H	1.20249297	2.38120279	-1.73811952
H	0.61193986	3.94351181	-1.08957029
C	-0.97990780	2.94099502	0.92128661
H	-0.85792173	4.03250936	0.85223113
H	-2.02221547	2.68738491	0.64381431
H	-0.86347844	2.64760915	1.98176449

$(S_0/S_1)^{120°}_{CI}$

C	0.25038293	-0.12491688	1.12289526
C	-0.02901090	0.73303083	0.03645225
C	-0.33581991	-0.10673852	-1.03769576
C	0.10449209	-1.55828452	0.68112719
C	-0.22081509	-1.55234230	-0.63957921
C	0.00113157	2.23348620	0.04737316
H	-0.62884758	0.22601173	-2.03692430
H	0.53399998	0.19482674	2.12889831
H	-0.39235375	-2.40327411	-1.29892320
H	0.24945255	-2.42287807	1.33009680
C	0.93725297	2.95625554	-0.87467380
H	1.95377740	3.08865036	-0.44498129
H	1.06688414	2.40635710	-1.82049693
H	0.55253011	3.96187659	-1.11022979
C	-0.91515318	2.94993129	0.98902703
H	-0.88577828	4.03928771	0.83309535
H	-1.96164646	2.60463485	0.87266086
H	-0.64429522	2.75428529	2.04590409

$(S_0/S_1)^{130°}_{CI}$

C	0.12569731	-0.11858303	1.13006933
C	-0.03088989	0.72441055	0.01930907
C	-0.22190271	-0.11986564	-1.06302825
C	0.02220921	-1.57746041	0.68948011
C	-0.14933295	-1.58329505	-0.64545884
C	0.00819382	2.25364529	0.04326624
H	-0.41228351	0.19859722	-2.09195981
H	0.29611939	0.21052988	2.15890858
H	-0.25330744	-2.43423126	-1.31844490
H	0.07173245	-2.42950078	1.36792396
C	0.88485523	2.97920306	-0.93212397
H	1.94158484	3.07479404	-0.59693166
H	0.91472528	2.46743293	-1.90726231
H	0.50806177	4.00143475	-1.09717859
C	-0.84392655	2.94793913	1.05440807
H	-0.88635349	4.03313039	0.87098385
H	-1.87821362	2.54860316	1.05588796
H	-0.46078577	2.80341559	2.08617716

$(S_0/S_1)^{140°}_{CI}$

C	0.00638162	-0.10227444	1.14755841
C	-0.02630652	0.73029046	0.03552670
C	-0.05967280	-0.12373305	-1.05879226
C	-0.00768220	-1.58474303	0.71320635
C	-0.01121365	-1.59456347	-0.62469039
C	-0.02484025	2.28245047	0.04735049
H	-0.10908240	0.18397771	-2.10714557
H	0.03573251	0.22858389	2.18986142
H	0.00030862	-2.44608111	-1.30489118
H	-0.01323138	-2.42564048	1.40686209
C	0.79994492	2.96629280	-0.99154759
H	1.78856365	2.47613509	-1.10114470
H	0.32652629	2.92290380	-1.99576003
H	0.96380058	4.02980237	-0.75624131
C	-0.84077487	2.97238937	1.08714488
H	-0.88008714	4.05906799	0.91395563
H	-1.88205218	2.58958795	1.11632210
H	-0.43013144	2.81575351	2.10645096

$(S_0/S_1)^{150°}_{CI}$

C	-0.10664323	-0.09795160	1.13505460
C	-0.02632298	0.72533099	0.02800883
C	0.04864242	-0.12584493	-1.05960428
C	-0.08002237	-1.59656617	0.70867981

C	0.04382817	-1.60682657	-0.61692025
C	-0.02391048	2.30253846	0.04688464
H	0.12074761	0.17845573	-2.10709310
H	-0.18854613	0.23224661	2.17433322
H	0.12935936	-2.45619052	-1.29505329
H	-0.15223810	-2.43215632	1.40431982
C	0.72403689	2.97330421	-1.05045629
H	1.73061230	2.52314550	-1.18449077
H	0.21494003	2.87274941	-2.03312618
H	0.85227011	4.05044459	-0.86076090
C	-0.76261849	2.97534580	1.14967332
H	-0.78598110	4.06718439	1.00897526
H	-1.80827254	2.61383887	1.23685155
H	-0.29369814	2.78115139	2.13875003

$(S_0/S_1)^{160^\circ}_{CI}$

C	-0.21703186	-0.09856589	1.11289390
C	-0.02161409	0.72340075	0.02442291
C	0.16378332	-0.12693591	-1.04670178
C	-0.14954538	-1.60466095	0.69475353
C	0.09777163	-1.61562241	-0.60933996
C	-0.01942961	2.31862369	0.04968632
H	0.34757488	0.17673276	-2.08121476
H	-0.40352313	0.23512399	2.13831631
H	0.23371678	-2.46373949	-1.28018877
H	-0.28782166	-2.43642289	1.38506113
C	0.65046564	2.97816268	-1.09850519
H	1.66912207	2.56448746	-1.26525538
H	0.10390210	2.81914761	-2.05434086
H	0.74164275	4.06519349	-0.94773139
C	-0.68077876	2.97696775	1.20707348
H	-0.72403458	4.06870638	1.07029157
H	-1.71904689	2.61238188	1.35991759
H	-0.14896986	2.78721891	2.16488736

$(S_0/S_1)^{170^\circ}_{CI}$

C	-0.32269747	-0.10363180	1.08584525
C	-0.01852238	0.72359796	0.02947694
C	0.27613732	-0.12426067	-1.01799794
C	-0.21253260	-1.61216921	0.67677506
C	0.15167548	-1.61776457	-0.59752651
C	-0.01877691	2.32946408	0.05387931
H	0.56848344	0.18066295	-2.02669738
H	-0.60978616	0.22625840	2.08911909
H	0.34119672	-2.46288694	-1.25914604
H	-0.41291220	-2.44423207	1.35123875
C	0.56703141	2.97703197	-1.14346937
H	1.60237866	2.61783741	-1.33756442
H	-0.00589044	2.74638965	-2.06914894
H	0.60147975	4.07241429	-1.03803944
C	-0.59689081	2.98351646	1.25472873
H	-0.60647167	4.07862660	1.13986387
H	-1.64084994	2.65641026	1.45125723
H	-0.02686886	2.75293502	2.18143182

$(S_0/S_1)^{180^\circ}_{CI}$

C	-0.42343344	-0.09755136	1.05030634
C	-0.01270732	0.72236889	0.02617078
C	0.38585420	-0.12643635	-0.98388029
C	-0.27266420	-1.60891980	0.65645037
C	0.21084166	-1.61988427	-0.57635945
C	-0.01707560	2.33250626	0.05306294
H	0.78939465	0.17607351	-1.95439838
H	-0.80084173	0.23749685	2.02172056
H	0.45427634	-2.46694896	-1.21747361
H	-0.55269449	-2.43756097	1.30607890

C	0.47746659	2.97923224	-1.18342815
H	1.52451463	2.68451891	-1.42001657
H	-0.12226202	2.68124671	-2.07266287
H	0.44649550	4.07704195	-1.10680447
C	-0.50896792	2.97840555	1.29567008
H	-0.45892760	4.07518848	1.21220912
H	-1.56313091	2.70794301	1.52533424
H	0.08004498	2.68547915	2.19204649

References

[1] Toru Shiozaki, Clemens Woywod, and Hans-Joachim Werner. Pyrazine excited states revisited using the extended multi-state complete active space second-order perturbation method. *Phys. Chem. Chem. Phys.*, 15:262–269, 2013.

[2] James Finley, Per Åke Malmqvist, Björn O. Roos, and Luis Serrano-Andrés. The multi-state caspt2 method. *Chemical Physics Letters*, 288(2):299–306, 1998.

[3] Stefano Battaglia and Roland Lindh. Extended dynamically weighted caspt2: The best of two worlds. *Journal of Chemical Theory and Computation*, 16(3):1555–1567, 2020. PMID: 32027802.

[4] Stefano Battaglia and Roland Lindh. On the role of symmetry in XDW-CASPT2. *The Journal of Chemical Physics*, 154(3):034102, 01 2021.

[5] Lea M. Ibele, Arshad Memhood, Benjamin G. Levine, and Davide Avagliano. Ab initio multiple spawning nonadiabatic dynamics with different caspt2 flavors: A fully open-source pyspawn/openmolcas interface. *Journal of Chemical Theory and Computation*, 20(18):8140–8151, 2024. PMID: 39228232.

[6] Alexander A. Granovsky. Extended multi-configuration quasi-degenerate perturbation theory: The new approach to multi-state multi-reference perturbation theory. *The Journal of Chemical Physics*, 134(21):214113, 06 2011.

[7] Toru Shiozaki, Werner Győrffy, Paolo Celani, and Hans-Joachim Werner. Communication: Extended multi-state complete active space second-order perturbation theory: Energy and nuclear gradients. *The Journal of Chemical Physics*, 135(8):081106, 08 2011.

[8] L. Martínez-Fernández, A. J. Pepino, J. Segarra-Martí, J. Jovaišaitė, I. Vaya, A. Nenov, D. Markovitsi, T. Gustavsson, A. Banyasz, M. Garavelli, and R. Improta. Photophysics of deoxycytidine and 5-methyldeoxycytidine in solution: A comprehensive picture by quantum mechanical calculations and femtosecond fluorescence spectroscopy. *Journal of the American Chemical Society*, 139(23):7780–7791, 2017. PMID: 28513173.

[9] Saumik Sen and Igor Schapiro. A comprehensive benchmark of the xms-caspt2 method for the photochemistry of a retinal chromophore model. *Molecular Physics*, 116(19-20):2571–2582, 2018.

[10] Björn O Roos, Kerstin Andersson, Markus P Fülscher, Per-âke Malmqvist, Luis Serrano-Andrés, Kristin Pierloot, and Manuela Merchán. Multiconfigurational perturbation theory: Applications in electronic spectroscopy. *Advances in chemical physics: new methods in computational quantum mechanics*, 93:219–331, 1996.

[11] Stefano Battaglia, Ignacio Fdez. Galván, and Roland Lindh. Chapter 5 - multiconfigurational quantum chemistry: The caspt2 method. In Cristina García-Iriepa and Marco Marazzi, editors, *Theoretical and Computational Photochemistry*, pages 135–162. Elsevier, 2023.

[12] Rachel Crespo-Otero and Mario Barbatti. Recent advances and perspectives on nonadiabatic mixed quantum–classical dynamics. *Chemical Reviews*, 118(15):7026–7068, 2018. PMID: 29767966.

[13] Basile F. E. Curchod and Todd J. Martínez. Ab initio nonadiabatic quantum molecular dynamics. *Chemical Reviews*, 118(7):3305–3336, 2018. PMID: 29465231.

[14] Mario Barbatti. Velocity adjustment in surface hopping: Ethylene as a case study of the maximum error caused by direction choice. *Journal of Chemical Theory and Computation*, 17(5):3010–3018, 2021. PMID: 33844922.

[15] Josene M. Toldo, Rafael S. Mattos, Max Jr. Pinheiro, Saikat Mukherjee, and Mario Barbatti. Recommendations for velocity adjustment in surface hopping. *Journal of Chemical Theory and Computation*, 20(2):614–624, 2024. PMID: 38207213.

[16] Ignacio Fdez. Galván, Mickaël G. Delcey, Thomas Bondo Pedersen, Francesco Aquilante, and Roland Lindh. Analytical state-average complete-active-space self-consistent field nonadiabatic coupling vectors: Implementation with density-fitted two-electron integrals and application to conical intersections. *Journal of Chemical Theory and Computation*, 12(8):3636–3653, 2016. PMID: 27327873.

[17] Christopher S. Page and Massimo Olivucci. Ground and excited state caspt2 geometry optimizations of small organic molecules. *Journal of Computational Chemistry*, 24(3):298–309, 2003.

[18] Javier Segarra-Martí, Marco Garavelli, and Francesco Aquilante. Multiconfigurational second-order perturbation theory with frozen natural orbitals extended to the treatment of photochemical problems. *Journal of Chemical Theory and Computation*, 11(8):3772–3784, 2015. PMID: 26574459.

[19] Michael J. Bearpark, Fernando Bernardi, Massimo Olivucci, Michael A. Robb, and Barry R. Smith. Can fulvene s_1 decay be controlled? a casscf study with mmvb dynamics. *Journal of the American Chemical Society*, 118(22):5254–5260, 1996.

[20] LiLing Wang, Alireza Azizi, Tianlv Xu, Michael Filatov, Steven R. Kirk, Martin J. Paterson, and Samantha Jenkins. The role of the natural transition orbital density in the $s_0 \rightarrow s_1$ and $s_0 \rightarrow s_2$ transitions of fulvene with next generation qtaim. *Chemical Physics Letters*, 751:137556, 2020.
